# Phenotypic diversity and genotypic flexibility of *Burkholderia cenocepacia* during long-term chronic infection of cystic fibrosis lungs

**DOI:** 10.1101/gr.213363.116

**Published:** 2017-04

**Authors:** Amy Huei-Yi Lee, Stephane Flibotte, Sunita Sinha, Adrianna Paiero, Rachel L. Ehrlich, Sergey Balashov, Garth D. Ehrlich, James E.A. Zlosnik, Joshua Chang Mell, Corey Nislow

**Affiliations:** 1 Department of Microbiology and Immunology,; 2 Department of Pharmaceutical Sciences, University of British Columbia, Vancouver, British Columbia V6T 1Z3, Canada;; 3 Department of Zoology, University of British Columbia, Vancouver, British Columbia V6T 1Z4, Canada;; 4 Department of Microbiology and Immunology, Drexel University College of Medicine, Philadelphia, Pennsylvania 19102, USA;; 5 Genomics Core Facility, Clinical and Translational Research Institute, Drexel University College of Medicine, Philadelphia, Pennsylvania 19102, USA;; 6 Center for Genomic Sciences, Institute for Molecular Medicine and Infection Diseases, Drexel University College of Medicine, Philadelphia, Pennsylvania 19102, USA;; 7 Centre for Preventing and Understanding Infection in Children, BC Children's Hospital, University of British Columbia, Vancouver, British Columbia V5Z 4H4, Canada

## Abstract

Chronic bacterial infections of the lung are the leading cause of morbidity and mortality in cystic fibrosis patients. Tracking bacterial evolution during chronic infections can provide insights into how host selection pressures—including immune responses and therapeutic interventions—shape bacterial genomes. We carried out genomic and phenotypic analyses of 215 serially collected *Burkholderia cenocepacia* isolates from 16 cystic fibrosis patients, spanning a period of 2–20 yr and a broad range of epidemic lineages. Systematic phenotypic tests identified longitudinal bacterial series that manifested progressive changes in liquid media growth, motility, biofilm formation, and acute insect virulence, but not in mucoidy. The results suggest that distinct lineages follow distinct evolutionary trajectories during lung infection. Pan-genome analysis identified 10,110 homologous gene clusters present only in a subset of strains, including genes restricted to different molecular types. Our phylogenetic analysis based on 2148 orthologous gene clusters from all isolates is consistent with patient-specific clades. This suggests that initial colonization of patients was likely by individual strains, followed by subsequent diversification. Evidence of clonal lineages shared by some patients was observed, suggesting inter-patient transmission. We observed recurrent gene losses in multiple independent longitudinal series, including complete loss of Chromosome III and deletions on other chromosomes. Recurrently observed loss-of-function mutations were associated with decreases in motility and biofilm formation. Together, our study provides the first comprehensive genome-phenome analyses of *B. cenocepacia* infection in cystic fibrosis lungs and serves as a valuable resource for understanding the genomic and phenotypic underpinnings of bacterial evolution.

Cystic fibrosis (CF) is the most common fatal genetic disorder, arising due to mutations within the cystic fibrosis transmembrane conductance regulator (*CFTR*) gene, which alters ion fluxes in mucosal membranes, including the lung airways (Riordan et al. 1989; Cant et al. 2014). The thick mucus covering the CF airway is an ideal environment for a polymicrobial community, including pathogens such as *Pseudomonas aeruginosa*, *Staphylococcus aureus*, and the *Burkholderia cepacia* species complex (*Bcc*) (Filkins and O'Toole 2015; Parkins and Floto 2015; Huang and LiPuma 2016). The ecological diversity and the dynamics of this community complicate diagnosis and treatment (Rau et al. 2012). Aggressive antimicrobial therapies are typically used to treat recurrent infections (Bhatt 2013; Biller 2015), and while effective at relieving symptoms, they encourage evolutionary adaptation of bacterial populations, including the development of antimicrobial resistance (Griesenbach and Alton 2015). *B. cenocepacia* infection is a risk factor for CF patients (Lipuma 2010), and because it is both highly transmissible and often antibiotic resistant, its presence can exclude patients from lung transplantation (Murray et al. 2008; Lobo et al. 2015).

The unpredictable infection trajectory of *B. cenocepacia* infection in CF patients poses treatment challenges; some patients colonized by *B. cenocepacia* are asymptomatic, while others develop cepacia syndrome, which can be fatal (Isles et al. 1984). CF patients infected with genotypically similar *Burkholderia* strains can have drastically different clinical outcomes. While useful for tracking *Bcc* epidemics, current genotyping methods—such as pulse-field gel electrophoresis (PFGE), multilocus sequence typing (MLST), and PCR-based methods with randomly amplified polymorphic DNA (RAPD) or BOX-PCR (Govan et al. 1993; Jones et al. 2004)—are not sufficient to define the scope of genetic diversity in infectious *B. cenocepacia* strains or to identify the underlying genetic differences responsible for variation in bacterial phenotypes that influence clinical outcomes (Mahenthiralingam et al. 2000; Speert et al. 2002a; Vonberg et al. 2006; Pretto et al. 2013). These low-resolution genotyping methods have shown that long-term sequential isolates from one patient typically belong to the same clonal type but nevertheless show phenotypic diversity (Cunha et al. 2003; Coutinho et al. 2011a,b).

Whole-genome sequencing provides single-base resolution of bacterial evolution in CF lung infections (Didelot et al. 2016). Similar approaches in *P. aeruginosa*, *Burkholderia dolosa*, and *Burkholderia multivorans* have shown that mutations accumulate in clonal lineages during adaptation to the lung. The spatial heterogeneity in CF airways can also influence bacterial population diversity, suggesting deep sampling is needed to capture this diversity (Lieberman et al. 2014; Markussen et al. 2014). These studies also find parallel adaptive changes in specific molecular pathways in response to host selection pressures, including antibiotic resistance, and changes in bacterial cell wall and membrane composition, metabolism, and oxygen-sensing (Smith et al. 2006; Huse et al. 2010; Lieberman et al. 2011, 2014; Yang et al. 2011; Marvig et al. 2013, 2015; Silva et al. 2016).

In contrast to *P. aeruginosa* and *B. dolosa*, little is known about the genomic changes that occur in *B. cenocepacia* in CF nor about how such changes influence clinically relevant bacterial phenotypes. *B. cenocepacia* is the most prevalent *Bcc* species colonizing CF patients, with most isolates from the United Kingdom and Canada belonging to a single molecular type (electrophoretic type ET12 or RAPD02) that includes epidemic strain J2315 (Speert et al. 2002b; McDowell et al. 2004). Along with RAPD02; RAPD01, -04, and -06 comprise a distinct genomic group (previously genomovar IIIA, now called *recA* subgroup A), whereas other RAPD types belong to a divergent *B. cenocepacia* genomic group (previously genomovar IIIB, now *recA* subgroup B) that includes PHDC epidemic lineages HI2424 and AU1054 strains (Johnson 1994; Rozee et al. 1994; Mahenthiralingam et al. 1996; Speert 2002; Speert et al. 2002a; Lipuma 2005; Holden et al. 2009; Miller et al. 2015).

*B. cenocepacia* was the most common species found in infected patients between 1990 and 1995 in clinics in Vancouver, Canada (Zlosnik et al. 2015). To understand how *B. cenocepacia* evolved in these patients, we selected 215 isolates from 16 patients over a span of 2.3–20.7 yr, because they included numerous isolates per patient and covered the diverse molecular types representing the major epidemic lineages. We conducted whole-genome sequencing and a battery of phenotypic assays (growth, motility, biofilm, mucoidy, and acute virulence) on all 215 isolates to gain insight into the genomic and phenotypic diversity within and between *B. cenocepacia* molecular types. Our results show patterns of recurrent phenotypic and genomic changes in independent *B. cenocepacia* infections of distinct molecular types, and this new data set offers a comprehensive genomic and phenotypic resource for future work on understanding bacterial evolution in the CF lung.

## Results

### A longitudinal collection of *B. cenocepacia* bacterial isolates

**Figure 1.**
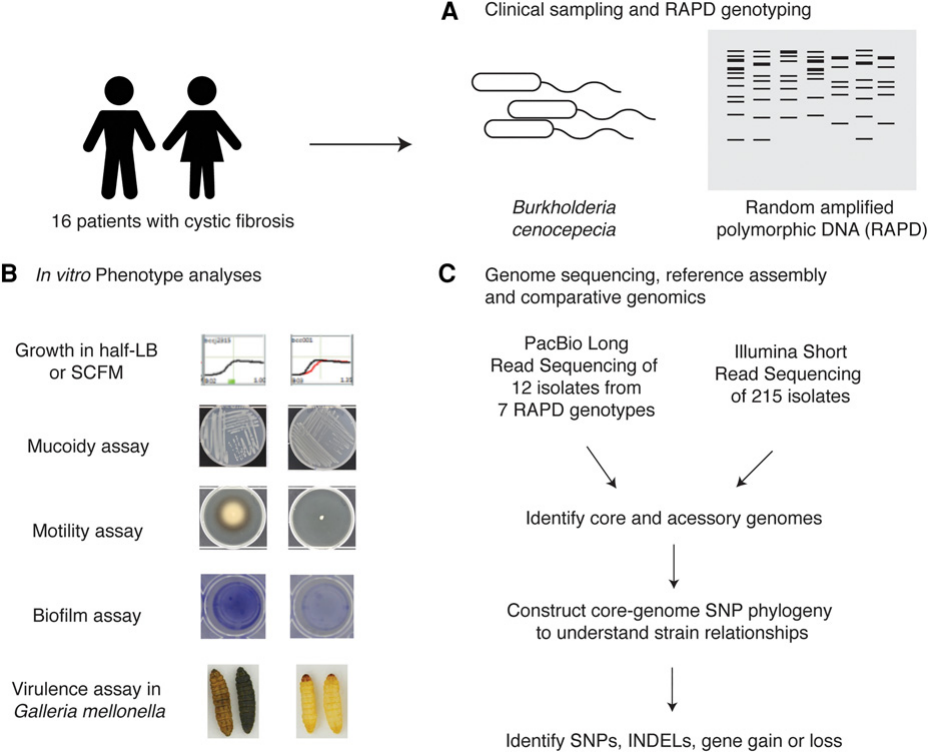
Overview of data collection. (*A*) To understand the genomic and phenotypic evolution of *B. cenocepacia* strains within the CF lung, we examined 16 longitudinal series of *B. cenocepacia* strains isolated from sputum (215 isolates total) that had been collected and typed using RAPD analysis as part of the surveillance program at CBCCRRR. (*B*) In vitro phenotypic analyses were carried out for all isolates, focusing on clinically relevant traits: growth rate, motility, biofilm formation, mucoidy, and acute virulence in an insect model system. (*C*) Short-read paired-end sequencing by Illumina was carried out for all 215 isolates. To provide reference-quality sequences for a subset of isolates representing all seven RAPD genotypes, long-read sequencing by PacBio was carried out on 11 isolates as well as on the reference *B. cenocepacia* J2315 as a control.

We used *Burkholderia* isolates collected from CF patients by the CBCCRRR (Canadian *Burkholderia cepacia* Complex Research and Referral Repository) (Fig. 1; Zlosnik et al. 2015). *Bcc* isolates were typed by RAPD analyses, which used random primers to generate electrophoretic patterns that differ among divergent isolates (Mahenthiralingam et al. 1996; Speert et al. 2002a).

**Figure 2.**
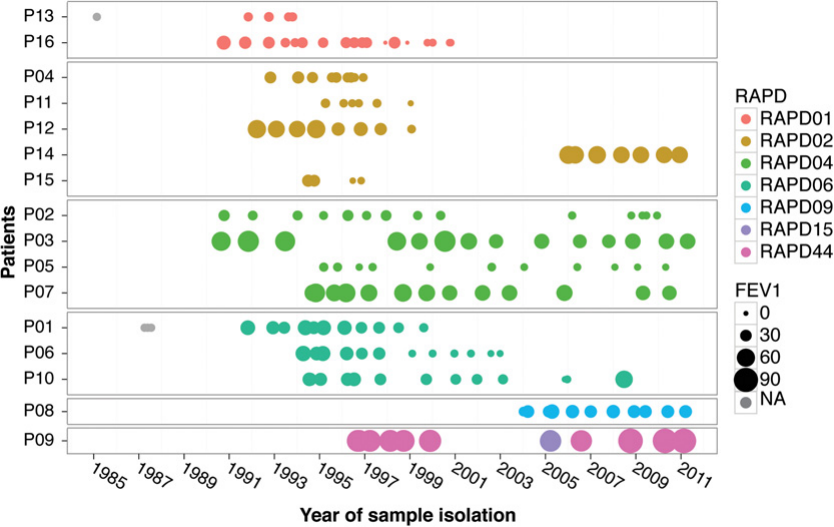
Longitudinal series of *B. cenocepacia* isolates. Each series (collected from patients P01 to P16) is depicted as a row of dots to represent sampling time points. Colors indicate different RAPD genotypes, and relative dot size indicates patient lung function (%FEV_1_) at that time point. Gray circles indicate no associated %FEV_1_ (percentage of predicted forced expiratory volume in 1 sec) measurement (also see Supplemental Fig. S1).

To develop a comprehensive genomic and phenotypic view of *B. cenocepacia* evolution in the CF lung, we selected 215 isolates from 16 patients from the CBCCRRR between 1985 and 2011 (Table 1; Figs. 1, 2; Supplemental Table S2). Selection criteria included patient series with numerous longitudinally sampled isolates (range, seven to 23) and with all major epidemic lineages. For most time points, a single bacterial isolate was archived, although two to three independent isolates were archived for some time points (due to visual identification of distinct colony morphologies). Patient lung function was assessed by spirometry at most time points. Most patients exhibited progressive declines in lung function over time; 14 of 16 showed significant decreases for at least one of three measures of lung function (Fig. 2; Supplemental Fig. S1; Supplemental Table S1A–C). Declines were substantial; %FEV_1_ (percentage of predicted forced expiratory volume in 1 sec) decreased by an average of 7.5%/yr for 12 patients (Supplemental Table S1A). Two additional measures of lung function gave similar results (Supplemental Table S1B,C).

**Table 1.**
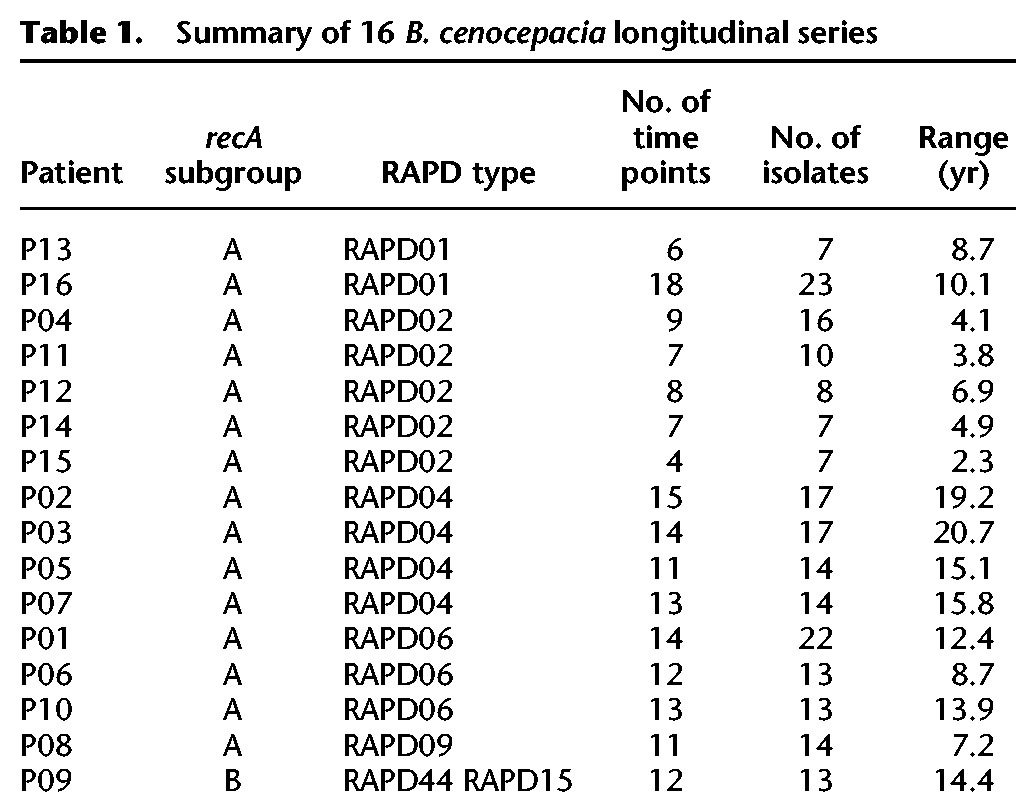
Summary of 16 *B. cenocepacia* longitudinal series

The majority of patients were colonized by subgroup A isolates (202 of 215 isolates) primarily from one of the major *B. cenocepacia* epidemic lineages. Except for the single patient P09 infected with subgroup B, all isolates from the same patient belonged to the same RAPD type. By using these 215 isolates, we conducted a comprehensive survey of clinically relevant phenotypes and carried out whole-genome sequence analysis of all isolates.

### Phenotypic analysis of *B. cenocepacia* longitudinal series finds strong correlations with RAPD type and frequent progressive temporal changes

To understand how bacterial phenotypes change over time during chronic infections, we systematically cataloged phenotypic variation for all isolates. Specifically, we measured: growth in two liquid media types (lysogeny broth [LB]; and synthetic cystic fibrosis medium [SCFM]), swimming motility, biofilm formation, acute virulence in an insect model, and mucoidy morphotype (Palmer et al. 2007). The results reveal extensive phenotypic variation among *B. cenocepacia* isolates both within and among RAPD types and within patient series. Detailed descriptions and results for each phenotype are in Supplemental Text S1, Supplemental Table S1, and Supplemental Figures S2 and S3.

By considering these phenotypes on a global level, we found that RAPD genotype was a significant predictor of phenotypic variation, explaining between ∼20% and 50% of the variation (Table 2; Supplemental Fig. S4). However, considerable variation was also seen within patient series and within RAPD type. We also identified progressive phenotypic changes in many longitudinal series (Table 3). Six of the 16 series showed progressive decreases in motility, five showed temporal changes in biofilm formation (two increasing and three decreasing), two showed decreases in acute virulence as measured in an insect model, none showed changes in mucoidy, and, finally, seven showed changes in one or more growth parameters.

**Table 2.**
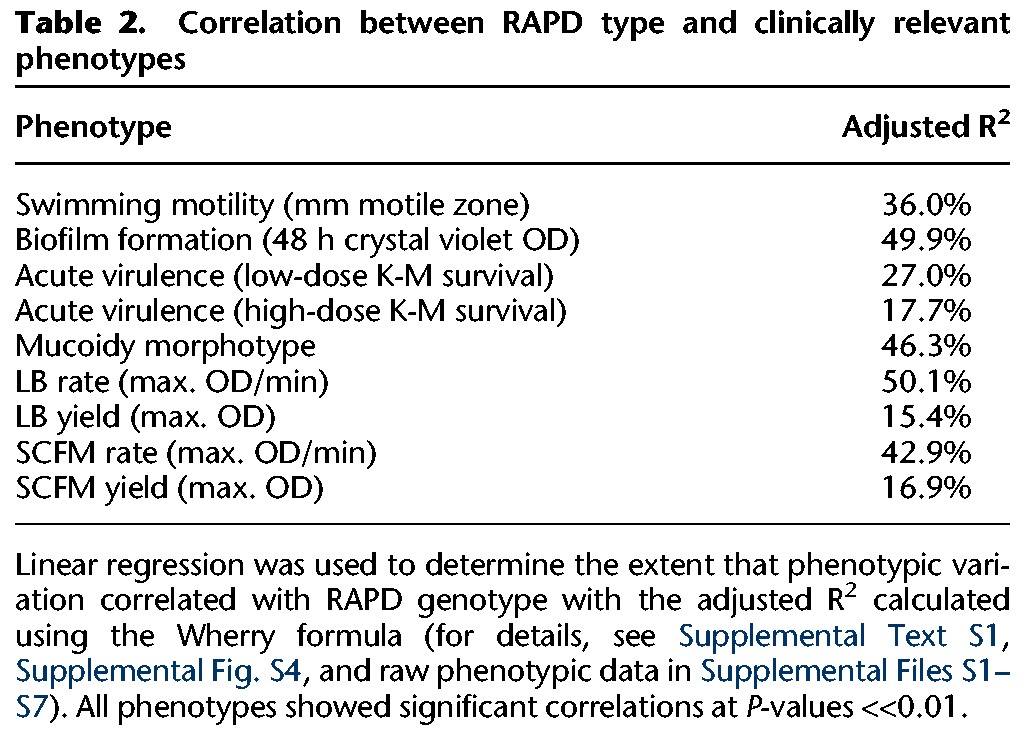
Correlation between RAPD type and clinically relevant phenotypes

Linear regression was used to determine the extent that phenotypic variation correlated with RAPD genotype with the adjusted R^2^ calculated using the Wherry formula (for details, see Supplemental Text S1, Supplemental Fig. S4, and raw phenotypic data in Supplemental Files S1–S7). All phenotypes showed significant correlations at *P*-values <<0.01.

**Table 3.**
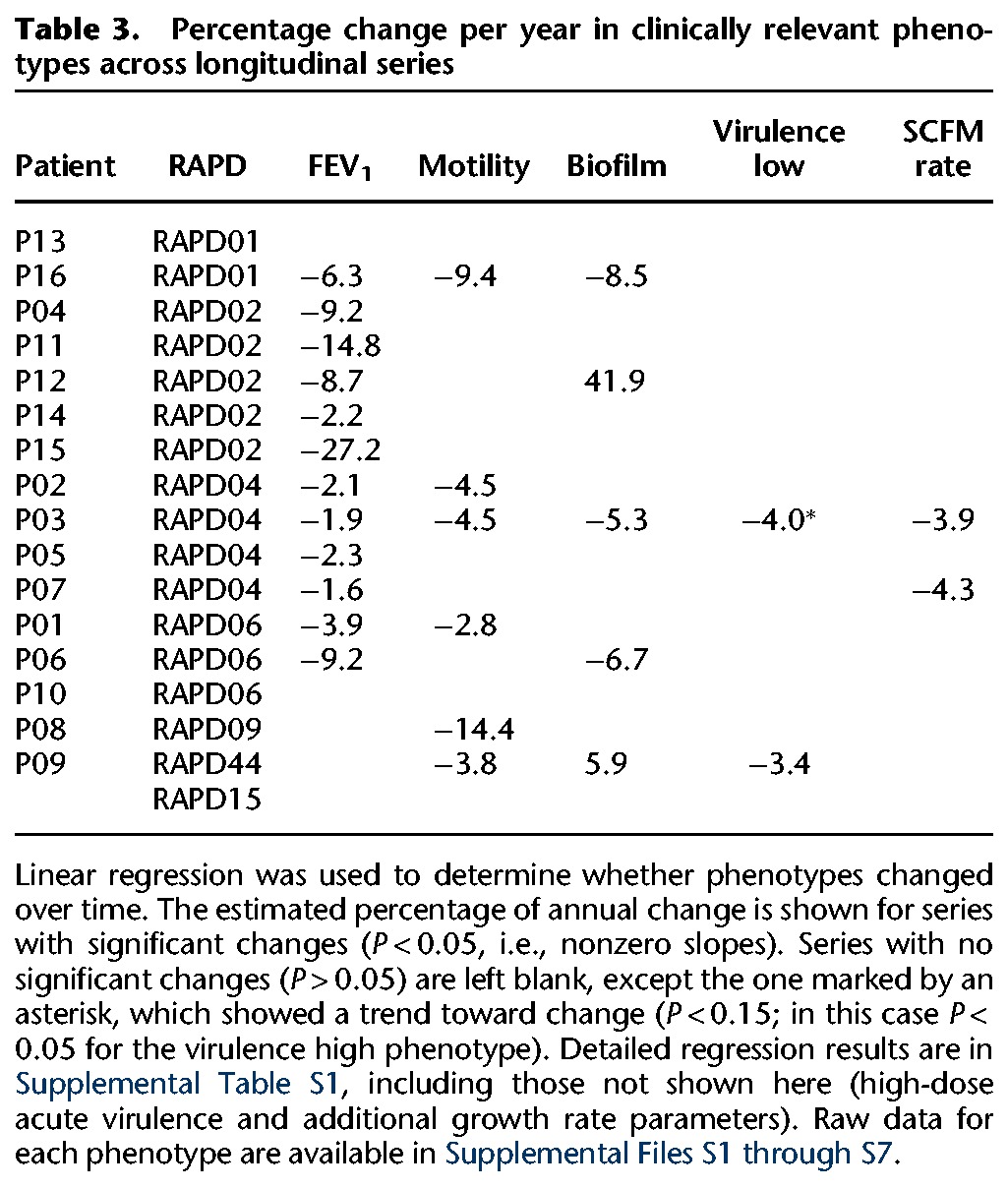
Percentage change per year in clinically relevant phenotypes across longitudinal series

**Figure 3.**
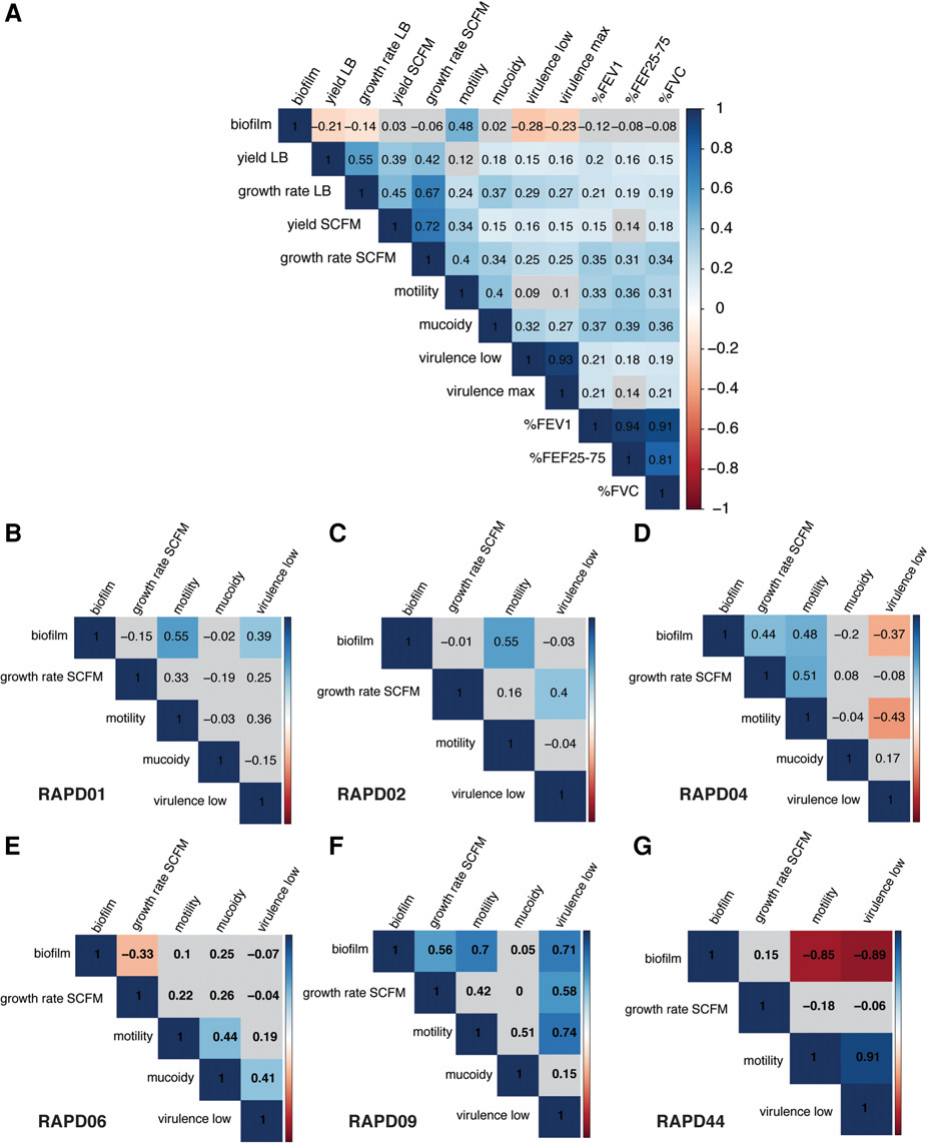
Correlation matrices of *B. cenocepacia* phenotypes and patient lung function. Pairwise Spearman rank correlations of the five phenotypes are depicted as matrices for the following: (*A*) all isolates, (*B*) RAPD01, (*C*) RAPD02, (*D*) RAPD04, (*E*) RAPD06, (*F*) RAPD09, and (*G*) RAPD44. Blue gradients indicate significant positive correlation; red gradients, significant negative correlation; and gray, statistically nonsignificant correlation and *P*-value >0.05.

Given that each individual phenotype could have a complex genetic basis, we examined pairwise phenotypic correlations across all isolates and for each RAPD type individually (Fig. 3). In general, most phenotypes we measured were positively correlated; with the exception that biofilm formation was negatively correlated with acute insect virulence and growth in LB (Fig. 3A). This is not surprising; biofilm has been associated with persistent infection and not with acute virulence (Furukawa et al. 2006; Wu et al. 2015). Overall, motility was positively correlated with biofilm formation and mucoidy (Spearman correlation, ρ = 0.48 and 0.40, respectively, adjusted *P*-value <0.0001) (Fig. 3A). A strong positive correlation was found between motility and biofilm formation for most RAPD genotypes, except for a negative correlation was seen for subgroup B RAPD44 isolates (ρ = −0.85, adjusted *P*-value = 0.001) (Fig. 3B–G).

Our phenotypic analyses showed that strains of the same RAPD type are more phenotypically similar and a trend over time toward decreasing motility, biofilm formation, acute virulence, and growth rate in many patients. These results likely represent a minimum estimate of phenotypic changes over time, since the single strain isolated at most time points may be one of multiple strains that coexist in the same infection. In support of this, for 36 time points for which two to four independent clones were available, seven patients had isolate pairs that showed significant differences in biofilm formation among isolates within a time point (*P* << 0.05). This strongly indicates that, even if most infections have a single clonal origin (as suggested from the RAPD typing results), strains generated by subsequent diversification can coexist in the same infection.

### Genome sequencing and assembly of *B. cenocepacia* longitudinal series

To identify genomic changes accumulating during chronic infection, we sequenced all 215 isolates to more than 60-fold coverage. *Burkholderia* genomes are relatively large (∼8 Mb) and repetitive, with two to three circular chromosomes and one or more plasmids (Holden et al. 2009). This complicated genome structure posed a challenge to genome assembly, especially in light of the dearth of high-quality reference sequences for all the RAPD types. We therefore supplemented Illumina sequencing for 11 isolates using long-read Pacific Biosciences RSII (PacBio) sequencing to about 60-fold genomic coverage. These 11 include four RAPD01 isolates; two isolates each of RAPD04, RAPD06, and RAPD09; and one isolate each for RAPD15 and -44 (Supplemental Table S4). As a control, we also resequenced the reference strain J2315 (Holden et al. 2009) using both short- and long-read methods. De novo assemblies were performed using the Ray assembler (Illumina) (Boisvert et al. 2010) and HGAP assembler (PacBio) (Chin et al. 2013), as detailed in the Methods.

Comparison of Illumina and PacBio assemblies from the same strains, methylation motifs identified by PacBio, and evaluation of the J2315 reference strain are included as Supplemental Files S8 and S9, Supplemental Text S2, Supplemental Table S5, and Supplemental Figure S5. Both sequence platforms gave consistent assemblies with only few single-nucleotide variants and structural differences (Supplemental Text S2; Supplemental Table S3). In all cases, however, the PacBio assemblies were of higher quality than their corresponding Illumina assemblies. Illumina assemblies had a median of 170 contigs and a median N50 of 97,307 bp, whereas the PacBio assemblies comprised one to 17 contigs and a median N50 of 3,775,012 bp (Supplemental Tables S3A,B). The pair-wise average nucleotide identities are >95% between *recA* subgroup A and B strains and >99% within each *recA* subgroup (Supplemental Files S8, S9; Supplemental Fig. S6).

The PacBio assembly identified Bcc129 as having a single circular chromosome with gene content from all three reference chromosomes (of *B. cenocepacia* HI2424 strain), suggesting a three-chromosome fusion, which was confirmed by PCR analyses (Supplemental Text S2; Supplemental Fig. S7). While the mechanism underlying this unique genome structure requires further study, the fusion implies a loss of function in two of the chromosome-specific ParAB partitioning systems (Dubarry et al. 2006).

### Pan-genome analyses identified clade-specific gene content in *B. cenocepacia*

The “pan-genome” or “supragenome” comprises all genes across members of a species, including both core genes (shared by all strains in a species) and “accessory” or “distributed” genes found only in subsets of strains (Medini et al. 2005; Shen et al. 2005; Hogg et al. 2007). Patterns of gene loss or gain across strains can be inferred from these inventories. Toward this end, we annotated each genome assembly using Prokka (Seemann 2014) and used Roary (Page et al. 2015) to cluster orthologous coding sequences and generate a gene possession matrix (assemblies with more than 500 contigs were excluded). This identified 2148 orthologous clusters present in >99% of 209 strains (core genes), along with an additional 36,627 present in only a subset of strains (Table 4; Supplemental Text S3; Supplemental Fig. S8). By collapsing together orthologous clusters that had been separated due to local gene order (see Methods), we arrived at an overall size of the *B. cenocepacia* pan-genome as consisting of 3005 core homologous gene clusters and 10,110 homologous gene clusters present in only a subset of strains (Table 4; Supplemental File S11).

**Table 4.**
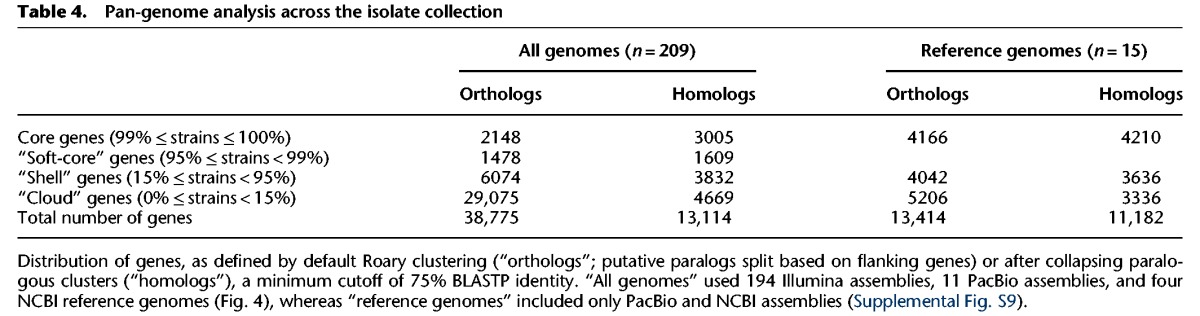
Pan-genome analysis across the isolate collection

**Figure 4.**
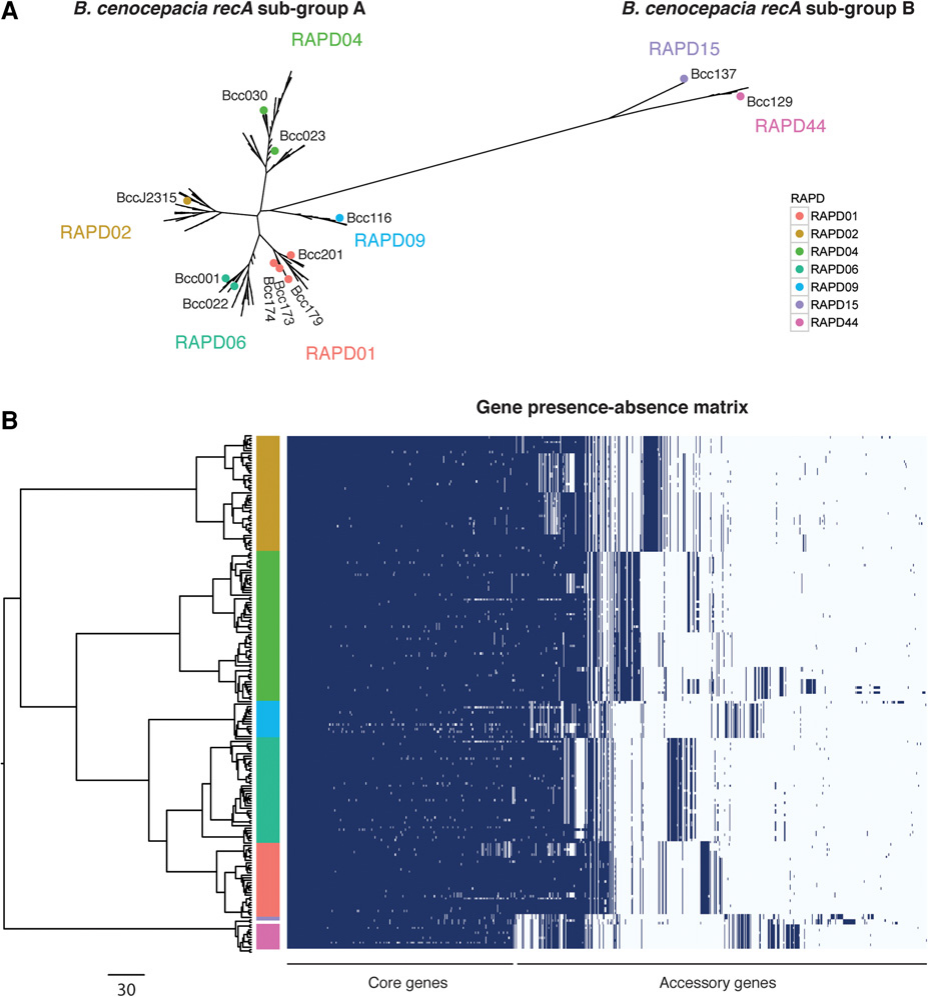
Core gene phylogeny and gene presence across *B. cenocepacia* isolates. (*A*) Core genome phylogeny built using RAxML (Stamatakis 2014) from 2148 orthologous clusters of protein coding genes from 209 *B. cenocepacia* isolates (four NCBI references were included, and 10 Illumina assemblies with more than 500 contigs were excluded). The structure shows that the RAPD genotypes form monophyletic clades. Colored dots correspond to RAPD reference genomes sequenced using PacBio and represent at least one isolate from each epidemic lineage. (*B*) *Left* side shows the same phylogenetic tree based on a concatenated core gene alignment. The *right* side shows a gene possession matrix, with each row representing a strain's gene content. Each column corresponds to a homologous gene cluster (i.e., after merging “orthologous” clusters into homologous clusters), and columns are ordered by the frequency of gene presence. The results clearly indicate extensive clade-specific gene content. The colored bar indicates RAPD genotype. Scale bar for the phylogenetic tree on the *bottom left* represents the number of SNPs in core genes.

A phylogenetic tree built from a concatenated core gene alignment of the 2148 unambiguous orthologs from all 209 strains showed that subgroups A and B formed highly differentiated clades (Fig. 4A; Vandamme et al. 1997; Mahenthiralingam et al. 2001). Isolates from each of the RAPD genotypes grouped into monophyletic clades, with RAPD06 more closely related to RAPD01, and RAPD02 more closely related to RAPD04 (Fig. 4A). Inspection of the core gene phylogeny (built from orthologous clusters only) (Fig. 4B, left) with the gene possession matrix (based on homolog clustering) (Fig. 4B, right) further underlined that RAPD types formed monophyletic groups and that large sets of accessory genes were RAPD specific (Fig. 4B; Supplemental Text S3).

### Evidence for diversification of *B. cenocepacia* clonal lineages during long-term infection of CF lungs

**Figure 5.**
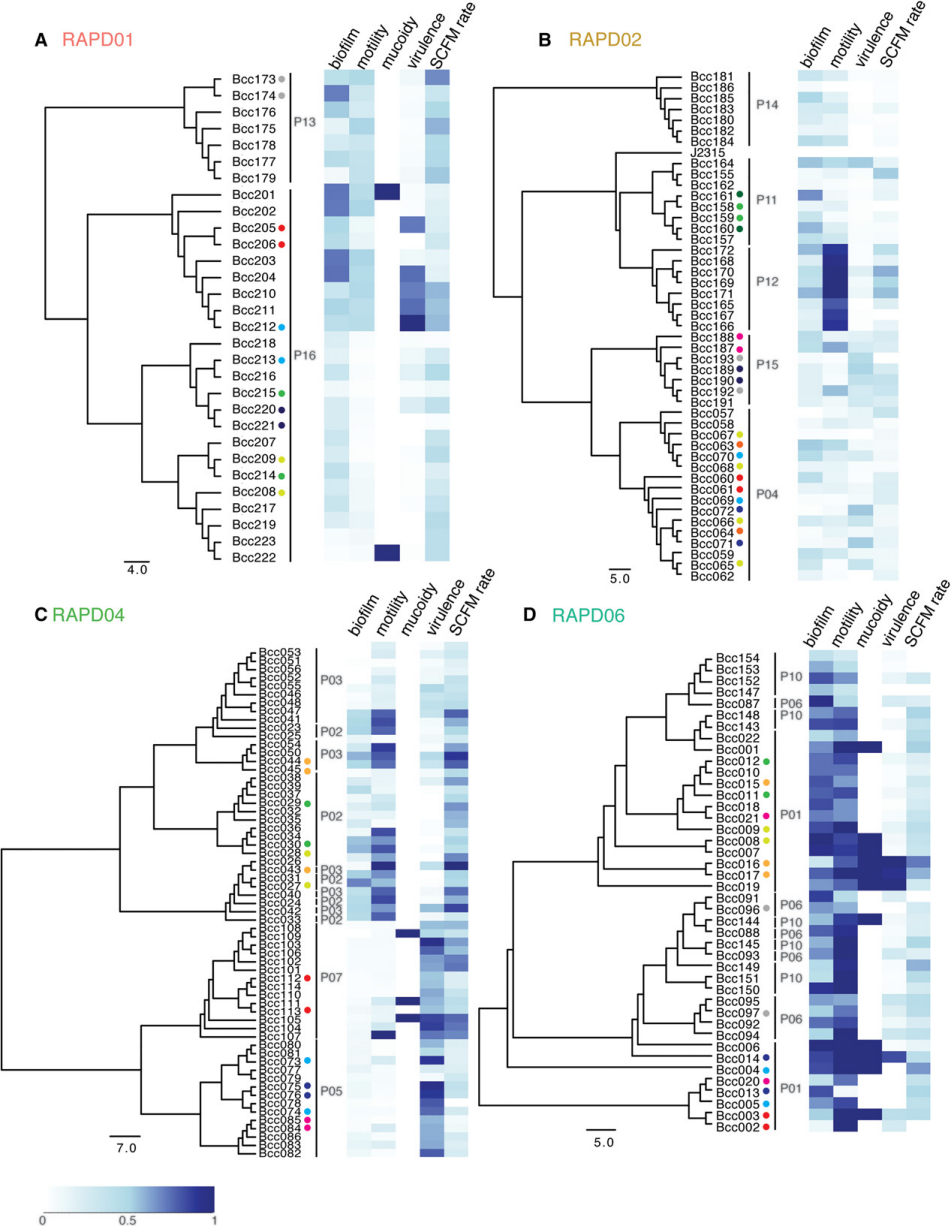
Genetic and phenotypic diversification of *B. cenocepacia* longitudinal series. Core gene phylogenetic trees were built for each RAPD type, with an adjacent heatmap for each phenotype. Phenotypic data were normalized to range between zero and one (lightest to darkest blue) for each phenotype, based on the range of values measured. Isolates: (*A*) RAPD01, (*B*) RAPD02, (*C*) RAPD04, and (*D*) RAPD06. Scale bar for the heatmap is on the *bottom left*, and scale bars for each phylogenetic tree represent the number of SNPs in core genes of the specific RAPD genotypes and the outgroup. Small colored dots are used for the 36 time points for which two to three isolates were collected (78 samples in total), with each color indicating a set of strains isolated from sputum at the same time point from that patient.

The retrospective nature of this study constrained our ability to associate genotype with phenotype. Building phylogenetic trees of each RAPD type based on their specific core gene orthologs and mapping the phenotypes of the corresponding strains to each tree (Fig. 5; Supplemental Fig. S10) can, however, illuminate the relationship between bacterial phylogeny, time of isolation, phenotype, and patient outcome (Supplemental Table S6; Supplemental Figs. S11, S12). Building trees for each RAPD type alone allowed inclusion of more core genes for a higher resolution in the phylogenetic analysis. We observed that isolates from RAPD01 and RAPD02 formed patient-specific clades (Fig. 5A,B; Supplemental Figs. S11, S12). This could suggest initial colonization of patients by single strains that subsequently diversify; alternatively, this pattern could be due to repeated colonizations by closely related isolates. Our results are consistent with observations from a *B. dolosa* outbreak at Boston Children's Hospital, in which isolates also formed distinct patient-specific clades (Lieberman et al. 2011). The phylogenetic structure of each patient-specific clade supports the presence of multiple strains from each clonal lineage coexisting in the same infection. For example, isolates from P16 fall into three different clades, and these isolates’ time of isolation has little or no correlation with clade structure (Fig. 5A; Supplemental Fig. S11A; Supplemental Table S2). Furthermore, two strains isolated at the same time (Fig. 5, colored dots; Supplemental Figs. S11, S12; Supplemental Table S2) often belonged to distinct clades; for example, isolates Bcc205/206 and Bcc220/221 are from sister taxa, while isolates Bcc212/213 and Bcc214/215 fall in distinct patient-specific phylogenies. This pattern can be seen over time for other isolates (e.g., P04: Bcc063/064). In contrast, for patient P14 (Fig. 5B), who developed cepacia syndrome, the single blood isolate (Bcc186) in the collection shared a recent common ancestry with sputum isolates (Bcc180–185), suggesting that the lung isolate entered the patient's bloodstream during sepsis and arose from the same original colonization event (Fig. 5B; St Denis et al. 2007).

We also observed cases where the same clonal lineages were shared among patients (for further discussion, see Supplemental Text S3). For RAPD04, isolates from patients P05 and P07 formed their own clades, while isolates from patients P02 and P03 were mixed (Fig. 5C; Supplemental Fig. S12A). Similarly, RAPD06 isolates were polyphyletic with respect to patient (P01, P06, and P10) (Fig. 5D; Supplemental Fig. S12B). As described above, strains isolated on the same date fall into distinct parts of the phylogenetic trees, showing coexistence of multiple strains (e.g., P02: Bcc043/044/045; P06: Bcc096/097). This intermingling of clonal lineages between patients suggest either that patients were independently infected by closely related strains or that strains were transmitted between patients.

In patient P09, we observed coinfection by two distinct subgroup B strains, showing that the strains’ positions in the patient-specific phylogeny were not correlated to when it was isolated (Fig. 1D; Supplemental Fig. S10B). Further evidence of coexisting strains belonging to the same clonal lineage comes from paired isolates Bcc129/130, which belong to distinct clades despite being sampled at the same time (Supplemental Fig. S10B).

Phenotypic variation among *B. cenocepacia* isolates collected from the same patient was extensive; for instance, we observed that 11 patients had isolates with significant phenotypic variation in motility (*P* << 0.05). This trend holds true even for isolates that show up as closely related sister taxa in the phylogenetic trees (e.g., P16: Bcc205 and Bcc206 for acute virulence, Bcc222 and Bcc223 for mucoidy; P11: Bcc158 and Bcc161 for biofilm; P01: Bcc013 and Bcc020 for motility). In fact, the branch lengths separating these sister taxa are often very short, with sister taxa separated by only a few nucleotides (see Fig. 5, scale bars), potentially suggesting that a small number of nucleotide differences may be responsible for large phenotypic differences. However, because these trees were based on only allelic variation in RAPD-specific core genes (Supplemental Table S6), a much more likely scenario is that phenotypic changes are the result of gene possession differences not reflected by these branch lengths.

### Recurrent genomic changes in *B. cenocepacia* during long-term infection of CF lungs

Previous studies on long-term bacterial adaptation have reported genome reduction by deletion of genes encoding nonessential functions, particularly for environmental-opportunistic pathogens (Rau et al. 2012; Price et al. 2013; Sharma et al. 2014). Long-term mutation accumulation studies showed that *B. cenocepacia* has low genome-wide mutation rates, with many of the mutations biased toward deletions (Dillon et al. 2015). Longitudinal sampling spanning up to 10 yr allowed us to ask if genome reduction occurs during the adaption of *B. cenocepacia* in CF patients. Specifically, we identified gene losses by comparing the gene content of later isolates to the first isolate within each longitudinal series.

**Figure 6.**
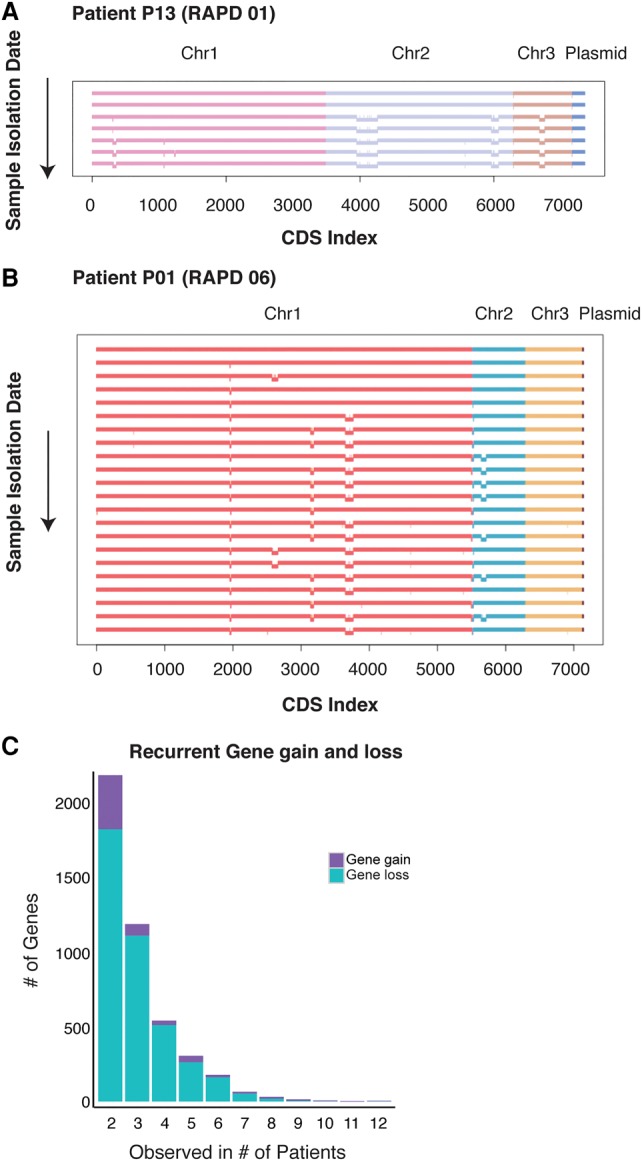
Recurrent gene loss observed in *B. cenocepacia* longitudinal series. Each horizontal line represents the genome of one isolate from patient P13 (*A*) and patient P01 (*B*). Each isolate genome is arranged chronologically, with the earliest isolate at the *top* and the last isolate at the *bottom* of the figure. Each replicon is represented by a different color. Genome deletions are represented by the offset bars *below* each chromosome. (*C*) Histogram of number of recurrent gene loss (teal) and gene gain (purple) observed in two or more patients.

Genome reduction was observed in seven patient series (P01, P02, P03, P07, P08, P13, and P16); isolates from later time points had reduced genomes and fewer genes versus earlier isolates (Supplemental Table S3; and examples in Fig. 6). Two strains (Bcc030 and Bcc115) lost Chromosome 3 entirely. Comparing the genome sizes for PacBio assemblies for early and late time points (patients P01, P02, and P13) also showed reduction in genome size and gene content (Supplemental Table S3B; Supplemental Fig. S13). Many gene losses likely occurred simultaneously as parts of large deletions (Supplemental Fig. S13).

By using the Roary gene presence matrix (homologous clusters) (Page et al. 2015), we found 8409 genes lost from at least one longitudinal series (Fig. 6C). We identified recurrent deletion of 3964 genes from at least two patient series over time, and 509 genes were lost in at least five patient series (Fig. 6C; Supplemental File S12). It is important to note that polarizing gene losses based on the earliest sampled isolate may not always be appropriate, since it might not reflect the most “ancestral” genome. However, we observed recurrent gains in substantially fewer patient series than losses, 572 gains in two series and 102 genes in only five series (Supplemental File S13); this suggests that choosing the earliest isolated strain was usually appropriate. Highly recurrent losses include putative virulence genes such as *aiiA* (group_8669; N-acyl homoserine lactonase), *mdtO* (group_10148; multidrug resistance), *luxO* (group_5898) and *gmr* (group_19364; cell–cell communication), and *hrp1* (group_24751; hypoxic response), while the majority of recurrent gene losses were hypothetical proteins (Fig. 6A,B; Supplemental File S12).

### Genetic diversity contributes to phenotypic diversity

**Figure 7.**
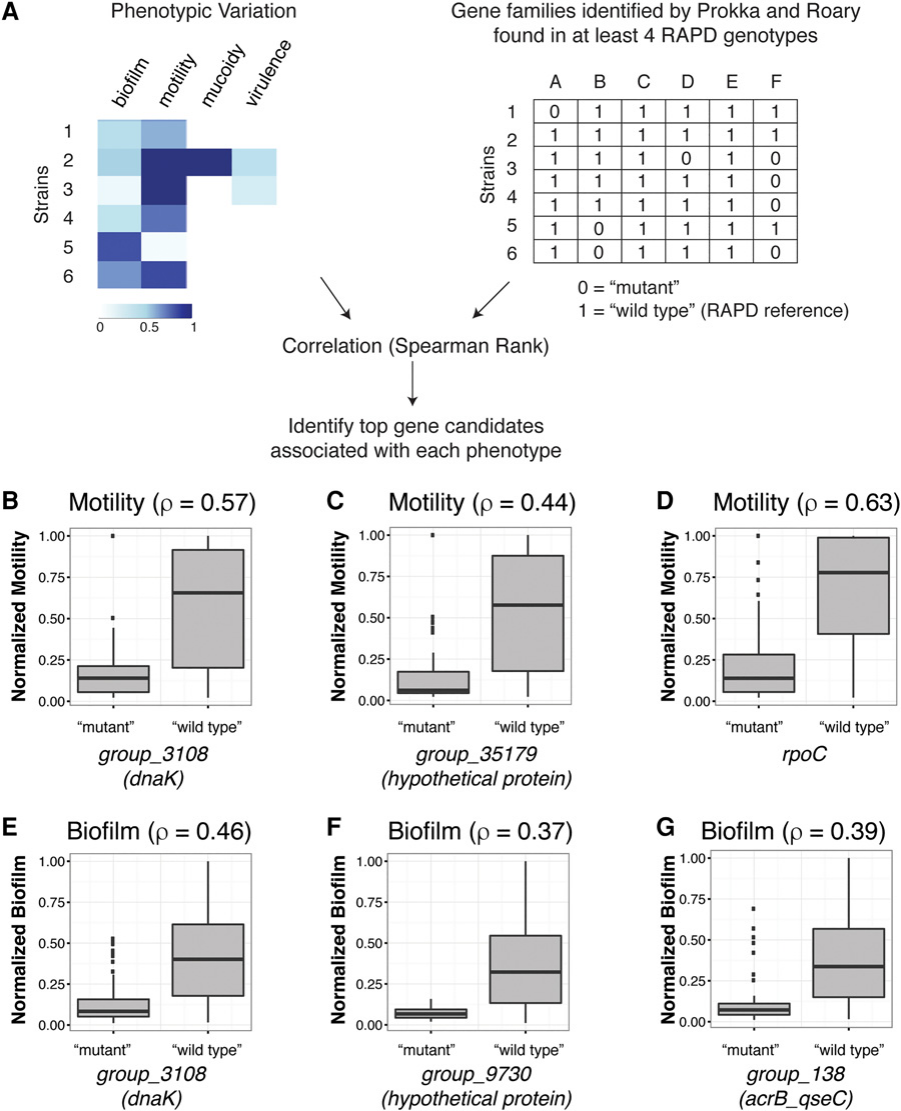
Genetic association testing identifies candidate gene families for the observed phenotypic variation. (*A*) We generated two matrices, one representing the phenotypic variation and the other the genotypic variation where we defined the variation for each isolate with respect to its RAPD reference, with 1 = “wild type” and 0 = “mutant,” i.e., any allele affected by deletion, nonsense, or missense mutations compared with the appropriate RAPD PacBio reference sequence from the earliest time point. Spearman rank correlation found genes that were highly correlated with either motility (examples in *B*–*D*) or biofilm (examples in *E*–*G*).

The phenotypic variation among isolates from the same patient (Fig. 5) must reflect underlying genetic (or epigenetic) variation. To test for associations between genetic variants and phenotypic differences, we used the Spearman-ranked correlation for motility and biofilm formation. We simplified our data into two matrices: (1) a binary genotype matrix defining the allele of each protein-coding gene in each strain as “wild type” or “mutant,” and (2) a quantitative matrix based on the phenotypic measurements (Fig. 7A; see Methods and Supplemental Text S4). To build the genotype matrix, we defined “mutant” (0) as any allele affected by deletions, nonsense, or missense changes relative to the appropriate PacBio reference genome (i.e., that of the matching RAPD at the earliest available time point), whereas “wild-type” alleles were those matching the reference or with only silent substitutions. These simplifications assume that the reference used represents the ancestral wild-type allele and that any mutation affecting coding results in loss of function. We thus focused on motility and biofilm, because these two phenotypes were typically high for the isolates used as reference genomes and at the start of the longitudinal series (i.e., early isolates tended to have high swimming motility and produce robust biofilms) (see Methods and Supplemental File S14).

These correlations identified numerous genes associated with swimming motility and biofilm formation (Supplemental File S14). Within the top motility candidate genes are a number of known regulators of motility, including *dnaK* (ρ = 0.57, group_ 3108) (Fig. 7B), *cadA* (ρ = 0.46), *cheA* (ρ = 0.41), and *aer* (ρ = 0.41). In *Escherichia coli*, DnaK (also known as the heat shock protein 70) is a molecular chaperone required for flagellar synthesis and diverse stress responses (Shi et al. 1992). We identified other regulatory genes, including *rpoC* (ρ = 0.63) (Fig. 7D), *yecS* (ρ = 0.49), and *zraR* (ρ = 0.29), as well as hypothetical proteins with unknown function (ρ = 0.44) (Fig. 7C) that were also associated with motility variation.

Several candidate genes associated with the motility phenotype are also associated with biofilm formation, including *dnaK* (Fig. 7E), *papC*, *gcvA*, and *qseC* (ρ = 0.46, 0.38, 0.38, and 0.39, respectively). These four genes have been implicated in biofilm formation (Novais et al. 2013; Yang et al. 2014; Arita-Morioka et al. 2015). In three of the genes—*dnaK*, *papC*, and *gcvA*—gene loss alone was correlated with less motility and less biofilm formation (Supplemental Text S4; Supplemental Figs. S14, S15), suggesting they may regulate both phenotypes in *B. cenocepacia*.

## Discussion

### Resource for comparative genomics and genome assembly pipelines

Prior to our study, there were 26 publicly available *B. cenocepacia* genomes. Our data increase the number of genomes available to 241 across major epidemic lineages (including 11 reference quality assembles), which will serve as a valuable resource for future comparative genomic and transcriptomic analyses and expand our understanding of *B. cenocepacia* epidemics beyond the ET12 (RAPD02) lineage. Furthermore, the use of both Illumina short-read and PacBio long-read sequencing on a subset of strains provides a useful test data set for optimizing assembly algorithms, particularly for hybrid assembly of both data types together and for evaluating sources of assembly errors.

### Genome-wide association studies

In contrast to most comparative genomics studies, we directly evaluated clinically important phenotypes for the full strain collection. By using a simple Spearman rank correlation approach, we identified a number of candidate genes (*dnaK*, *papC*, *gcvA*, and *qseC*) that are highly associated with both the motility and biofilm phenotypes. Genes that regulate these clinically relevant traits could be promising targets for anti-virulence agents. Myricetin, a small molecule inhibitor of DnaK, prevents biofilm formation without inhibiting bacterial growth (Arita-Morioka et al. 2015). We expect the number of applications of this combined genotype–phenotype resource will be large; for example, more sophisticated machine learning analyses could consider how combinations of genes contribute to the observed phenotypes.

### Experimental evolution

Previous experimental evolution studies with *B. cenocepacia* strain HI2424 (subgroup B) found that lines evolved over 1000 generations to have increased biofilm formation had lost motility, suggesting an evolutionary trade-off between these two phenotypes (Traverse et al. 2013; Cooper 2014; Cooper et al. 2014). Consistent with this in vitro finding, subgroup B isolates from patient P09 showed an inverse correlation between the two phenotypes. In contrast, in subgroup A isolates, we observed a strong positive correlation between biofilm and motility. This raises the interesting possibility that the genetic architecture of these traits is quite different between the two subgroups, and we hypothesize that similar experiments in a distinct genetic background could reveal distinct phenotypic correlations in evolved lines.

### Clinical significance

Our phenotypic analyses showed extensive variation among *B. cenocepacia* isolates both within and among RAPD types and patient series. Strains from a given RAPD type were more similar phenotypically but still showed considerable variation, even among closely related strains. Within a subset of longitudinal series, we observed temporal trends toward decreasing motility, biofilm formation, acute virulence, and growth rate. These findings may be useful for clinical microbiologists in associating potential virulence traits with standard molecular diagnostic testing.

Previous genotyping methods suggested that chronic *B. cenocepacia* infections in CF patients result from the colonization of a few genetically “clonal” bacterial strains (Romling et al. 1994a,b). We now know there can be a large phenotypic and genotypic diversity within a single patient at any sampling point (Smith et al. 2006; Clark et al. 2015; Darch et al. 2015). For instance, a random sampling of 44 *P. aeruginosa* colonies from a single sputum sample of a clinically stable CF patient showed a wide range of phenotypes, from variations in protease and exotoxin productions to antibiotic susceptibility (Darch et al. 2015). Consistent with this, we observed broad phenotypic and genotypic variation for *B. cenocepacia* isolates obtained from the same patient at the same time point (Zlosnik et al. 2014; Clark et al. 2015). We thus emphasize the value of bacterial isolate collections that not only sample longitudinally but also sample populations at single time points, irrespective of observed variation of colony morphology.

In summary, we compiled a comprehensive genotype and phenotype resource of 215 *B. cenocepacia* genomes, along with an extensive phenotypic data set, that emphasizes the importance of both in understanding the role of different strains in disease progression in chronic infections. We expect this rich resource will provide for interrogating clinical isolates of *B. cenocepacia* both to address basic biological questions about how bacteria evolve within infections and to help characterize future outbreaks.

## Methods

### DNA extraction and genome sequencing

Genomic DNA was extracted from *B. cenocepacia* cultures using the Puregene Gentra archival bacterial DNA extraction kit (Qiagen). Multiplexed sequencing libraries were made using the Illumina Nextera XT DNA sample prep kit (Illumina). Sequencing was performed with single-end and paired-end reads at the UBC Sequencing Centre (University of British Columbia) to a minimum read depth of 60×. PacBio sequencing was performed using two SMRTcells per isolate with P4-C2 chemistry at the Clinical and Translational Research Institute Genomics Core Facility (Drexel University).

### Clinical isolate collection

Clinical data for all patients in this study were previously collected, as approved by the UBC Research Ethics Boards (H07-01396) (Zlosnik et al. 2011).

### Phenotypic analyses

#### In vitro growth assays in LB and SCFM

*B. cenocepacia* isolates were grown in one-half strength LB at 37°C overnight, washed with 10 mM MgSO_4_, and inoculated into either one-half LB or SCFM (Palmer et al. 2007) at OD_600_ = 0.1, in clear, flat-bottom 96-well microtiter plates.

#### Biofilm assay

We tested the ability of *B. cenocepacia* strains to form biofilm following a previously established protocol (O'Toole and Kolter 1998; Conway et al. 2002). Biofilm assays were conducted in 96-well polypropylene microtiter dishes containing SCFM (Palmer et al. 2007).

#### Mucoid phenotype classification

*B. cenocepacia* isolates were streaked to yeast extract mannitol media, and mucoidy phenotype was assessed following a previously established protocol (Zlosnik and Speert 2010; Zlosnik et al. 2011, 2015).

#### Motility assay

The swimming motility for each isolate was individually measured using the motility agar (0.3% LB agar plates) as previously described (Zlosnik et al. 2015).

#### Galleria mellonella *killing assay*

Fresh overnight cultures of *B. cenocepacia* isolates were grown in one-half strength LB, pelleted, and washed with 10 mM MgSO_4_. Each larva was injected with 10 μL of 10^6^ or 10^7^ cfu/mL (equivalent of 10^4^ or 10^5^ cfu, respectively) of bacteria plus 1.2 mg/mL of ampicillin to the hindmost left proleg using the BD ultra-fine II insulin syringe (Van Leene et al. 2007).

### Statistical analyses

All phenotypic data were analyzed in R (R Core Team 2016). For details, see Supplemental Methods.

### De novo genome assembly

We assembled *B. cenocepacia* genomes from Illumina reads using a custom assembly pipeline, which includes Trimmomatic-v0.30 (Bolger et al. 2014), COPE-v1.1.2 (Liu et al. 2012), ALLPATHS-LG (Butler et al. 2008), and Ray-v2.2.0 assembler (Boisvert et al. 2012).

For PacBio-sequenced isolates (Bcc001, Bcc022, Bcc023, Bcc030, Bcc116, Bcc129, Bcc137, Bcc173, Bcc174, Bcc179, Bcc201, and J2315), the PacBio reads were assembled with the HGAP assembler and the consensus sequence polishing by the Quiver algorithm (Chin et al. 2013) using the SMRT Analysis Suite (Pacific Biosciences) and circulated with Circlator (Hunt et al. 2015). Base modification analysis was performed with the SMRT Analysis Suite using standard mapping protocols. All assembled genomes were annotated with the rapid prokaryotic genome annotation pipeline, Prokka, v1.12 (Seemann 2014).

### Pan-genome analyses

Pan-genome analyses for all isolates with fewer than 500 contigs or for each RAPD genotype were performed using the rapid large-scale prokaryote pan-genome analysis pipeline, Roary, v3.4.2, from the Sanger Institute (https://github.com/sanger-pathogens/Roary; Page et al. 2015).

### Estimating population structure and phylogeny

Core-genome SNPs were extracted from the Roary core genome alignment for each RAPD genotype or for all 215 isolates using the SNP Sites program, v2.0.2, from the Sanger Institute (https://github.com/sanger-pathogens/snp_sites). These SNPs were used as input for maximum likelihood inference with RAxML, v8.2.0 (Stamatakis 2014). Phenotypic data were mapped onto the maximum-likelihood tree using the plotTree.R script (https://github.com/katholt/plotTree; Inouye et al. 2014).

### Variant calling and genome loss

Illumina reads were aligned to the NCBI or PacBio RAPD reference genome using the short-read aligner BWA, v0.7.9a (Li and Durbin 2009). Single-nucleotide variants were identified and filtered with the SAMtools toolbox, v1.1 (Li et al. 2009). We kept any variant locations with at least 80% of the mapped reads agreeing with the variant call and a root mean square mapping quality of ≥30.

### Identification of candidate genotype–phenotype associations

To identify variants that are associated with a phenotypic variation, we first identified 3055 gene families that were present in at least four RAPD genotypes as defined by Prokka (Seemann 2014) and Roary (Page et al. 2015). We excluded RAPD15 from the analyses due to small sample size (two isolates total). We generated a matrix of presence (one) versus absent (zero) genes, where we arbitrarily defined absent genes as any gene affected by small indels, nonsense and missense mutations, or deletions detected via our read coverage analysis described in the previous section. We performed Spearman's rank correlation between the phenotype and genotype matrices in order to find genes most strongly associated with each individual phenotype.

## Data access

The raw sequence data from this study have been submitted to the NCBI BioProject (http://www.ncbi.nlm.nih.gov/bioproject) under accession number PRJNA289138 and can be accessed from the Sequence Read Archive (SRA; https://www.ncbi.nlm.nih.gov/sra) with accession number SRP075474. This Whole Genome project has been submitted to the DDBJ/ENA/GenBank (https://www.ncbi.nlm.nih.gov/genbank/) under the accession numbers MUJN00000000–MUJS00000000, MUPR00000000–MUWY00000000, and CP019664–CP019678.

## Supplementary Material

Supplemental Material
