# Supplementary material for: Phenotypic diversity and genotypic flexibility of Burkholderia cenocepacia during long-term chronic infection of cystic fibrosis lungs
Source: Genome Res. 2017 Apr;27(4):650–62. doi: 10.1101/gr.213363.116 (PMC5378182; doi:10.1101/gr.213363.116)
Supplement: Supplemental Material [file supp_27_4_650__index.html]

Phenotypic diversity and genotypic flexibility of Burkholderia cenocepacia during long-term chronic infection of cystic fibrosis lungs — Phenotypic diversity and genotypic flexibility of Burkholderia cenocepacia during long-term chronic infection of cystic fibrosis lungs — Supplemental Material 

# Phenotypic diversity and genotypic flexibility of *Burkholderia cenocepacia* during long-term chronic infection of cystic fibrosis lungs

## Supplemental Material

- Supplemental\_Tables.pdf
- Supplemental\_FigureS1\_raw\_biofilm.csv
- Supplemental\_FigureS8\_Pair-wise\_ANI.csv
- Supplemental\_FigureS12\_recurrent\_gene\_loss.csv
- Supplemental\_FigureS6\_logistic\_growth\_SCFM.csv
- Supplemental\_FigureS10\_Roary\_gene\_presence\_absence\_original.csv
- Supplemental\_FigureS4\_raw\_moth\_10\_5.csv
- Supplemental\_FigureS2\_raw\_motility.csv
- Supplemental\_FigureS9\_Pair-wise\_tetranucleotide.csv
- Supplemental\_FigureS13\_recurrent\_gene\_gain.csv
- Supplemental\_FigureS7\_patient\_lung\_function\_all.csv
- Supplemental\_FigureS11\_Roary\_gene\_presence\_absence\_paralogs\_merged.csv
- Supplemental\_FigureS5\_logistic\_growth\_halfLB.csv
- Supplemental\_FigureS3\_raw\_moth\_10\_4.csv
- Supplemental\_FigureS14\_correlation\_gene\_loss\_and\_phenotype.csv
- Supplemental\_Methods\_Text\_Figures.docx
