# Supplementary material for: Phenotypic diversity and genotypic flexibility of Burkholderia cenocepacia during long-term chronic infection of cystic fibrosis lungs
Source: Genome Res. 2017 Apr;27(4):650–62. doi: 10.1101/gr.213363.116 (PMC5378182; doi:10.1101/gr.213363.116)
Supplement: Supplemental Material [file supp_gr.213363.116_Supplemental_Tables.pdf]

Table S1A. Linear regression of patient lung function over time (% FEV1, percent predicted forced expiratory volume in 1 sec).

Table S1B. Linear regression of patient lung function over time (%FVC, percent predicted forced vital capacity).

Table S1C. Linear regression of patient lung function over time (%FEF25-75, percent predicted forced expiratory flow at 25 – 75% of FVC).

Table S1D. Linear regression of swimming motility by longitudinal series of bacterial isolates over time. Swimming motility was measured as the diameter of the motility zone in mm (maximum of 90 mm).

Table S1E. Linear regression of biofilm formation by longitudinal series of bacterial isolates over time. Biofilm formation was measured by taking the absorbance value at 595 nm of crystal violet staining of 48-hr static bacterial cultures.

Table S1F. Linear regression of acute virulence (low dose) by longitudinal series of bacterial isolates over time. Low dose acute virulence (50 larvae, 10K cfu / 10µl injection) was assessed using a normalized rmeans value from Kaplan-Meier survival analysis over two days, where 0 indicates survival by all larvae, and 1 indicates no larvae survived after 1 day.

Table S1G. Linear regression of acute virulence (high dose) by longitudinal series of bacterial isolates over time. Low dose acute virulence (50 larvae, 100K cfu / 10µl injection) was assessed using a normalized rmeans value from Kaplan-Meier survival analysis over two days, where 0 indicates survival by all larvae, and 1 indicates no larvae survived after 1 day.

Table S1H. Logistic regression of mucoidy by longitudinal series of bacterial isolates over time. Mucoidy was measured qualitatively on yeast extract mannitol agar as 0 for non-mucoid and 1 for mucoid phenotypes. Only the six longitudinal series with variation in mucoidy are shown.

Table S1I. Linear regression of maximum yield in half-LB by longitudinal series of bacterial isolates over time. Maximum yield in half-LB was measured quantitatively using 96-well Tecan plate reader to monitor growth over 24 hours, with maximum yield modeled by logistic growth using growthcurve in R.

Table S1J.Linear regression of maximum growth rate in half-LB by longitudinal series of bacterial isolates over time. Maximum growth rate in half-LB was measured quantitatively using 96-well Tecan plate reader to monitor growth over 24 hours, with maximum growth rate modeled by logistic growth using growthcurve in R.

Table S1K. Linear regression of maximum yield in SCFM by longitudinal series of bacterial isolates over time. Maximum yield in SCFM was measured quantitatively using 96-well Tecan plate reader to monitor growth over 24 hours, with maximum yield modeled by logistic growth using growthcurve in R.

Table S1L. Linear regression of maximum growth rate in SCFM by longitudinal series of bacterial isolates over time. Maximum growth rate in SCFM was measured quantitatively using 96-well Tecan plate reader to monitor growth over 24 hours, with maximum yield modeled by logistic growth using growthcurve in R.

Table S1A. Linear regression of patient lung function over time (% FEV1, percent predicted forced expiratory volume in 1 sec).

**Patient**	**RAPD**	**p.adjust**	**Intercept**	**Change/Year**	**%Change/Year**	**R-squared**
P13	RAPD01	0.418	36.10	NA	NA	NA
**P16**	RAPD01	**0.001**	46.49	-2.91	-6.3%	0.39
**P04**	RAPD02	**<0.001**	45.39	-4.17	-9.2%	0.68
**P11**	RAPD02	**0.001**	28.57	-4.24	-14.8%	0.76
**P12**	RAPD02	**0.002**	81.00	-7.05	-8.7%	0.81
**P14**	RAPD02	**0.013**	74.46	-1.61	-2.2%	0.72
**P15**	RAPD02	**0.001**	44.27	-12.05	-27.2%	0.92
**P02**	RAPD04	**0.001**	35.28	-0.75	-2.1%	0.59
**P03**	RAPD04	**0.001**	88.75	-1.69	-1.9%	0.55
**P05**	RAPD04	**0.001**	25.69	-0.60	-2.3%	0.59
**P07**	RAPD04	**0.001**	74.61	-1.17	-1.6%	0.70
**P01**	RAPD06	**0.010**	71.99	-2.84	-3.9%	0.36
**P06**	RAPD06	**<0.001**	59.34	-5.48	-9.2%	0.90
P10	RAPD06	0.322	47.85	NA	NA	NA
P08	RAPD09	0.528	46.12	NA	NA	NA
P09	RAPD44.15	0.130	94.75	NA	NA	NA

Table S1B. Linear regression of patient lung function over time (%FVC, percent predicted forced vital capacity).

Patient	RAPD	p.adjust	Intercept	Change/Year	%Change/Year	R-squared
**P13**	RAPD01	**0.002**	79.87	-4.19	-5.2%	0.89
**P16**	RAPD01	**<0.001**	68.74	-5.01	-7.3%	0.58
**P04**	RAPD02	**0.002**	56.86	-4.12	-7.2%	0.55
**P11**	RAPD02	**0.037**	50.56	-4.00	-7.9%	0.38
**P12**	RAPD02	**0.006**	88.34	-6.61	-7.5%	0.75
**P14**	RAPD02	**0.029**	87.52	-1.82	-2.1%	0.62
**P15**	RAPD02	**0.007**	66.54	-13.57	-20.4%	0.79
**P02**	RAPD04	**0.004**	50.24	-0.58	-1.2%	0.45
**P03**	RAPD04	**0.031**	102.75	-0.89	-0.9%	0.24
**P05**	RAPD04	**0.001**	59.39	-0.99	-1.7%	0.69
**P07**	RAPD04	**0.001**	88.17	-1.29	-1.5%	0.65
**P01**	RAPD06	**<0.001**	105.60	-3.30	-3.1%	0.59
**P06**	RAPD06	**<0.001**	74.00	-6.07	-8.2%	0.95
**P10**	RAPD06	**0.031**	67.74	-1.57	-2.3%	0.32
P08	RAPD09	0.450	85.36	NA	NA	NA
**P09**	RAPD44.15	**0.015**	86.67	1.19	1.4%	0.41

Table S1C. Linear regression of patient lung function over time (%FEF25-75, percent predicted forced expiratory flow at 25 – 75% of FVC).

Patient	RAPD	p.adjust	Intercept	Change/Year	%Change/Year	R-squared
**P13**	RAPD01	**<0.001**	66.09	-7.04	-10.6%	0.98
**P16**	RAPD01	**0.019**	15.91	-0.99	-6.2%	0.25
**P04**	RAPD02	**0.040**	19.07	-1.79	-9.4%	0.30
**P11**	RAPD02	**<0.001**	12.33	-2.23	-18.1%	0.84
**P12**	RAPD02	**0.002**	70.25	-8.64	-12.3%	0.85
P14	RAPD02	0.111	34.93	NA	NA	NA
**P15**	RAPD02	**0.002**	15.19	-4.62	-30.4%	0.89
**P02**	RAPD04	**0.028**	17.82	-0.59	-3.3%	0.59
P03	RAPD04	0.212	40.88	NA	NA	NA
P05	RAPD04	0.134	7.18	NA	NA	NA
**P07**	RAPD04	**0.040**	45.47	-1.34	-2.9%	0.29
**P01**	RAPD06	**<0.001**	30.95	-1.64	-5.3%	0.74
**P06**	RAPD06	**<0.001**	32.03	-3.69	-11.5%	0.81
P10	RAPD06	0.586	19.31	NA	NA	NA
**P08**	RAPD09	**0.019**	11.71	0.54	4.6%	0.73
P09	RAPD44.15	0.586	112.66	NA	NA	NA

Table S1D. Linear regression of swimming motility by longitudinal series of bacterial isolates over time. Swimming motility was measured as the diameter of the motility zone in mm (maximum of 90 mm).

**Patient**	**RAPD**	**p.adjust**	**Intercept**	**Change/Year**	**%Change/Year**	**R-squared**
P13	RAPD01	0.833	36.30	NA	NA	NA
**P16**	RAPD01	**0.012**	36.29	-3.40	-9.4%	0.30
P04	RAPD02	0.833	16.09	NA	NA	NA
P11	RAPD02	0.379	9.79	NA	NA	NA
P12	RAPD02	0.258	77.77	NA	NA	NA
P14	RAPD02	0.833	19.73	NA	NA	NA
P15	RAPD02	0.984	20.47	NA	NA	NA
**P02**	RAPD04	**0.001**	74.96	-3.39	-4.5%	0.67
**P03**	RAPD04	**0.008**	75.63	-3.40	-4.5%	0.47
P05	RAPD04	0.833	6.49	NA	NA	NA
P07	RAPD04	0.833	17.11	NA	NA	NA
**P01**	RAPD06	**0.034**	89.43	-2.52	-2.8%	0.24
P06	RAPD06	0.675	72.08	NA	NA	NA
P10	RAPD06	0.111	85.54	NA	NA	NA
**P08**	RAPD09	**0.008**	92.61	-13.30	-14.4%	0.56
**P09**	RAPD44.15	**0.010**	93.67	-3.60	-3.8%	0.54

Table S1E. Linear regression of biofilm formation by longitudinal series of bacterial isolates over time. Biofilm formation was measured by taking the absorbance value at 595 nm of crystal violet staining of 48-hr static bacterial cultures.

Patient	RAPD	p.adjust	Intercept	Change/Year	%Change/Year	R-squared
P13	RAPD01	0.323	0.288	NA	NA	NA
**P16**	RAPD01	**<0.001**	0.364	-0.031	-8.53%	0.653
P04	RAPD02	0.890	0.082	NA	NA	NA
P11	RAPD02	0.348	0.124	NA	NA	NA
**P12**	RAPD02	**0.011**	0.078	0.032	41.89%	0.789
P14	RAPD02	0.890	0.140	NA	NA	NA
P15	RAPD02	0.163	0.167	NA	NA	NA
P02	RAPD04	0.111	0.252	NA	NA	NA
**P03**	RAPD04	**0.011**	0.234	-0.012	-5.27%	0.474
P05	RAPD04	0.890	0.047	NA	NA	NA
P07	RAPD04	0.383	0.033	NA	NA	NA
P01	RAPD06	0.890	0.356	NA	NA	NA
P06	RAPD06	0.020	0.437	-0.029	-6.69%	0.484
P10	RAPD06	0.540	0.348	NA	NA	NA
P08	RAPD09	0.368	0.095	NA	NA	NA
**P09**	RAPD44.15	**0.029**	0.035	0.002	5.89%	0.430

Table S1F. Linear regression of acute virulence (low dose) by longitudinal series of bacterial isolates over time. Low dose acute virulence (50 larvae, 10K cfu / 10µl injection) was assessed using a normalized rmeans value from Kaplan-Meier survival analysis over two days, where 0 indicates survival by all larvae, and 1 indicates no larvae survived after 1 day.

**Patient**	**RAPD**	**p.adjust**	**Intercept**	**Change/Year**	**%Change/Year**	**R-squared**
P13	RAPD01	0.638	0.066	NA	NA	NA
P16	RAPD01	0.547	0.307	NA	NA	NA
P04	RAPD02	0.377	0.025	NA	NA	NA
P11	RAPD02	0.140	-0.054	NA	NA	NA
P12	RAPD02	0.377	0.027	NA	NA	NA
P14	RAPD02	0.377	0.032	NA	NA	NA
P15	RAPD02	0.861	0.322	NA	NA	NA
P02	RAPD04	0.377	0.023	NA	NA	NA
P03	RAPD04	0.058	0.425	-0.017	-4.0%	0.317
P05	RAPD04	0.546	0.689	NA	NA	NA
P07	RAPD04	0.058	0.772	NA	NA	NA
P01	RAPD06	0.546	0.125	NA	NA	NA
P06	RAPD06	0.546	0.159	NA	NA	NA
P10	RAPD06	0.505	0.109	NA	NA	NA
P08	RAPD09	0.377	0.365	NA	NA	NA
**P09**	RAPD44.15	**0.001**	0.998	-0.034	-3.4%	0.758

Table S1G. Linear regression of acute virulence (high dose) by longitudinal series of bacterial isolates over time. Low dose acute virulence (50 larvae, 100K cfu / 10µl injection) was assessed using a normalized rmeans value from Kaplan-Meier survival analysis over two days, where 0 indicates survival by all larvae, and 1 indicates no larvae survived after 1 day.

Patient	RAPD	p.adjust	Intercept	Change/Year	%Change/Year	R-squared
P13	RAPD01	0.744	0.43	NA	NA	NA
P16	RAPD01	0.428	0.62	NA	NA	NA
P04	RAPD02	0.834	0.52	NA	NA	NA
P11	RAPD02	0.140	0.04	NA	NA	NA
P12	RAPD02	0.214	0.37	NA	NA	NA
P14	RAPD02	0.744	-0.02	NA	NA	NA
P15	RAPD02	0.744	0.76	NA	NA	NA
P02	RAPD04	0.744	0.36	NA	NA	NA
**P03**	RAPD04	**0.011**	0.96	-0.02	-2.2%	0.517
P05	RAPD04	0.834	0.89	NA	NA	NA
P07	RAPD04	0.140	0.98	NA	NA	NA
P01	RAPD06	0.428	0.38	NA	NA	NA
P06	RAPD06	0.939	0.58	NA	NA	NA
P10	RAPD06	0.313	0.51	NA	NA	NA
P08	RAPD09	0.387	0.71	NA	NA	NA
P09	RAPD44.15	0.114	1.01	-0.01	-0.7%	0.543

Table S1H. Logistic regression of mucoidy by longitudinal series of bacterial isolates over time. Mucoidy was measured qualitatively on yeast extract mannitol agar as 0 for non-mucoid and 1 for mucoid phenotypes. Only the six longitudinal series with variation in mucoidy are shown.

Patient	RAPD	p.value	Intercept
P16	RAPD01	0.16	-0.61
P07	RAPD04	0.49	-0.84
P01	RAPD06	0.20	0.80
P06	RAPD06	0.99	21.05
P10	RAPD06	0.38	11.06
P08	RAPD09	0.99	31.04

Table S1I. Linear regression of maximum yield in half-LB by longitudinal series of bacterial isolates over time. Maximum yield in half-LB was measured quantitatively using 96-well Tecan plate reader to monitor growth over 24 hours, with maximum yield modeled by logistic growth using growthcurve in R.

Patient	RAPD	p.adjust	Intercept	Change/Year	%Change/Year	R-squared
P13	RAPD01	0.840	0.43	NA	NA	NA
P16	RAPD01	0.840	0.40	NA	NA	NA
P04	RAPD02	0.240	0.62	NA	NA	NA
P11	RAPD02	0.880	0.33	NA	NA	NA
P12	RAPD02	0.284	0.45	NA	NA	NA
P14	RAPD02	0.549	0.32	NA	NA	NA
P15	RAPD02	0.840	0.34	NA	NA	NA
P02	RAPD04	0.898	0.45	NA	NA	NA
P03	RAPD04	0.284	0.42	NA	NA	NA
P05	RAPD04	0.840	0.40	NA	NA	NA
P07	RAPD04	0.840	0.45	NA	NA	NA
P01	RAPD06	0.840	0.37	NA	NA	NA
P06	RAPD06	0.840	0.44	NA	NA	NA
P10	RAPD06	0.840	0.41	NA	NA	NA
P08	RAPD09	0.789	0.47	NA	NA	NA
P09	RAPD44.15	0.240	0.52	NA	NA	NA

Table S1J.Linear regression of maximum growth rate in half-LB by longitudinal series of bacterial isolates over time. Maximum growth rate in half-LB was measured quantitatively using 96-well Tecan plate reader to monitor growth over 24 hours, with maximum growth rate modeled by logistic growth using growthcurve in R.

Patient	RAPD	p.adjust	Intercept	Change/Year	%Change/Year	R-squared
P13	RAPD01	0.987	1.82E-05	NA	NA	NA
P16	RAPD01	0.378	2.28E-05	NA	NA	NA
P04	RAPD02	0.907	7.76E-06	NA	NA	NA
P11	RAPD02	0.970	7.24E-06	NA	NA	NA
P12	RAPD02	0.941	1.31E-05	NA	NA	NA
P14	RAPD02	0.168	4.71E-06	NA	NA	NA
P15	RAPD02	0.907	1.34E-05	NA	NA	NA
**P02**	RAPD04	**0.027**	1.44E-05	2.86E-07	2%	0.37
**P03**	RAPD04	**0.030**	1.72E-05	-2.98E-07	-1.70%	0.33
P05	RAPD04	0.987	1.30E-05	NA	NA	NA
**P07**	RAPD04	**0.005**	3.05E-05	-1.17E-06	-3.80%	0.65
P01	RAPD06	0.168	1.75E-05	NA	NA	NA
P06	RAPD06	0.168	1.09E-05	NA	NA	NA
P10	RAPD06	0.415	1.44E-05	NA	NA	NA
**P08**	RAPD09	**0.027**	3.25E-05	-2.33E-06	-7.20%	0.43
**P09**	RAPD44.15	**0.009**	3.74E-05	-9.79E-07	-2.60%	0.60

Table S1K. Linear regression of maximum yield in SCFM by longitudinal series of bacterial isolates over time. Maximum yield in SCFM was measured quantitatively using 96-well Tecan plate reader to monitor growth over 24 hours, with maximum yield modeled by logistic growth using growthcurve in R.

Patient	RAPD	p.adjust	Intercept	Change/Year	Change/Year	R-squared
P13	RAPD01	0.875	0.47	NA	NA	NA
P16	RAPD01	0.408	0.29	NA	NA	NA
P04	RAPD02	0.875	0.28	NA	NA	NA
P11	RAPD02	0.764	0.29	NA	NA	NA
P12	RAPD02	0.764	0.47	NA	NA	NA
P14	RAPD02	0.884	0.18	NA	NA	NA
**P15**	RAPD02	**0.050**	0.28	-0.03	NA	0.78
P02	RAPD04	0.764	0.43	NA	NA	NA
**P03**	RAPD04	**0.004**	0.65	-0.02	-3.30%	0.58
P05	RAPD04	0.875	0.40	NA	NA	NA
**P07**	RAPD04	**0.028**	0.50	0.00	-0.90%	0.45
P01	RAPD06	0.764	0.37	NA	NA	NA
**P06**	RAPD06	**0.010**	0.32	0.02	5.90%	0.67
P10	RAPD06	0.100	0.45	NA	NA	NA
P08	RAPD09	0.875	0.47	NA	NA	NA
P09	RAPD44.15	0.875	0.46	NA	NA	NA

Table S1L. Linear regression of maximum growth rate in SCFM by longitudinal series of bacterial isolates over time. Maximum growth rate in SCFM was measured quantitatively using 96-well Tecan plate reader to monitor growth over 24 hours, with maximum yield modeled by logistic growth using growthcurve in R.

**Patient**	**RAPD**	**p.adjust**	**Intercept**	**Change/Year**	**Change/Year**	**R-squared**
P13	RAPD01	0.986	2.18E-05	NA	NA	NA
P16	RAPD01	0.590	9.91E-06	NA	NA	NA
P04	RAPD02	0.226	1.03E-05	NA	NA	NA
P11	RAPD02	0.590	1.10E-05	NA	NA	NA
P12	RAPD02	0.207	7.35E-06	NA	NA	NA
P14	RAPD02	0.986	2.45E-06	NA	NA	NA
P15	RAPD02	0.921	1.13E-05	NA	NA	NA
P02	RAPD04	0.851	2.25E-05	NA	NA	NA
**P03**	RAPD04	**0.003**	3.78E-05	0.00	-3.90%	0.55
P05	RAPD04	0.207	1.21E-05	NA	NA	NA
**P07**	RAPD04	**<0.001**	3.13E-05	0.00	-4.30%	0.81
P01	RAPD06	0.637	1.88E-05	NA	NA	NA
P06	RAPD06	0.590	1.16E-05	NA	NA	NA
P10	RAPD06	0.921	1.86E-05	NA	NA	NA
P08	RAPD09	0.959	2.04E-05	NA	NA	NA
P09	RAPD44.15	0.590	3.36E-05	NA	NA	NA

Table S2. The in vitro phenotypes of *B. cenocepacia* isolates from patients P01 to P16 and the date of isolation. Patient lung functions were assessed by the percent predicted forced expiratory volume in one second (%FEV_1), percent forced vital capacity (%FVC) and percent forced expiratory flow at 25 – 75% of FVC (%$FEV_{25\text{-}75}$). The closest dates for which we had patient lung functions and *B. cenocepacia* isolates were included in the analysis. *In vitro* phenotype data for swimming motility (measured as the diameter of the motility zone in mm), biofilm formation (average absorbance value of crystal violet staining), virulence (rmeans value in the Kaplan-Meier survival analysis for 10^4 or 10^5 cfu/10µl infection doses) and mucoidy (as assessed on yeast mannitol agar) are shown. Additionally, the *in vitro* maximum growth and growth rate in 96-well microtitre plates containing SCFM or half-strength LB are shown.

Column	Description
BioProject id	NCBI BioProject
BioSample Accession	NCBI BioSample associated with each isolate
Strain	Strain ID assigned by the Canadian Burkholderia cepacia Complex Research and Referral Repository
Isolate_ID	Sample ID for each isolate used in the laboratory
Patient_ID	Patient ID for each patient used in the laboratory
Isolate_Date	Isolation date for each isolate
RAPD	RAPD genotyping as definited by the clinical microbiologist
swimming	Swimming motility zone in mm using the motility agar (0.3% LB agar plates)
biofilm_avg	Average crystal violet staining measured by absorbance wavelength of595nm
mucoidy.norm	Mucoid phenotype assessed on YEM agar plates, 1 = mucoid, 0 = nonmucoid
virulence4.norm	G. mellonella Killing assay to assess virulence, each larva was injected with 10^4 cfu and rmeans value indicated
virulence5.norm	G. mellonella Killing assay to assess virulence, each larva was injected with 10^5 cfu and rmeans value indicated
FEV1_normal	patient lung function from clinical metadata for %FEV1 (percent predicted forced expiratory volume in 1 sec)
FVC_normal	patient lung function from clinical metadata for %FVC (percent predicted forced vital capacity)
FEV_25_75	patient lung function from clinical metadata for %FEF25-75 (percent predicted forced expiratory flow at 25 – 75% of FVC)
Avg_MaxGrowth_SCFM	Average maximum growth/yield estimated by logistic growth curve for replicates grown in synthetic cystic fibrosis media
Avg_MaxRate_SCFM	Average maximum growth rate estimated by logistic growth curve for replicates grown in synthetic cystic fibrosis media
Avg_MaxGrowth_LB	Average maximum growth/yield estimated by logistic growth curve for replicates grown in 1/2 LB
Avg_MaxRate_LB	Average maximum growth rate estimated by logistic growth curve for replicates grown in 1/2 LB

Table S2. The in vitro phenotypes of *B. cenocepacia* isolates from patients P01 to P16 and the date of isolation. Patient lung functions were assessed by the percent predicted forced expiratory volume in one second (%FEV_1), percent forced vital capacity (%FVC) and percent forced expiratory flow at 25 – 75% of FVC (%FEV_{25-75}). The closest dates for which we had patient lung functions and *B. cenocepacia* isolates were included in the analysis. *In vitro* phenotype data for swimming motility (measured as the diameter of the motility zone in mm), biofilm formation (average absorbance value of crystal violet staining), virulence (rmeans value in the Kaplan-Meier survival analysis for 10^4 or 10^5 cfu/10µl infection doses) and mucoidy (as assessed on yeast mannitol agar) are shown. Additionally, the *in vitro* maximum growth and growth rate in 96-well microtitre plates containing SCFM or half-strength LB are shown.

BioProject id	BioSample Accession	Strain	Isolate ID	Patient ID	Isolate Date	Isolate Sites	RAPD	swimming	biofilm_avg	mucoidy.norm	virulence4.norm	virulence5.norm	FEV1_normal	FVC_normal	FEV_25_75	Avg_MaxGrowth_SCFM	Avg_MaxRate_SCFM	Avg_MaxGrowth_LB	Avg_MaxRate_LB
289138	SAMN05001798	VC2307	Bcc001	P01	1987-04-04	Sputum	RAPD06	90.00	0.34932222	1	0.011173185	0.013513514	NA	118	34	0.385265662	2.20E-05	0.413958307	2.17E-05
289138	SAMN05001799	VC2387	Bcc002	P01	1987-05-24	Sputum	RAPD06	90.00	0.06603333	0	0.261111111	0.617977528	NA	106	33	NA	NA	0.174314437	5.45E-06
289138	SAMN05001800	VC2386	Bcc003	P01	1987-05-24	Sputum	RAPD06	90.00	0.27218889	1	0.413774105	0.982456141	NA	106	33	0.372172279	1.85E-05	0.397861454	1.79E-05
289138	SAMN05001801	VC2448	Bcc004	P01	1987-07-19	Sputum	RAPD06	90.00	0.43328889	1	0.005617978	0.015300362	NA	105	24	0.420005601	2.79E-05	0.40451499	2.26E-05
289138	SAMN05001802	VC2449	Bcc005	P01	1987-07-19	Sputum	RAPD06	75.50	0.34064444	0	0.015461442	0.019822716	NA	105	24	0.393954282	1.69E-05	0.418425321	2.15E-05
289138	SAMN05001803	VC4462	Bcc006	P01	1991-10-29	Sputum	RAPD06	90.00	0.51846667	1	0.043603314	0.4375	58	78	30	0.280861086	6.27E-06	0.353791247	1.37E-05
289138	SAMN05001804	VC5018	Bcc007	P01	1992-12-09	Sputum	RAPD06	79.00	0.48928889	1	0.056603774	0.491739069	50	75	20	0.379632203	1.45E-05	0.383129053	1.27E-05
289138	SAMN05001805	VC5280	Bcc008	P01	1993-06-08	Sputum	RAPD06	75.25	0.52395556	1	0.0874375	0.540540541	44	70	18	0.395329731	1.55E-05	0.393490689	1.22E-05
289138	SAMN05001806	VC5279	Bcc009	P01	1993-06-08	Sputum	RAPD06	90.00	0.42981111	0	0.15934066	0.678571429	44	70	18	0.389830638	1.77E-05	0.361179056	1.43E-05
289138	SAMN05001807	VC6133	Bcc010	P01	1994-05-16	Sputum	RAPD06	66.50	0.38326667	0	0.036363637	0.48757764	60	91	25	0.325985714	7.03E-06	0.389267393	1.29E-05
289138	SAMN05001808	VC6581	Bcc011	P01	1994-09-30	Sputum	RAPD06	74.00	0.40727778	0	0.081698113	0.550458716	48	70	19	0.309484422	9.80E-06	0.368789654	1.48E-05
289138	SAMN05001809	VC6579	Bcc012	P01	1994-09-30	Sputum	RAPD06	75.00	0.38592222	0	0.112262038	0.553398059	48	70	19	0.308045303	9.08E-06	0.384446348	1.25E-05
289138	SAMN05001810	VC6905	Bcc013	P01	1995-03-07	Sputum	RAPD06	8.00	0.42787778	0	0	0.235358132	59	92	19	0.490102267	2.15E-05	0.515222459	1.59E-05
289138	SAMN05001811	VC6906	Bcc014	P01	1995-03-07	Sputum	RAPD06	90.00	0.4427	1	0.821917808	1	59	92	19	0.432439789	1.80E-05	0.459578004	1.55E-05
289138	SAMN05001812	VC7485	Bcc015	P01	1996-02-09	Sputum	RAPD06	57.50	0.42098889	0	0.03719509	0.508474577	56	86	20	0.470193631	1.27E-05	0.35903817	1.34E-05
289138	SAMN05001813	VC7488	Bcc016	P01	1996-02-09	Sputum	RAPD06	71.00	0.16445556	1	1	1	56	86	20	0.555257768	3.09E-05	0.437359178	1.59E-05
289138	SAMN05001814	VC7489	Bcc017	P01	1996-02-09	Sputum	RAPD06	90.00	0.4054	1	0.910447761	1	56	86	20	0.486493521	2.53E-05	0.504542582	2.27E-05
289138	SAMN05001815	VC7945	Bcc018	P01	1996-11-08	Sputum	RAPD06	59.00	0.43145556	0	0.02827239	0.483635301	41	83	10	0.352987636	1.09E-05	0.311833337	8.39E-06
289138	SAMN05001816	VC8379	Bcc019	P01	1997-08-21	Sputum	RAPD06	68.50	0.33862222	1	0.983606558	1	42	85	11	0.359003182	1.24E-05	0.412747468	1.30E-05
289138	SAMN05001817	VC9041	Bcc020	P01	1998-07-02	Sputum	RAPD06	66.00	0.20137778	0	0.01868323	0.004201681	36	58	12	0.428040492	1.55E-05	0.364416006	9.73E-06
289138	SAMN05001818	VC9040	Bcc021	P01	1998-07-02	Sputum	RAPD06	59.00	0.37123333	0	0.050458716	0.530973452	36	58	12	0.331721179	1.47E-05	0.278529158	6.30E-06
289138	SAMN05001819	VC9789	Bcc022	P01	1999-08-13	Sputum	RAPD06	53.75	0.23204444	0	0	0.426537299	28	62	7	0.438202762	1.91E-05	0.425278366	1.71E-05
289138	SAMN05001820	VC4558	Bcc023	P02	1992-01-17	Sputum	RAPD04	74.00	0.25613333	0	0.072994841	0.566037736	31	44	NA	0.492749816	2.57E-05	0.384932559	1.46E-05
289138	SAMN05001821	VC3929	Bcc024	P02	1990-10-11	Sputum	RAPD04	75.50	0.23927778	0	0.044364374	0.523333334	39	51	NA	0.417346056	2.50E-05	0.402370904	1.49E-05
289138	SAMN05001822	VC5732	Bcc025	P02	1994-01-09	Sputum	RAPD04	68.75	0.26333333	0	0.059633028	0.625	31	49	NA	0.445811836	2.06E-05	0.410416923	1.19E-05
289138	SAMN05001823	VC5731	Bcc026	P02	1994-01-13	Sputum	RAPD04	55.25	0.107	0	0.004201681	0.318782927	31	49	NA	0.484274848	2.95E-05	0.468460643	1.68E-05
289138	SAMN05001824	VC6904	Bcc027	P02	1995-03-07	Sputum	RAPD04	50.00	0.36166667	0	0.023190159	0.467799964	26	43	NA	0.475523915	2.02E-05	0.405995017	1.38E-05
289138	SAMN05001825	VC6903	Bcc028	P02	1995-03-07	Sputum	RAPD04	72.50	0.30233333	0	0.050458716	0.431497175	26	43	NA	0.399888355	1.73E-05	0.469456299	1.62E-05
289138	SAMN05001826	VC7603	Bcc029	P02	1996-04-04	Sputum	RAPD04	25.00	0.05433333	0	0.009733893	0.055555556	38	49	18	0.39269794	2.01E-05	0.423992093	1.43E-05
289138	SAMN05001827	VC7604	Bcc030	P02	1996-04-04	Sputum	RAPD04	59.00	0.285	0	0.005617978	0.040433152	38	49	18	0.427567073	2.18E-05	0.491648165	1.86E-05
289138	SAMN05001828	VC8057	Bcc031	P02	1997-01-31	Sputum	RAPD04	59.50	0.27033333	0	0.00862069	0.138297873	28	46	9	0.461494222	2.17E-05	0.655673482	1.47E-05
289138	SAMN05001829	VC8556	Bcc032	P02	1997-12-12	Sputum	RAPD04	30.50	0.10933333	0	0.009899078	0.205157132	36	58	NA	0.462388713	2.84E-05	0.491238499	1.94E-05
289138	SAMN05001830	VC9612	Bcc033	P02	1999-05-07	Sputum	RAPD04	69.50	0.23333333	0	0.0874375	0.597938145	28	49	10	0.368554296	1.20E-05	0.445606243	1.41E-05
289138	SAMN05001831	VC10178	Bcc034	P02	2000-05-12	Sputum	RAPD04	74.50	0.05333333	0	0.037871341	0.459316395	29	43	NA	0.371131546	1.49E-05	0.493075585	2.20E-05
289138	SAMN05001832	VC13300	Bcc035	P02	2006-03-08	Sputum	RAPD04	14.00	0.06966667	0	0.012820513	0.376985112	23	38	NA	0.46021726	2.51E-05	0.439070777	1.68E-05
289138	SAMN05001833	VC14507	Bcc036	P02	2008-10-20	Sputum	RAPD04	4.00	0.133	0	0.03719509	0.216063349	21	39	7	0.463388794	2.43E-05	0.441178648	1.89E-05
289138	SAMN05001834	VC14748	Bcc037	P02	2009-04-17	Sputum	RAPD04	11.50	0.227	0	0.029872818	0.538461539	21	39	7	0.437637009	1.38E-05	0.442347697	2.15E-05
289138	SAMN05001835	VC14815	Bcc038	P02	2009-06-20	Sputum	RAPD04	13.00	0.02433333	0	0.070240987	0.591836735	21	39	7	0.450775231	1.93E-05	0.429190037	1.78E-05
289138	SAMN05001836	VC15049	Bcc039	P02	2009-12-10	Sputum	RAPD04	12.50	0.12566667	0	0.188910591	0.591836735	21	39	7	0.530038207	2.72E-05	0.460367852	2.06E-05
289138	SAMN05001837	VC3868	Bcc040	P03	1990-08-24	Throat	RAPD04	70.00	0.20666667	0	0.088776882	0.707317073	82	90	NA	0.603328299	3.19E-05	0.427861287	1.43E-05
289138	SAMN05001838	VC4480	Bcc041	P03	1991-11-07	Throat	RAPD04	69.50	0.228	0	0.345758522	0.882352941	93	103	NA	0.597756628	3.32E-05	0.403687985	1.34E-05
289138	SAMN05001839	VC5300	Bcc042	P03	1993-06-24	Sputum	RAPD04	58.00	0.249	0	0.552091879	1	87	102	NA	0.589855774	3.61E-05	0.514175034	2.24E-05
289138	SAMN05001840	VC8945	Bcc043	P03	1998-06-04	Sputum	RAPD04	86.50	0.034	0	0.37042683	0.808219178	79	106	36	0.63400787	4.27E-05	0.382982149	1.54E-05
289138	SAMN05001841	VC8946	Bcc044	P03	1998-06-04	Sputum	RAPD04	71.50	0.30633333	0	0.457338454	0.893939394	79	106	36	0.656914607	3.84E-05	0.467024828	1.63E-05
289138	SAMN05001842	VC8947	Bcc045	P03	1998-06-04	Sputum	RAPD04	67.50	0.25233333	0	0.32532833	0.821917808	79	106	36	0.623565284	3.34E-05	0.418397492	1.46E-05
289138	SAMN05001843	VC9670	Bcc046	P03	1999-06-04	Sputum	RAPD04	15.50	0.05733333	0	0.475909704	0.893939394	68	97	27	0.321967799	1.43E-05	0.454772528	1.35E-05
289138	SAMN05001844	VC10287	Bcc047	P03	2000-07-21	Sputum	RAPD04	15.50	0.034	0	0.398503033	0.95	93	104	57	0.415585433	1.87E-05	0.401818423	1.39E-05
289138	SAMN05001845	VC10878	Bcc048	P03	2001-08-10	Sputum	RAPD04	17.50	0.02066667	0	0.336123077	0.935483871	68	90	30	0.284656186	1.60E-05	0.418261062	1.36E-05
289138	SAMN05001846	VC11578	Bcc049	P03	2002-10-25	Sputum	RAPD04	16.50	0.00833333	0	0.062694704	0.618595826	55	76	13	0.24746792	7.79E-06	0.482757603	1.75E-05
289138	SAMN05001847	VC11575	Bcc050	P03	2002-10-25	Sputum	RAPD04	79.00	0.15133333	0	0.042803283	0.586996337	55	76	13	0.486013861	2.70E-05	0.438226468	1.23E-05
289138	SAMN05001848	VC12599	Bcc051	P03	2004-11-01	Sputum	RAPD04	21.50	0.01033333	0	0.177131984	0.634408602	60	93	NA	0.294813636	1.27E-05	0.509488813	1.42E-05
289138	SAMN05001849	VC13450	Bcc052	P03	2006-07-10	Sputum	RAPD04	13.00	0.008	0	0.096723357	0.527884399	53	83	22	0.176231629	6.86E-06	0.442896709	1.03E-05
289138	SAMN05001850	VC14013	Bcc053	P03	2007-10-22	Sputum	RAPD04	17.50	0.00633333	0	0.049399657	0.552015929	52	81	21	0.261138331	1.07E-05	0.472505622	1.06E-05
289138	SAMN05001851	VC14543	Bcc054	P03	2008-11-13	Sputum	RAPD04	4.00	0.07	0	0.103163494	0.632894737	63	89	27	0.2566486	1.19E-05	0.587737924	1.02E-05
289138	SAMN05001852	VC15235	Bcc055	P03	2010-05-10	Sputum	RAPD04	24.50	0.00833333	0	0.10721121	0.493380406	63	89	27	0.341864493	1.56E-05	0.499037588	1.34E-05
289138	SAMN05001853	VC15566	Bcc056	P03	2011-04-18	Sputum	RAPD04	2.00	0.01533333	0	0.051716299	0.489032997	63	89	27	0.265180661	8.04E-06	0.455647047	1.02E-05
289138	SAMN05001854	VC4927	Bcc057	P04	1992-10-29	Sputum	RAPD02	7.50	0.01166667	0	0.15970516	0.656875266	42	51	20	0.351555145	1.51E-05	NA	NA
289138	SAMN05001855	VC5748	Bcc058	P04	1994-01-21	Sputum	RAPD02	18.00	0.04733333	0	0.150666667	0.689655173	44	56	NA	0.196754668	2.31E-06	0.649570827	6.00E-06
289138	SAMN05001856	VC6533	Bcc059	P04	1994-09-09	Sputum	RAPD02	25.50	0.20233333	0	0.110810811	0.525632564	38	50	NA	0.254920267	4.22E-06	0.459563106	6.82E-06

289138	SAMN05001857	VC7233	Bcc060	P04	1995-07-20	Sputum	RAPD02	12.50	0.221	0	0.041697692	0.532710281	32	48	10	0.32911131	9.01E-06	0.397577667	1.25E-05
289138	SAMN05001858	VC7232	Bcc061	P04	1995-07-20	Sputum	RAPD02	15.00	0.08	0	0.09469534	0.525116083	32	48	10	0.271895781	9.70E-06	0.414966173	1.64E-05
289138	SAMN05001859	VC7348	Bcc062	P04	1995-09-29	Sputum	RAPD02	4.50	0.07866667	0	0.012195122	0.016192283	35	46	14	0.311708643	6.67E-06	0.417570052	1.13E-05
289138	SAMN05001860	VC7579	Bcc063	P04	1996-03-29	Sputum	RAPD02	40.00	0.27633333	0	0.075076985	0.488439955	32	42	15	0.366370144	2.85E-06	0.376198605	7.20E-06
289138	SAMN05001861	VC7580	Bcc064	P04	1996-03-29	Sputum	RAPD02	14.00	0.02833333	0	0.102667298	0.519607843	32	42	15	0.274616135	6.39E-06	0.393570375	9.52E-06
289138	SAMN05001862	VC7692	Bcc065	P04	1996-05-24	Sputum	RAPD02	4.00	0.161	0	0.348224196	0.473267327	34	46	15	0.261190635	4.52E-06	0.423335	1.18E-05
289138	SAMN05001863	VC7691	Bcc066	P04	1996-05-24	Sputum	RAPD02	17.75	0.138	0	0.337257618	0.580645162	34	46	15	0.258451592	3.66E-06	0.418067404	8.30E-06
289138	SAMN05001864	VC7690	Bcc067	P04	1996-05-24	Sputum	RAPD02	3.50	0.02166667	0	0.019011114	0.346311476	34	46	15	0.183637898	3.90E-06	0.379534219	4.85E-06
289138	SAMN05001865	VC7689	Bcc068	P04	1996-05-24	Sputum	RAPD02	14.00	0.079	0	0.051973104	0.563829787	34	46	15	0.313908839	2.32E-06	0.39372529	6.98E-06
289138	SAMN05001866	VC7779	Bcc069	P04	1996-07-18	Sputum	RAPD02	4.00	0.027	0	0.076485911	0.510416667	26	35	12	0.291537917	9.82E-06	0.399455591	1.43E-05
289138	SAMN05001867	VC7780	Bcc070	P04	1996-07-18	Sputum	RAPD02	20.00	0.23766667	0	0.248504984	0.666666667	26	35	12	0.580488312	3.69E-06	0.590824386	4.95E-06
289138	SAMN05001868	VC7985	Bcc071	P04	1996-12-08	Sputum	RAPD02	8.00	0.035	0	0.523684703	0.983606558	25	37	9	0.282471906	6.70E-06	0.404679093	1.45E-05
289138	SAMN05001869	VC7986	Bcc072	P04	1996-12-08	Sputum	RAPD02	6.00	0.014	0	0.53931624	0.904761905	25	37	9	0.269789092	4.58E-06	0.362805838	6.94E-06
289138	SAMN05001870	VC6916	Bcc073	P05	1995-03-10	Sputum	RAPD04	22.25	0.044	0	0.893939394	1	28	56	NA	0.316345604	7.96E-06	0.361775426	1.48E-05
289138	SAMN05001871	VC6917	Bcc074	P05	1995-03-10	Sputum	RAPD04	5.00	0.02733333	0	0.753246753	0.967213115	28	56	NA	0.381004142	1.14E-05	0.444116916	1.10E-05
289138	SAMN05001872	VC7378	Bcc075	P05	1995-10-20	Sputum	RAPD04	5.00	0.05833333	0	0.932795699	0.949152543	27	57	8	0.428279887	1.60E-05	0.448238323	1.76E-05
289138	SAMN05001873	VC7379	Bcc076	P05	1995-10-20	Sputum	RAPD04	4.00	0.05533333	0	0.880597015	0.983606558	27	57	8	0.435820391	1.47E-05	0.449969802	1.49E-05
289138	SAMN05001874	VC7884	Bcc077	P05	1996-10-04	Sputum	RAPD04	5.00	0.078	0	0.241451428	0.737854251	18	57	6	0.359581892	9.01E-06	0.368809242	1.31E-05
289138	SAMN05001875	VC8222	Bcc078	P05	1997-05-08	Sputum	RAPD04	2.00	0.02233333	0	0.79342723	1	23	62	7	NA	NA	0.351410572	8.96E-06
289138	SAMN05001876	VC9970	Bcc079	P05	1999-11-25	Sputum	RAPD04	6.00	0.03966667	0	0.072184406	0.500649773	19	57	6	0.335139804	1.24E-05	0.367162892	1.18E-05
289138	SAMN05001877	VC11482	Bcc080	P05	2002-08-16	Sputum	RAPD04	2.50	0.04366667	0	0.572815534	0.90625	25	57	6	0.40671626	8.69E-06	0.38152297	1.14E-05
289138	SAMN05001878	VC12229	Bcc081	P05	2004-01-23	Sputum	RAPD04	2.00	0.05066667	0	0.442003793	0.769230769	20	59	6	0.72827274	8.09E-06	0.37221693	9.62E-06
289138	SAMN05001879	VC13395	Bcc082	P05	2006-05-31	Sputum	RAPD04	3.00	0.05333333	0	0.789473684	1	21	49	8	0.36267197	1.06E-05	0.416975709	1.44E-05
289138	SAMN05001880	VC14165	Bcc083	P05	2008-01-28	Sputum	RAPD04	18.50	0.083	0	0.609664352	1	17	43	6	0.363342027	8.69E-06	0.382294514	1.23E-05
289138	SAMN05001881	VC14636	Bcc084	P05	2009-01-29	Sputum	RAPD04	20.75	0.02233333	0	0.595959596	1	17	43	6	0.304776819	4.38E-06	0.426652248	1.29E-05
289138	SAMN05001882	VC14637	Bcc085	P05	2009-01-29	Sputum	RAPD04	2.50	0.023	0	0.572815534	0.833333334	17	43	6	0.304781189	3.36E-06	0.446045942	1.22E-05
289138	SAMN05001883	VC15221	Bcc086	P05	2010-04-29	Sputum	RAPD04	4.00	0.06533333	0	0.597207304	1	17	43	6	0.378010526	1.11E-05	0.437483223	1.63E-05
289138	SAMN05001884	VC6072	Bcc087	P06	1994-04-13	Sputum	RAPD06	40.25	0.49733333	NA	0.248232522	0.65934066	62	73	39	0.345632348	9.22E-06	0.402756795	1.18E-05
289138	SAMN05001885	VC6666	Bcc088	P06	1994-11-15	Sputum	RAPD06	82.75	0.406	NA	0.059633028	0.572815534	53	68	26	0.34969598	9.51E-06	0.424749674	1.86E-05
289138	SAMN05001886	VC6890	Bcc089	P06	1995-02-24	Sputum	RAPD06	90.00	0.245	0	0.027776357	0.195684524	60	66	37	0.366200331	2.06E-05	0.352959236	1.34E-05
289138	SAMN05001887	VC7559	Bcc091	P06	1996-03-16	Sputum	RAPD06	39.50	0.454	NA	0.210843374	0.588235294	52	61	NA	NA	NA	0.687571103	8.00E-06
289138	SAMN05001888	VC7949	Bcc092	P06	1996-11-15	Sputum	RAPD06	74.75	0.388	NA	0.106060606	0.560747664	42	62	15	0.323438115	1.01E-05	0.357579464	6.07E-06
289138	SAMN05001889	VC8381	Bcc093	P06	1997-08-23	Sputum	RAPD06	90.00	0.349	0	0.271589724	0.681818182	45	64	16	0.333576065	8.54E-06	0.453998216	1.53E-05
289138	SAMN05001890	VC9478	Bcc094	P06	1999-02-08	Sputum	RAPD06	90.00	0.261	0	0.305129262	0.824324325	20	42	5	0.422277589	1.62E-05	0.514651198	1.95E-05
289138	SAMN05001891	VC10526	Bcc095	P06	2000-12-20	Sputum	RAPD06	54.25	0.334	NA	0.327330395	0.779220779	22	33	9	0.459380846	1.86E-05	0.528876467	1.95E-05
289138	SAMN05001892	VC10925	Bcc096	P06	2001-09-14	Sputum	RAPD06	52.50	0.33766667	NA	0	0.02468157	21	32	6	0.387211215	1.12E-05	0.46377077	9.40E-06
289138	SAMN05001893	VC10926	Bcc097	P06	2001-09-14	Sputum	RAPD06	65.00	0.2678	NA	0.200926436	0.55882353	21	32	6	0.503220928	1.84E-05	0.449277019	2.22E-05
289138	SAMN05001894	VC11461	Bcc098	P06	2002-08-02	Sputum	RAPD06	58.00	0.15742222	0	0.306506625	0.581632653	16	20	3	0.527507179	1.07E-05	0.452034364	1.99E-05
289138	SAMN05001895	VC11675	Bcc099	P06	2002-12-29	Sputum	RAPD06	51.25	0.02824444	0	0.127037618	0.467464115	16	20	3	0.493373414	1.90E-05	0.465394411	2.54E-05
289138	SAMN05001896	VC10018	Bcc100	P06	2000-01-03	Sputum	RAPD06	64.50	0.24704444	1	0.490802676	0.951612903	21	34	NA	NA	NA	0.361144508	1.01E-05
289138	SAMN05001897	VC6553	Bcc101	P07	1994-09-16	Sputum	RAPD04	5.00	0.03028889	0	0.614450128	0.966666667	69	86	38	0.527790373	3.11E-05	0.495723226	3.34E-05
289138	SAMN05001898	VC6639	Bcc102	P07	1994-11-01	Sputum	RAPD04	5.00	0.03104444	0	0.635062612	0.930555556	80	94	50	0.522159925	3.03E-05	0.501300905	3.18E-05
289138	SAMN05001899	VC7292	Bcc103	P07	1995-08-28	Sputum	RAPD04	6.00	0.03394445	0	0.935483871	1	70	87	38	0.478753725	2.74E-05	0.385104737	2.38E-05
289138	SAMN05001900	VC7530	Bcc104	P07	1996-03-05	Sputum	RAPD04	26.00	0.04164445	0	0.865671642	1	80	90	53	0.48198377	3.10E-05	0.432816301	3.01E-05
289138	SAMN05001901	VC8229	Bcc105	P07	1997-03-09	Sputum	RAPD04	12.50	0.03655556	1	0.842857143	0.983606558	70	80	46	0.491621945	3.26E-05	0.426928117	2.78E-05
289138	SAMN05001902	VC9137	Bcc106	P07	1998-09-11	Sputum	RAPD04	5.00	0.02782222	0	0.779220779	0.951612903	74	88	45	0.527058156	2.40E-05	0.450411386	1.88E-05
289138	SAMN05001903	VC9855	Bcc107	P07	1999-09-30	Sputum	RAPD04	90.00	0.04908889	0	0.724071208	1	69	81	40	0.458880026	2.96E-05	0.512850868	3.50E-05
289138	SAMN05001904	VC10394	Bcc108	P07	2000-10-05	Sputum	RAPD04	5.00	0.02088889	0	0.557692308	0.867647059	61	76	31	0.45648009	2.45E-05	0.423503525	2.35E-05
289138	SAMN05001905	VC11261	Bcc109	P07	2002-03-21	Sputum	RAPD04	5.50	0.01027778	1	0.471654004	0.722891567	63	71	37	0.403551356	1.46E-05	0.432809333	2.36E-05
289138	SAMN05001906	VC11903	Bcc110	P07	2003-05-30	Sputum	RAPD04	3.00	0.01648889	0	0.61	0.983606558	63	71	42	0.458030257	1.49E-05	0.455273166	1.64E-05
289138	SAMN05001907	VC13112	Bcc111	P07	2005-11-04	Sputum	RAPD04	22.66	0.02688889	1	0.571428572	0.764587525	66	84	0	0.43699506	1.83E-05	0.37940443	1.18E-05
289138	SAMN05001908	VC14761	Bcc112	P07	2009-04-27	Sputum	RAPD04	6.00	0.01934444	0	0.532710281	0.716049383	57	69	31	0.454123748	9.25E-06	0.488905716	1.54E-05
289138	SAMN05001909	VC14762	Bcc113	P07	2009-04-27	Sputum	RAPD04	12.75	0.04355556	0	0.544335512	0.923076923	57	69	31	0.431697979	1.64E-05	0.358180812	1.19E-05
289138	SAMN05001910	VC15305	Bcc114	P07	2010-06-28	Sputum	RAPD04	4.50	0.02064444	0	0.540540541	0.890625	57	69	31	0.449139846	9.21E-06	0.479527532	1.54E-05
289138	SAMN05001911	VC12201	Bcc115	P08	2004-01-12	Sputum	RAPD09	90.00	0.08613333	1	0.090669856	0.395492663	31	62	NA	0.42976379	1.84E-05	0.448611596	3.27E-05
289138	SAMN05001912	VC12308	Bcc116	P08	2004-03-23	Sputum	RAPD09	90.00	0.06345556	1	0.29791985	0.910447761	47	NA	NA	0.480153857	2.08E-05	0.502093842	3.53E-05
289138	SAMN05001913	VC12746	Bcc117	P08	2005-02-23	Sputum	RAPD09	90.00	0.08521111	0	0.377152052	0.967213115	42	79	NA	0.49676527	1.96E-05	0.451552629	2.65E-05
289138	SAMN05001914	VC12821	Bcc118	P08	2005-04-18	Sputum	RAPD09	84.00	0.08746667	0	0.869565218	1	57	98	NA	0.481600233	1.91E-05	0.449452558	2.82E-05
289138	SAMN05001915	VC12820	Bcc119	P08	2005-04-18	Sputum	RAPD09	88.00	0.12895556	0	0.035548387	0.055112571	57	98	NA	0.495725045	2.21E-05	0.484270468	3.37E-05
289138	SAMN05001916	VC13307	Bcc120	P08	2006-03-13	Sputum	RAPD09	26.00	0.05878889	0	0.016001235	0.078431373	51	98	NA	0.420379057	1.24E-05	0.437436324	1.82E-05
289138	SAMN05001917	VC13602	Bcc121	P08	2007-01-03	Sputum	RAPD09	3.50	0.03928333	0	0.01868323	0.018366465	46	81	13	0.454501474	1.96E-05	0.422195352	1.82E-05
289138	SAMN05001918	VC13600	Bcc122	P08	2007-01-03	Sputum	RAPD09	90.00	0.11616111	0	0.662790698	1	46	81	13	0.509038222	2.71E-05	0.50341585	3.47E-05
289138	SAMN05001919	VC14112	Bcc123	P08	2008-01-02	Sputum	RAPD09	85.00	0.15041667	0	0.594059406	1	51	75	NA	0.509087035	2.84E-05	0.485084156	3.46E-05
289138	SAMN05001920	VC14584	Bcc124	P08	2008-12-04	Sputum	RAPD09	2.50	0.05039445	0	0.004132232	0.012820513	48	78	15	0.462752999	1.92E-05	0.446350959	1.71E-05
289138	SAMN05001921	VC14801	Bcc125	P08	2009-06-04	Sputum	RAPD09	22.25	0.07528333	0	0.112244898	0.545454546	48	78	15	0.46528938	1.95E-05	0.448884515	1.74E-05
289138	SAMN05001922	VC14802	Bcc126	P08	2009-06-04	Sputum	RAPD09	3.00	0.05063889	0	0	0	48	78	15	0.461278529	1.86E-05	0.440829062	1.70E-05
289138	SAMN05001923	VC15266	Bcc127	P08	2010-06-02	Sputum	RAPD09	16.00	0.07306111	0	0.008474577	0.004201681	48	78	15	0.478157139	1.82E-05	0.421588974	1.64E-05
289138	SAMN05001924	VC15495	Bcc128	P08	2011-03-16	Sputum	RAPD09	5.00	0.02919444	0	0.016244571	NA	48	78	15	0.493644391	1.75E-05	0.466149955	1.91E-05
289138	SAMN05001925	VC7848	Bcc129	P09	1996-09-10	Sputum	RAPD44	90.00	0.02610556	1	1	1	97	89	118	0.431815789	2.86E-05	0.475047798	3.85E-05
289138	SAMN05001926	VC7849	Bcc130	P09	1996-09-10	Sputum	RAPD44	90.00	0.03736111	1	1	1	97	89	118	0.432743342	3.06E-05	0.517029608	4.06E-05
289138	SAMN05001927	VC7881	Bcc131	P09	1996-10-01	Throat	RAPD44	90.00	0.04789445	1	1	1	97	89	118	0.421737254	2.93E-05	0.480302706	3.83E-05

289138	SAMN05001928	VC8156	Bcc132	P09	1997-03-25	Sputum	RAPD44	90.00	0.03878333	1	1	1	97	89	118	0.436520316	2.85E-05	0.475006536	3.50E-05
289138	SAMN05001929	VC8672	Bcc133	P09	1998-02-17	Sputum	RAPD44	90.00	0.04337222	1	1	1	97	89	118	0.498964762	4.19E-05	0.575542868	3.41E-05
289138	SAMN05001930	VC9160	Bcc134	P09	1998-09-22	Sputum	RAPD44	90.00	0.03266111	1	1	1	97	89	118	0.506148769	3.98E-05	0.613440138	3.55E-05
289138	SAMN05001931	VC9958	Bcc135	P09	1999-11-23	Sputum	RAPD44	82.00	0.04868333	1	0.709639954	1	99	91	93	0.507512686	4.21E-05	0.528685726	3.15E-05
289138	SAMN05001932	VC12792	Bcc136	P09	2005-03-24	Sputum	RAPD15	90.00	0.03155	1	0.786331053	NA	81	76	86	0.467723709	4.04E-05	0.464555936	3.32E-05
289138	SAMN05001933	VC12802	Bcc137	P09	2005-03-27	Sputum	RAPD15	90.00	0.03371667	1	0.816224381	NA	94	89	104	0.46018701	3.88E-05	0.457047878	3.25E-05
289138	SAMN05001934	VC13476	Bcc138	P09	2006-08-03	Sputum	RAPD44	18.00	0.05081667	1	0.533146592	1	94	101	77	0.40233826	3.19E-05	0.430985452	2.35E-05
289138	SAMN05001935	VC14488	Bcc139	P09	2008-10-07	Sputum	RAPD44	36.50	0.08187222	1	0.46048746	NA	112	108	121	0.471538764	2.78E-05	0.43143855	1.55E-05
289138	SAMN05001936	VC15200	Bcc140	P09	2010-04-18		RAPD44	58.50	0.06183889	1	0.495908825	0.859275054	112	108	121	0.475320672	4.02E-05	0.459475901	2.85E-05
289138	SAMN05001937	VC15466	Bcc141	P09	2011-02-17	Sputum	RAPD44	35.00	0.07238333	1	0.675202547	NA	112	108	121	0.468578977	4.09E-05	0.451267606	2.68E-05
289138	SAMN05001938	VC6432	Bcc142	P10	1994-07-20	Sputum	RAPD06	90.00	0.37339445	1	0.327205883	0.888888889	52	72	27	0.43305379	2.27E-05	0.42127119	1.72E-05
289138	SAMN05001939	VC6456	Bcc143	P10	1994-08-01	Sputum	RAPD06	76.50	0.43423889	0	0.084120068	0.638297873	50	73	26	0.380241916	1.57E-05	0.34683727	1.14E-05
289138	SAMN05001940	VC6823	Bcc144	P10	1995-01-17	Sputum	RAPD06	78.00	0.29232778	1	0.082524272	0.58525	49	65	28	0.361059236	9.13E-06	0.389188395	1.64E-05
289138	SAMN05001941	VC7576	Bcc145	P10	1996-03-29	Sputum	RAPD06	90.00	0.30651111	NA	0.076923077	0.27686806	50	66	25	0.581559492	2.08E-05	0.404520431	1.48E-05
289138	SAMN05001942	VC7773	Bcc146	P10	1996-07-12	Sputum	RAPD06	90.00	0.30856667	NA	0.081790788	0.460474309	53	71	25	0.603499344	1.99E-05	0.423964416	1.37E-05
289138	SAMN05001943	VC8402	Bcc147	P10	1997-09-11	Sputum	RAPD06	30.50	0.28561111	0	0.008333333	0.063867244	42	62	15	NA	NA	0.554189584	6.71E-06
289138	SAMN05001944	VC9842	Bcc148	P10	1999-09-23	Sputum	RAPD06	68.25	0.34152222	0	0.005617978	0.179662922	40	58	14	0.546003953	2.18E-05	0.413189906	1.17E-05
289138	SAMN05001945	VC10567	Bcc149	P10	2001-01-11	Sputum	RAPD06	83.75	0.23722222	NA	0.053528457	0.170329671	36	55	13	0.616723665	2.50E-05	0.410586745	1.78E-05
289138	SAMN05001946	VC11138	Bcc150	P10	2001-12-21	Sputum	RAPD06	90.00	0.47572222	NA	0.048500882	0.35978836	36	46	14	0.496172223	1.14E-05	0.415425345	1.57E-05
289138	SAMN05001947	VC11747	Bcc151	P10	2003-02-14	Sputum	RAPD06	90.00	0.21126667	NA	0.03302851	0.317073171	33	47	10	0.582775687	1.90E-05	0.392965427	1.35E-05
289138	SAMN05001948	VC13124	Bcc152	P10	2005-11-09	Sputum	RAPD06	59.00	0.42351111	NA	0.139525368	NA	12	42	NA	0.638334331	1.21E-05	0.441276262	1.24E-05
289138	SAMN05001949	VC13178	Bcc153	P10	2005-12-20	Sputum	RAPD06	36.75	0.30994444	NA	0.038692254	NA	21	39	4	NA	NA	0.479027854	7.25E-06
289138	SAMN05001950	VC14358	Bcc154	P10	2008-06-25	Sputum	RAPD06	23.00	0.19625556	NA	0.069	0.312682642	73	72	69	NA	NA	0.413729856	1.12E-05
289138	SAMN05001951	VC6972	Bcc155	P11	1995-04-07	Sputum	RAPD02	13.00	0.04218889	0	0.048500882	0.289164693	29	50	11	0.534606337	2.27E-05	0.408821386	1.40E-05
289138	SAMN05001952	VC7473	Bcc156	P11	1996-01-26	Sputum	RAPD02	14.50	0.17692222	0	0.004201681	0.114583334	24	45	9	NA	NA	0.337658034	5.09E-06
289138	SAMN05001953	VC7718	Bcc157	P11	1996-06-12	Sputum	RAPD02	27.75	0.20884444	0	0.048500882	0.196305052	22	42	10	0.285120783	2.95E-06	0.375913818	8.36E-06
289138	SAMN05001954	VC7722	Bcc158	P11	1996-06-13	Sputum	RAPD02	16.50	0.07554444	0	0.032443937	0.555316864	22	42	10	NA	NA	0.155935392	3.33E-06
289138	SAMN05001955	VC7723	Bcc159	P11	1996-06-13	Sputum	RAPD02	5.00	0.1768	0	0.033955007	0.113927376	22	42	10	0.377293341	4.68E-06	0.325493142	6.87E-06
289138	SAMN05001956	VC7880	Bcc160	P11	1996-09-27	Sputum	RAPD02	18.50	0.30305556	0	0.045138889	0.163072167	23	49	10	0.452134529	2.01E-06	0.238873919	5.05E-06
289138	SAMN05001957	VC7879	Bcc161	P11	1996-09-27	Sputum	RAPD02	8.00	0.34958889	0	0.041887125	0.20327016	23	49	10	0.074643184	6.69E-06	0.409298845	1.12E-06
289138	SAMN05001958	VC8319	Bcc162	P11	1997-07-18	Sputum	RAPD02	4.50	0.04515556	0	0.027776357	0.246715687	26	54	9	0.335657484	6.03E-06	0.382213942	9.68E-06
289138	SAMN05001959	VC9443	Bcc163	P11	1999-01-14	Sputum	RAPD02	16.00	0.27505556	0	0.102100841	NA	11	32	3	0.892014992	7.21E-06	0.341268129	6.66E-06
289138	SAMN05001960	VC9444	Bcc164	P11	1999-01-14	Sputum	RAPD02	41.50	0.28537778	0	0.530973452	0.967741936	11	32	3	0.377245942	6.65E-06	0.372572044	8.72E-06
289138	SAMN05001961	VC4640	Bcc165	P12	1992-03-24	Sputum	RAPD02	71.50	0.12874445	0	0.01868323	0.44844872	77	84	65	0.422701182	8.89E-06	0.483532047	1.50E-05
289138	SAMN05001962	VC5080	Bcc166	P12	1993-02-02	Sputum	RAPD02	81.00	0.10695556	0	0.032443937	0.398749399	70	74	67	0.522751051	6.24E-06	0.447916736	1.31E-05
289138	SAMN05001963	VC5719	Bcc167	P12	1994-01-04	Sputum	RAPD02	78.00	0.09532222	0	0.032406417	0.316077441	68	79	49	NA	NA	0.420205096	1.11E-05
289138	SAMN05001964	VC6660	Bcc168	P12	1994-11-08	Sputum	RAPD02	85.00	0.11452222	0	0.042032086	0.348656786	77	87	63	0.456455732	7.77E-06	0.390080991	1.22E-05
289138	SAMN05001965	VC7393	Bcc169	P12	1995-10-30	Sputum	RAPD02	90.00	0.19997778	0	0.077947235	0.544	50	61	29	0.453441614	2.09E-05	0.392604169	1.32E-05
289138	SAMN05001966	VC7925	Bcc170	P12	1996-10-29	Sputum	RAPD02	90.00	0.21171667	0	0.20380824	0.515353353	54	63	34	0.472252593	2.56E-05	0.377009748	1.13E-05
289138	SAMN05001967	VC8412	Bcc171	P12	1997-09-16	Sputum	RAPD02	90.00	0.30248889	0	0.12672326	0.554108797	45	53	23	0.434489456	2.40E-05	0.390335349	1.40E-05
289138	SAMN05001968	VC9459	Bcc172	P12	1999-01-28	Sputum	RAPD02	80.00	0.29856667	0	0.042032086	0.53125	25	35	9	0.433012797	1.76E-05	0.417344493	1.32E-05
289138	SAMN05001969	VC1254	Bcc173	P13	1985-02-21	Sputum	RAPD01	43.50	0.21037778	0	0.0956875	0.573762839	NA	81	67	0.499544379	2.90E-05	0.433274185	2.10E-05
289138	SAMN05001970	VC1255	Bcc174	P13	1985-02-21	Sputum	RAPD01	27.50	0.37104444	0	0.042032086	0.307707708	NA	81	67	0.440138823	1.40E-05	0.425568587	1.53E-05
289138	SAMN05001971	VC4477	Bcc175	P13	1991-11-06	Sputum	RAPD01	45.33	0.08653333	0	0.036363637	0.324804549	28	40	12	0.49556803	2.50E-05	0.463146984	1.94E-05
289138	SAMN05001972	VC4896	Bcc176	P13	1992-10-04	Sputum	RAPD01	22.67	0.27252222	0	0.051632558	0.556277936	31	51	10	0.49647266	2.10E-05	0.459347665	1.71E-05
289138	SAMN05001973	VC5402	Bcc177	P13	1993-08-18	Sputum	RAPD01	33.00	0.22567778	0	0.055126165	0.546218488	27	46	8	0.4634247	2.06E-05	0.426168308	1.94E-05
289138	SAMN05001974	VC5506	Bcc178	P13	1993-10-24	Sputum	RAPD01	28.67	0.13221111	0	0.046118793	0.427509882	27	46	8	0.466810287	1.98E-05	0.427168241	1.84E-05
289138	SAMN05001975	VC5486	Bcc179	P13	1993-10-17	Sputum	RAPD01	34.00	0.0422	0	0.082517482	0.579449409	27	46	8	0.472349108	2.29E-05	0.427699607	1.71E-05
289138	SAMN05001976	VC13195	Bcc180	P14	2006-01-05	Sputum	RAPD02	14.00	0.13073333	0	0.036982363	NA	76	86	32	0.1646227	1.17E-06	0.319828572	5.80E-06
289138	SAMN05001977	VC13356	Bcc181	P14	2006-04-26	Sputum	RAPD02	19.33	0.17095556	0	0.036067537	NA	73	88	36	0.142274869	2.35E-06	0.351361505	3.51E-06
289138	SAMN05001978	VC13755	Bcc182	P14	2007-04-16	Sputum	RAPD02	13.50	0.07303333	0	0.005617978	0.043603314	74	89	38	0.195694811	2.30E-06	0.301295916	5.83E-06
289138	SAMN05001979	VC14309	Bcc183	P14	2008-05-12	Sputum	RAPD02	25.50	0.1355	0	0.0345231	NA	68	80	38	0.286511361	5.38E-06	0.342783565	5.00E-06
289138	SAMN05001980	VC14715	Bcc184	P14	2009-03-23	Sputum	RAPD02	31.00	0.22692222	0	0.010246856	0.012820513	68	80	38	0.120496762	2.47E-06	0.357228006	7.22E-06
289138	SAMN05001981	VC15185	Bcc185	P14	2010-04-03	Sputum	RAPD02	16.00	0.26224444	0	0.005050505	0.012820513	68	80	38	0.18894283	1.76E-06	0.323688992	6.62E-06
289138	SAMN05001982	VC15442	Bcc186	P14	2010-12-10	Blood	RAPD02	2.00	0.03745556	0	0.014501572	0.166745395	68	80	38	0.139214288	1.58E-06	0.41583448	7.12E-06
289138	SAMN05001983	VC6356	Bcc187	P15	1994-07-01	Sputum	RAPD02	54.50	0.21206667	0	0.253818182	0.7125	44	64	17	0.27167176	9.20E-06	0.33366069	1.31E-05
289138	SAMN05001984	VC6357	Bcc188	P15	1994-07-01	Sputum	RAPD02	13.00	0.22163333	0	0.18411045	0.645161291	44	64	17	0.300244139	1.07E-05	0.336020346	1.39E-05
289138	SAMN05001985	VC6590	Bcc189	P15	1994-10-07	Sputum	RAPD02	7.50	0.09893333	0	0.508853755	0.9375	42	67	12	0.273126366	1.06E-05	0.327686315	1.41E-05
289138	SAMN05001986	VC6589	Bcc190	P15	1994-10-07	Sputum	RAPD02	5.50	0.097	0	0.385206653	0.780888889	42	67	12	0.273843651	1.58E-05	0.367677481	1.32E-05
289138	SAMN05001987	VC7741	Bcc191	P15	1996-06-21	Sputum	RAPD02	10.00	0.09143333	0	0.279220779	0.586917293	13	26	5	0.213828073	9.66E-06	0.296781433	1.22E-05
289138	SAMN05001988	VC7933	Bcc192	P15	1996-11-04	Sputum	RAPD02	50.13	0.09863333	0	0.259287926	0.585858586	19	40	5	0.233433078	1.40E-05	0.344254832	1.19E-05
289138	SAMN05001989	VC7932	Bcc193	P15	1996-11-04	Sputum	RAPD02	4.00	0.03880556	NA	0.448568089	0.875266525	19	40	5	NA	NA	0.349759298	1.85E-05
289138	SAMN05001990	VC3917	Bcc201	P16	1990-10-05	Sputum	RAPD01	38.75	0.37935556	1	0.046756954	0.688842326	54	86	15	0.265048971	7.01E-06	0.450316149	2.62E-05
289138	SAMN05001991	VC4386	Bcc202	P16	1991-09-17	Sputum	RAPD01	45.38	0.37225556	0	0.035508022	0.200892451	45	66	13	0.288229605	8.52E-06	0.426346081	2.45E-05
289138	SAMN05001992	VC4895	Bcc203	P16	1992-10-04	Sputum	RAPD01	45.50	0.37775556	0	0.104482173	0.697339246	42	55	17	0.267185827	6.46E-06	0.420607537	2.29E-05
289138	SAMN05001993	VC5305	Bcc204	P16	1993-06-28	Sputum	RAPD01	42.25	0.38445556	0	0.741172552	0.967741936	30	45	10	0.284995449	7.62E-06	0.405775824	2.23E-05
289138	SAMN05001994	VC5617	Bcc205	P16	1993-12-01	Sputum	RAPD01	10.50	0.25915556	0	0.713968958	0.9375	27	42	9	0.308290605	9.82E-06	0.418810675	1.94E-05
289138	SAMN05001995	VC5618	Bcc206	P16	1993-12-01	Sputum	RAPD01	12.75	0.23432778	0	0.009899078	0.07755903	27	42	9	0.331985539	1.25E-05	0.427923562	2.18E-05
289138	SAMN05001996	VC6047	Bcc207	P16	1994-03-30	Sputum	RAPD01	3.00	0.16482222	0	0.014253526	0.00862069	36	51	14	0.366860484	1.50E-05	0.428001101	1.95E-05
289138	SAMN05001997	VC6901	Bcc208	P16	1995-02-28	Sputum	RAPD01	8.50	0.13566667	0	0.021689089	0.042803283	34	49	10	0.315309247	1.23E-05	0.389569745	1.47E-05
289138	SAMN05001998	VC6900	Bcc209	P16	1995-02-28	Sputum	RAPD01	2.00	0.13023333	0	0.035508022	0.05367647	34	49	10	0.35186236	1.86E-05	0.407616527	1.93E-05

289138	SAMN05001999	VC7526	Bcc210	P16	1996-03-05	Sputum	RAPD01	42.97	0.16914444	0	0.705882353	0.983606558	38	45	19	0.379842371	2.46E-05	0.404993118	2.72E-05
289138	SAMN05002000	VC7777	Bcc211	P16	1996-07-15	Sputum	RAPD01	37.00	0.26611111	0	0.740740741	0.983606558	36	44	13	0.392132247	2.32E-05	0.403085411	2.42E-05
289138	SAMN05002001	VC7964	Bcc212	P16	1996-11-21	Sputum	RAPD01	39.50	0.21774444	0	0.983606558	1	36	44	13	0.377135136	2.42E-05	0.377685474	2.48E-05
289138	SAMN05002002	VC7965	Bcc213	P16	1996-11-21	Sputum	RAPD01	2.00	0.0903	0	0.109025171	0.750712251	36	44	13	0.360734458	1.08E-05	0.345566595	8.48E-06
289138	SAMN05002003	VC8069	Bcc214	P16	1997-02-02	Sputum	RAPD01	6.50	0.19984444	0	0.027164213	0.004201681	36	44	13	0.296908343	9.36E-06	0.387909852	1.82E-05
289138	SAMN05002004	VC8068	Bcc215	P16	1997-02-02	Sputum	RAPD01	2.00	0.04828889	0	0.042803283	0.140740741	36	44	13	0.303175151	4.38E-06	0.307971042	2.91E-06
289138	SAMN05002005	VC8542	Bcc216	P16	1997-11-30	Sputum	RAPD01	4.00	0.16917778	0	0.172413793	0.670329671	0	0	0	0.387900067	2.00E-05	0.426840682	2.11E-05
289138	SAMN05002006	VC8865	Bcc217	P16	1998-04-28	Sputum	RAPD01	9.00	0.17612222	0	0.036363637	0.033634538	42	48	20	0.35587315	1.52E-05	0.486687569	1.69E-05
289138	SAMN05002007	VC9272	Bcc218	P16	1998-11-20	Sputum	RAPD01	4.00	0.11593333	NA	0.04177622	0.097142857	0	0	0	0.161737383	1.68E-06	0.227343035	2.12E-06
289138	SAMN05002008	VC9872	Bcc219	P16	1999-10-10	Sputum	RAPD01	12.50	0.11517778	0	0.016949153	0.026681012	19	26	5	0.4048227	1.92E-05	0.444725936	2.26E-05
289138	SAMN05002009	VC10022	Bcc220	P16	2000-01-04	Sputum	RAPD01	5.00	0.13081111	0	0.218786996	0.807692308	21	29	5	0.400004736	1.64E-05	0.554668664	1.74E-05
289138	SAMN05002010	VC10023	Bcc221	P16	2000-01-04	Sputum	RAPD01	4.50	0.13078889	NA	0.017241379	0.338983051	21	29	5	0.181868631	1.31E-06	NA	NA
289138	SAMN05002011	VC10414	Bcc222	P16	2000-10-05	Sputum	RAPD01	5.00	0.0178	1	0.020165356	0.037735849	19	22	5	0.427001422	1.77E-05	0.458212453	2.24E-05
289138	SAMN05002012	VC10425	Bcc223	P16	2000-10-22	Sputum	RAPD01	7.50	0.02665556	0	0.005617978	0.032406417	19	22	5	0.417709999	1.79E-05	0.482819167	2.30E-05

Table S3. Assembly statistics for **A.** Illumina short-read sequencing, **B.** PacBio long-read sequencing. For Illumina assemblies, the number of contigs, the draft assembly length, N50, and the GC content for each isolate are shown. For PacBio assemblies, Assembly statistics of PacBio long-read sequencing. The number of contigs, the draft assembly length, the number of Prokka-predicted genes, N50, average, minimum and maximum contig length,f Prokka-predicted genes, N50, average, minimum and maximum contig length, %GC, the total number of SNPs between the PacBio assembly and the respective Illumina assembly extracted by Mauve for each isolate, total number of SNPs at the ends of Illumina contigs and the total number of SNPs between the assemblies after filtering out SNPs at the ends of Illumina contigs are shown, and **C.** Number of SNPs identified by mapping Illumina reads against PacBio assemblies.

Column	Description
Patient_ID	Patient ID for each patient used in the laboratory
Isolate_ID	Sample ID for each isolate used in the laboratory
RAPD	RAPD genotyping as definited by the clinical microbiologist
Contigs	# of contigs in the Illumina draft assembly
Length	Total genome length in bp for the draft assembly
N50	N50 length in bp, i.e. the assembly statistics such at 50% of the entire assembly is contained in contigs equal to or larger than this value
GC	% GC content of the draft assembly
# of PacBio Contigs	# of contigs in the PacBio draft assembly
Genome Size	Total genome length in bp for the assembly
Average contig length	Average contig length in bp
Minimum contig length	Minimum contig length in bp
Maximum contig length	Maximum contig length in bp
# of Prokka Genes	# of genes prediced by the Prokka annotation tool
Total # of SNPs: PacBio & Illumina assemblies	# of SNPs between PacBio and Illumina assemblies extracted by Mauve
SNPs near Illumina contig ends	total # of SNPs at the ends (500bp from each side) of Illumina contigs
Final # of SNPs	Final # of SNPs by filtering out SNPs near Illumina contig ends from the total number of SNPs extracted by Mauve

Table S3A. Assembly statistics of Illumina short-read sequencing. The number of contigs, the draft assembly length, N50, and the GC content for each isolate are shown.

Isolate_ID	Contigs	Length	N50	GC	Coverage
Bcc001	118	7,925,783	121,515	0.67	73.72
Bcc002	137	8,305,511	105,657	0.67	63.82
Bcc003	120	8,303,544	112,468	0.67	68.83
Bcc004	122	7,713,702	120,954	0.67	87.97
Bcc005	138	7,918,418	120,886	0.67	62.89
Bcc006	159	7,821,186	94,026	0.67	54.36
Bcc007	122	7,994,972	131,698	0.67	89.22
Bcc008	129	7,799,151	110,853	0.67	88.56
Bcc009	122	7,754,666	137,603	0.67	131.17
Bcc010	136	8,128,047	125,676	0.67	72.36
Bcc011	118	8,130,961	121,127	0.67	95.61
Bcc012	103	7,657,866	141,329	0.67	106.47
Bcc013	131	7,903,817	120,844	0.67	84.30
Bcc014	135	7,716,780	118,221	0.67	72.46
Bcc015	125	7,990,792	131,863	0.67	71.51
Bcc016	136	8,216,635	121,280	0.67	105.42
Bcc017	122	7,878,229	116,654	0.67	81.33
Bcc018	142	8,089,212	112,340	0.67	68.27
Bcc019	115	8,052,732	131,696	0.67	90.32
Bcc020	142	8,243,697	115,468	0.67	68.78
Bcc021	136	7,881,538	118,491	0.67	97.18
Bcc022	163	7,863,896	100,125	0.67	60.22
Bcc023	199	8,439,130	73,659	0.67	65.36
Bcc024	153	8,280,796	111,824	0.67	107.74
Bcc025	167	8,223,766	84,257	0.67	65.57
Bcc026	233	8,160,310	55,387	0.67	52.41
Bcc027	183	8,167,271	73,688	0.67	65.94
Bcc028	183	8,338,440	80,564	0.67	67.66
Bcc029	168	8,205,144	101,052	0.67	98.38
Bcc030	173	7,023,674	70,907	0.67	61.72
Bcc031	193	8,064,782	74,464	0.67	60.21
Bcc032	181	8,100,261	84,243	0.67	83.64
Bcc033	174	8,208,614	89,769	0.67	115.08
Bcc034	211	8,302,918	83,040	0.67	76.09
Bcc035	299	8,418,157	55,472	0.67	67.04
Bcc036	194	7,993,094	88,512	0.67	122.74
Bcc037	193	8,029,511	78,731	0.67	93.04
Bcc038	436	8,033,464	70,164	0.67	58.24
Bcc039	208	7,842,096	73,364	0.67	101.81
Bcc040	185	8,010,235	73,702	0.67	53.57
Bcc041	357	8,217,675	48,395	0.67	59.35
Bcc042	202	8,256,386	71,985	0.67	54.11
Bcc043	151	8,105,853	91,959	0.67	74.37
Bcc044	242	8,135,266	56,961	0.67	57.96
Bcc045	159	8,561,276	101,584	0.67	203.46
Bcc046	173	8,030,651	108,235	0.67	269.38
Bcc047	169	8,082,095	89,605	0.67	93.15

Bcc048	154	8,059,037	95,310	0.67	208.78
Bcc049	543	8,170,914	78,682	0.67	73.82
Bcc050	158	8,075,701	103,074	0.67	158.89
Bcc051	184	7,834,535	85,082	0.67	84.24
Bcc052	221	7,840,548	58,311	0.67	57.02
Bcc053	207	8,034,416	79,930	0.67	67.31
Bcc054	207	7,974,837	61,790	0.67	61.34
Bcc055	202	8,255,412	78,997	0.67	70.78
Bcc056	210	8,057,352	70,824	0.67	61.98
Bcc057	185	8,108,607	83,337	0.67	448.01
Bcc058	151	7,779,068	89,009	0.67	162.48
Bcc059	151	8,194,825	89,785	0.67	79.91
Bcc060	175	8,073,471	94,354	0.67	82.12
Bcc061	134	7,659,765	104,372	0.67	85.30
Bcc062	152	7,793,949	87,115	0.67	85.10
Bcc063	161	8,272,729	81,549	0.67	83.96
Bcc064	181	7,605,111	76,756	0.67	64.95
Bcc065	187	7,671,778	81,539	0.67	65.20
Bcc066	175	7,896,156	79,762	0.67	71.63
Bcc067	181	7,927,174	78,990	0.67	54.80
Bcc068	201	7,736,663	65,777	0.67	54.14
Bcc069	182	7,735,064	85,814	0.67	62.97
Bcc070	133	7,737,583	94,697	0.67	188.83
Bcc071	155	8,054,231	100,351	0.67	115.23
Bcc072	133	7,700,203	102,545	0.67	165.74
Bcc073	120	8,195,834	130,815	0.67	58.29
Bcc074	114	8,422,831	136,893	0.67	80.91
Bcc075	140	8,461,196	114,220	0.67	74.28
Bcc076	333	8,367,352	157,262	0.67	187.61
Bcc077	108	7,963,420	141,210	0.67	109.84
Bcc078	190	8,033,083	94,448	0.67	53.12
Bcc079	113	8,653,941	131,332	0.67	94.77
Bcc080	132	8,188,200	113,325	0.67	69.12
Bcc081	179	8,456,224	88,487	0.67	55.56
Bcc082	99	8,289,318	143,914	0.67	94.98
Bcc083	116	8,561,670	133,135	0.67	80.32
Bcc084	122	8,886,003	131,690	0.67	85.39
Bcc085	229	8,166,374	72,702	0.67	47.41
Bcc086	195	8,233,364	75,059	0.67	61.79
Bcc087	125	8,087,599	126,366	0.67	66.22
Bcc088	178	7,873,839	84,362	0.67	48.35
Bcc089	1,997	10,297,781	78,888	0.59	66.82
Bcc091	142	7,774,669	120,769	0.67	58.87
Bcc092	145	8,018,150	117,375	0.67	64.35
Bcc093	162	7,788,896	100,450	0.67	64.91
Bcc094	172	7,459,900	85,298	0.67	518.82
Bcc095	175	8,265,967	111,294	0.67	61.14
Bcc096	108	6,864,411	131,456	0.67	113.39
Bcc097	262	7,810,896	117,692	0.67	68.46
Bcc098	2,535	8,666,072	103,977	0.67	481.90
Bcc099	1,901	8,088,620	102,332	0.66	229.61
Bcc100	6,673	9,787,116	3,308	0.66	20.03
Bcc101	248	9,416,391	90,804	0.66	47.65
Bcc102	167	9,419,006	105,313	0.66	61.73
Bcc103	284	8,705,557	102,977	0.66	54.44

Bcc104	190	8,829,720	99,773	0.66	44.97
Bcc105	127	9,221,948	160,923	0.66	105.60
Bcc106	146	8,967,713	146,184	0.66	70.79
Bcc107	97	8,391,151	150,987	0.67	103.74
Bcc108	136	8,444,245	146,919	0.67	73.06
Bcc109	122	8,702,300	130,788	0.67	73.05
Bcc110	116	8,329,494	123,952	0.67	76.95
Bcc111	117	8,487,941	131,539	0.67	89.25
Bcc112	138	8,424,239	109,400	0.67	58.33
Bcc113	120	8,657,486	130,711	0.67	87.48
Bcc114	132	8,409,845	123,819	0.67	70.70
Bcc115	94	6,673,164	225,783	0.67	63.55
Bcc116	88	7,813,367	216,937	0.67	89.42
Bcc117	128	7,792,758	107,684	0.67	65.12
Bcc118	111	7,703,210	146,759	0.67	57.67
Bcc119	101	7,807,554	166,724	0.67	62.21
Bcc120	84	7,717,990	231,020	0.67	116.53
Bcc121	79	7,795,263	176,502	0.67	76.67
Bcc122	67	7,858,397	227,329	0.67	99.69
Bcc123	59	8,210,251	247,014	0.67	115.05
Bcc124	71	7,567,024	214,586	0.67	95.35
Bcc125	110	7,863,310	157,950	0.67	73.88
Bcc126	141	7,120,785	116,190	0.67	57.91
Bcc127	109	6,849,782	111,824	0.67	71.85
Bcc128	119	7,218,535	125,263	0.67	55.34
Bcc129	142	7,408,917	146,773	0.67	93.41
Bcc130	161	7,668,323	153,958	0.67	83.15
Bcc131	161	7,137,591	105,373	0.67	63.07
Bcc132	121	7,713,115	156,011	0.67	79.77
Bcc133	106	7,516,515	178,409	0.67	100.55
Bcc134	173	7,701,741	99,509	0.67	50.74
Bcc135	177	7,379,544	107,584	0.67	56.36
Bcc136	88	7,510,056	251,057	0.67	101.26
Bcc137	81	7,469,615	208,967	0.67	26.91
Bcc138	122	7,484,159	162,477	0.67	134.28
Bcc139	127	7,558,564	156,495	0.67	76.27
Bcc140	169	7,900,058	121,186	0.67	91.00
Bcc141	970	7,375,795	15,152	0.67	172.19
Bcc142	1,330	7,779,238	11,777	0.67	92.10
Bcc143	332	7,742,779	48,175	0.67	54.97
Bcc144	198	7,705,763	78,900	0.67	47.82
Bcc145	218	7,812,933	67,560	0.67	43.66
Bcc146	565	7,684,045	26,000	0.67	29.97
Bcc147	137	7,790,525	121,306	0.67	108.05
Bcc148	120	7,693,636	120,565	0.67	84.17
Bcc149	128	7,872,484	132,801	0.67	81.06
Bcc150	178	7,707,408	83,771	0.67	52.34
Bcc151	190	7,868,321	91,116	0.67	50.56
Bcc152	258	7,643,517	57,172	0.67	39.74
Bcc153	206	7,910,337	82,948	0.67	45.23
Bcc154	294	7,711,338	50,458	0.67	54.59
Bcc155	473	8,060,818	31,597	0.67	45.31
Bcc156	715	7,953,760	21,678	0.67	44.32
Bcc157	218	8,191,316	69,039	0.67	62.24
Bcc158	298	8,150,235	49,062	0.67	50.13

Bcc159	173	8,054,440	78,613	0.67	72.84
Bcc160	246	8,115,564	65,767	0.67	70.27
Bcc161	162	8,030,352	82,615	0.67	74.00
Bcc162	217	8,025,032	71,120	0.67	67.86
Bcc163	1,385,308	240,139,281	161	0.63	35.83
Bcc164	316	8,022,808	42,054	0.67	82.53
Bcc165	185	8,260,220	79,353	0.67	89.34
Bcc166	218	8,031,736	64,968	0.67	62.22
Bcc167	204	8,061,899	71,572	0.67	61.86
Bcc168	262	8,140,388	57,241	0.67	46.53
Bcc169	203	8076230	78361	0.67	69.39
Bcc170	187	8103950	87245	0.67	62.62
Bcc171	190	7963481	78555	0.67	80.01
Bcc172	270	8,031,879	89,002	0.67	59.33
Bcc173	196	8,381,251	99,650	0.67	70.38
Bcc174	185	8,167,941	93,656	0.67	71.15
Bcc175	217	7,681,638	76,036	0.67	56.79
Bcc176	217	7,592,800	69,807	0.67	61.02
Bcc177	299	7,577,121	78,157	0.67	74.99
Bcc178	192	7,492,479	79,147	0.67	68.77
Bcc179	200	7,628,267	73,266	0.67	70.53
Bcc180	168	8264125	95594	0.67	82.18
Bcc181	158	8452801	95687	0.67	99.53
Bcc182	157	8165961	104195	0.67	145.98
Bcc183	260	8161135	69519	0.67	60.16
Bcc184	170	8258636	94511	0.67	128.89
Bcc185	215	8241262	76861	0.67	66.94
Bcc186	180	8413953	81911	0.67	72.44
Bcc187	144	8006336	108592	0.67	81.93
Bcc188	209	7813954	69897	0.67	55.93
Bcc189	170	7941719	81887	0.67	60.69
Bcc190	374	7994961	44830	0.67	62.52
Bcc191	136	7925989	105444	0.67	73.40
Bcc192	149	7935234	100795	0.67	60.10
Bcc193	124	7,868,150	114,015	0.67	71.66
Bcc201	1,012	8,363,008	104,235	0.67	61.62
Bcc202	181	8,553,945	87,513	0.67	57.69
Bcc203	153	8,337,406	101,435	0.67	63.13
Bcc204	165	8,291,207	101,641	0.67	85.54
Bcc205	169	7,549,072	80,924	0.67	66.62
Bcc206	170	7,816,083	85,365	0.67	70.83
Bcc207	245	8,344,095	61,797	0.67	46.31
Bcc208	183	8,362,730	83,759	0.67	88.19
Bcc209	172	8,338,450	104,077	0.67	100.18
Bcc210	207	8,645,242	110,280	0.67	91.48
Bcc211	152	8,132,784	104,003	0.67	87.50
Bcc212	168	8,426,237	94,457	0.67	161.88
Bcc213	179	8,507,419	101,231	0.67	107.20
Bcc214	171	8,278,844	94,530	0.67	130.84
Bcc215	211	8,387,313	81,509	0.67	65.69
Bcc216	222	8,277,648	64,241	0.67	52.35
Bcc217	193	8,200,519	80,989	0.67	82.08
Bcc218	227	8,289,567	59,846	0.67	56.12
Bcc219	198	8,034,938	75,986	0.67	120.61
Bcc220	240	8,373,076	61,117	0.67	58.13

Bcc221	197	8,253,340	76,892	0.67	85.87
Bcc222	205	8,182,231	83,573	0.67	80.75
Bcc223	207	8,039,157	72,006	0.67	83.41

Table S3B. Assembly statistics of PacBio long-read sequencing. The number of contigs, the draft assembly length, the number of Prokka-predicted genes, N50, average, minimum and maximum contig length, %GC, the total number of SNPs between the PacBio assembly and the respective Illumina assembly extracted by Mauve for each isolate, total number of SNPs at the ends of Illumina contigs and the total number of SNPs between the assemblies after filtering out SNPs at the ends of Illumina contigs are shown.

Patient_ID	Isolate_ID	RAPD	# of PacBio Contigs	Genome Size	# of Prokka Genes	N50_contig_length	Average contig length	Min contig length	Max contig length	%GC	Total # of SNPs: PacBio & Illumina assemblies	SNPs near Illumina contig ends	Final # of SNPs
P01	Bcc001	RAPD06	4	7915579	7142	6028290	1978895	14146	6028290	66.95	79	61	18
P01	Bcc022	RAPD06	7	7803519	7047	2732770	1114788	10137	3076554	66.90	47	35	12
P02	Bcc023	RAPD04	9	8228891	7380	3296054	914321	10587	3394186	67.00	74	33	41
P02	Bcc030	RAPD04	5	7094839	6381	2018644	1418968	212231	3297374	66.91	87	63	24
P08	Bcc116	RAPD09	4	7634498	6786	2984720	1908625	17257	3668000	67.15	215	55	160
P09	Bcc129	RAPD44	1	7499459	6706	7499459	7499459	7499459	7499459	66.92	24	17	7
P09	Bcc137	RAPD15	2	7394909	6550	6339862	3697455	1055047	6339862	67.05	118	109	9
P13	Bcc173	RAPD01	4	8177267	7360	3156709	2044317	191387	3818057	66.97	32	22	10
P13	Bcc174	RAPD01	17	8373634	7526	2762452	492567	9072	3188294	66.88	95	69	26
P13	Bcc179	RAPD01	10	7736143	6961	2769789	773614	7478	2920426	66.89	237	146	91
P16	Bcc201	RAPD01	13	7344673	6643	2494342	564975	3768	3197438	66.81	397	238	159

Reference	Isolate	RAPD	Contigs	Genome Size	# of Prokka Genes	N50_contig_length	Average contig length	Min contig length	Max contig length	%GC	# of SNPs between PacBio and NCBI Reference
NCBI	J2315	RAPD02	4	8016291	7261	3217061	2004073	92401	3830852	66.91	10

Table S3C. Number of SNPs identified by mapping Illumina reads against PacBio assemblies. The total number of unfiltered SNPs and final number of SNPs after filtering out for low quality SNPs (see Supplemental Methods) are shown.

Patient_ID	Isolate_ID	RAPD	# of PacBio Contigs	Genome Size	# of unfiltered SNPs	Final # of SNPs
P01	Bcc001	RAPD06	4	7915579	119	0
P02	Bcc023	RAPD04	9	8228891	38	0
P08	Bcc116	RAPD09	4	7634498	4	0
P09	Bcc129	RAPD44	1	7499459	1	0
P09	Bcc137	RAPD15	2	7394909	79	0
P13	Bcc173	RAPD01	4	8177267	52	0

Table S4. Multi-locus sequence type (MLST) of each B. cenocepacia isolates as identified by SRST2 (https://github.com/katholt/srst2).

Column	Description
Isolate_ID	Sample ID for each isolate used in the laboratory
ST	the best scoring sequence type for *B. cenocepacia* as predicted by SRST2
atpD	*atpD*, one of the *B. cenocepacia* MLST locus, and the best scoring allele
gltB	*gltB,* one of the *B. cenocepacia* MLST locus, and the best scoring allele
gyrB	*gyrB*, one of the *B. cenocepacia* MLST locus, and the best scoring allele
recA	*recA*, one of the *B. cenocepacia* MLST locus, and the best scoring allele
lepA	*lepA*, one of the *B. cenocepacia* MLST locus, and the best scoring allele
phaC	*phaC,* one of the *B. cenocepacia* MLST locus, and the best scoring allele
trpB	*trpB,* one of the *B. cenocepacia* MLST locus, and the best scoring allele
mismatches	details of any mismatches
uncertainty	details when the depth of coverage was too low to be confident in the result
depth	mean read depth across the length of all alleles assigned a top scoring allele number
maxMAF	highest minor allele frequency of variants found for MLST locus alignments
*	indicates that the best scoing allele has >= 1 mismatch
?	indicates uncertainty in the result since the best scoring allele has low-depth bases
-	no allele could be assigned
NF	no known ST type has been assigned to the allele combination

Table S4. Multi-locus sequence type (MLST) of each B. cenocepacia isolates as identified by SRST2 (https://github.com/katholt/srst2).

Isolate_ID	ST	atpD	gltB	gyrB	recA	lepA	phaC	trpB	mismatches	uncertainty	depth	maxMAF
Bcc001	210	15	11	180	14	11	6	79	0	-	38.3144286	0.15555556
Bcc002	210	15	11	180	14	11	6	79	0	-	37.8717143	0.18181818
Bcc003	210	15	11	180	14	11	6	79	0	-	38.5521429	0.16216216
Bcc004	210	15	11	180	14	11	6	79	0	-	51.197	0.16
Bcc005	210	15	11	180	14	11	6	79	0	-	34.2338571	0.18181818
Bcc006	210	15	11	180	14	11	6	79	0	-	29.4114286	0.16
Bcc007	210	15	11	180	14	11	6	79	0	-	48.9484286	0.14814815
Bcc008	210	15	11	180	14	11	6	79	0	-	50.6998571	0.12280702
Bcc009	210	15	11	180	14	11	6	79	0	-	66.9604286	0.19672131
Bcc010	210	15	11	180	14	11	6	79	0	-	42.2835714	0.18181818
Bcc011	210	15	11	180	14	11	6	79	0	-	51.0894286	0.13333333
Bcc012	210	15	11	180	14	11	6	79	0	-	56.743	0.13432836
Bcc013	210	15	11	180	14	11	6	79	0	-	48.9812857	0.13888889
Bcc014	210	15	11	180	14	11	6	79	0	-	40.208	0.13513514
Bcc015	210	15	11	180	14	11	6	79	0	-	36.1234286	0.15
Bcc016	210	15	11	180	14	11	6	79	0	-	54.9352857	0.16071429
Bcc017	210	15	11	180	14	11	6	79	0	-	41.7718571	0.125
Bcc018	210	15	11	180	14	11	6	79	0	-	35.4784286	0.15384615
Bcc019	210	15	11	180	14	11	6	79	0	-	44.7518571	0.15555556
Bcc020	210	15	11	180	14	11	6	79	0	-	35.8617143	0.17241379
Bcc021	210	15	11	180	14	11	6	79	0	-	53.1702857	0.15909091
Bcc022	210	15	11	180	14	11	6	79	0	-	31.8111429	0.20689655
Bcc023	234	15	11	182	14	11	6	147	0	-	34.0155714	0.23333333
Bcc024	234	15	11	182	14	11	6	147	0	-	61.2035714	0.14583333
Bcc025	234	15	11	182	14	11	6	147	0	-	35.9445714	0.2
Bcc026	234	15	11	182	14	11	6	147	0	-	32.019	0.16666667
Bcc027	234	15	11	182	14	11	6	147	0	-	35.5162857	0.13953488
Bcc028	234	15	11	182	14	11	6	147	0	-	38.892	0.20930233
Bcc029	234	15	11	182	14	11	6	147	0	-	57.0981429	0.13333333
Bcc030	234	15	11	182	14	11	6	147	0	-	35.9944286	0.16666667
Bcc031	234	15	11	182	14	11	6	147	0	-	32.6395714	0.18518519
Bcc032	234	15	11	182	14	11	6	147	0	-	50.6532857	0.11764706
Bcc033	234	15	11	182	14	11	6	147	0	-	58.3274286	0.11764706
Bcc034	234	15	11	182	14	11	6	147	0	-	44.0744286	0.25
Bcc035	234	15	11	182	14	11	6	147	0	-	43.466	0.11111111
Bcc036	234	15	11	182	14	11	6	147	0	-	63.397	0.23684211
Bcc037	234	15	11	182	14	11	6	147	0	-	47.3267143	0.2
Bcc038	234	15	11	182	14	11	6	147	0	-	26.7044286	0.15384615
Bcc039	234	15	11	182	14	11	6	147	0	-	50.2908571	0.16216216
Bcc040	234	15	11	182	14	11	6	147	0	-	25.506	0.17647059
Bcc041	234	15	11	182	14	11	6	147	0	-	35.5537143	0.2173913
Bcc042	234	15	11	182	14	11	6	147	0	-	30.4054286	0.16666667
Bcc043	234	15	11	182	14	11	6	147	0	-	34.0122857	0.125
Bcc044	234	15	11	182	14	11	6	147	0	-	33.848	0.14814815
Bcc045	234	15	11	182	14	11	6	147	0	-	112.710286	0.13333333
Bcc046	NF*	136*	11	581*	14	271*	6	79*	atpD_136/3snp;gyrB_581/12snp;lepA_271/1snp;trpB_79/1snp	-	139.705143	0.14018692
Bcc047	234	15	11	182	14	11	6	147	0	-	50.2902857	0.16216216
Bcc048	NF*	132*	11	182	14	11	6	147	atpD_132/1snp	-	120.361286	0.14678899
Bcc049	234	15	11	182	14	11	6	147	0	-	46.1752857	0.1
Bcc050	234	15	11	182	14	11	6	147	0	-	92.2197143	0.11392405
Bcc051	234	15	11	182	14	11	6	147	0	-	49.8357143	0.12765957
Bcc052	234?	15	11	182	14	11	6?	147	0	phaC_6/edge	26.6662857	0.14285714
Bcc053	234	15	11	182	14	11	6	147	0	-	37.8317143	0.13793103
Bcc054	234	15	11	182	14	11	6	147	0	-	34.0818571	0.19230769
Bcc055	234	15	11	182	14	11	6	147	0	-	39.8771429	0.15789474
Bcc056	234	15	11	182	14	11	6	147	0	-	35.6308571	0.16666667
Bcc057	NF*	152*	87*	574*	143*	195*	168*	270*	atpD_152/24snp;gltB_87/12snp;gyrB_574/10snp;recA_143/1snp;lepA_195/8snp;phaC_168/9snp;trpB_270/12snp	-	211.526286	0.07333333
Bcc058	28	15	11	9	14	11	6	12	0	-	93.8734286	0.2247191
Bcc059	28	15	11	9	14	11	6	12	0	-	45.4571429	0.16666667
Bcc060	28	15	11	9	14	11	6	12	0	-	21.8297143	0.2
Bcc061	28	15	11	9	14	11	6	12	0	-	49.6864286	0.10869565
Bcc062	28	15	11	9	14	11	6	12	0	-	42.3735714	0.14285714
Bcc063	28	15	11	9	14	11	6	12	0	-	46.6608571	0.16
Bcc064	28	15	11	9	14	11	6	12	0	-	33.6772857	0.14285714
Bcc065	28	15	11	9	14	11	6	12	0	-	37.77	0.13333333
Bcc066	28	15	11	9	14	11	6	12	0	-	42.357	0.10714286
Bcc067	28	15	11	9	14	11	6	12	0	-	30.6328571	0.2
Bcc068	28	15	11	9	14	11	6	12	0	-	31.2804286	0.17142857
Bcc069	28	15	11	9	14	11	6	12	0	-	35.2842857	0.25
Bcc070	28	15	11	9	14	11	6	12	0	-	110.664429	0.20325203

Bcc071	28	15	11	9	14	11	6	12		0	-	62.3851429	0.1875
Bcc072	28	15	11	9	14	11	6	12		0	-	84.295	0.12857143
Bcc073	278	15	183	182	14	11	6	147		0	-	31.0917143	0.14285714
Bcc074	278	15	183	182	14	11	6	147		0	-	44.0677143	0.12195122
Bcc075	278	15	183	182	14	11	6	147		0	-	49.2591429	0.16666667
Bcc076	NF*	308*	183	182	14	11	6	147	atpD_308/1snp		-	114.038143	0.11688312
Bcc077	278	15	183	182	14	11	6	147		0	-	66.524	0.13793103
Bcc078	278	15	183	182	14	11	6	147		0	-	30.3142857	0.14285714
Bcc079	278	15	183	182	14	11	6	147		0	-	14.3027143	0.25
Bcc080	278	15	183	182	14	11	6	147		0	-	42.0651429	0.13953488
Bcc081	278	15	183	182	14	11	6	147		0	-	30.4735714	0.24137931
Bcc082	278	15	183	182	14	11	6	147		0	-	50.7698571	0.11320755
Bcc083	278	15	183	182	14	11	6	147		0	-	42.8832857	0.20588235
Bcc084	278	15	183	182	14	11	6	147		0	-	48.4412857	0.11494253
Bcc085	278	15	183	182	14	11	6	147		0	-	25.5821429	0.16666667
Bcc086	278	15	183	182	14	11	6	147		0	-	42.5595714	0.28571429
Bcc087	210	15	11	180	14	11	6	79		0	-	26.6191429	0.26666667
Bcc088	210	15	11	180	14	11	6	79		0	-	24.625	0.28571429
Bcc089	210	15	11	180	14	11	6	79		0	-	38.2118571	0.13953488
Bcc091	210	15	11	180	14	11	6	79		0	-	17.218	0.2
Bcc092	210	15	11	180	14	11	6	79		0	-	39.2224286	0.14705882
Bcc093	210	15	11	180	14	11	6	79		0	-	37.969	0.17307692
Bcc094	NF*	270*	394*	302*	29*	195*	86*	104*	atpD_270/35snp;gltB_394/1snp;gyrB_302/21snp1indel;recA_29/28snp;lepA_195/8snp;phaC_86/22snp;trpB_104/23snp		-	303.269286	0.05120482
Bcc095	210	15	11	180	14	11	6	79		0	-	31.031	0.15789474
Bcc096	210	15	11	180	14	11	6	79		0	-	43.4377143	0.12903226
Bcc097	210	15	11	180	14	11	6	79		0	-	25.9457143	0.16666667
Bcc098	NF*	232*	396*	43*	143*	191*	85*	368*	atpD_232/21snp;gltB_396/11snp1indel;gyrB_43/21snp;recA_143/1snp;lepA_191/7snp;phaC_85/5snp;trpB_368/3snp		-	233.163143	0.07692308
Bcc099	210	15	11	180	14	11	6	79		0	-	91.595	0.16
Bcc100	234*?	15	11	182*?	14	11?	6?	147	gyrB_182/1snp		gyrB_182/ed	8.46814286	0.5
Bcc101	234	15	11	182	14	11	6	147		0	-	18.5174286	0.2631579
Bcc102	234	15	11	182	14	11	6	147		0	-	31.237	0.14285714
Bcc103	234	15	11	182	14	11	6	147		0	-	31.576	0.15625
Bcc104	234	15	11	182	14	11	6	147		0	-	21.6925714	0.23076923
Bcc105	234	15	11	182	14	11	6	147		0	-	52.2041429	0.13888889
Bcc106	234	15	11	182	14	11	6	147		0	-	40.3512857	0.22580645
Bcc107	234	15	11	182	14	11	6	147		0	-	55.8252857	0.14634146
Bcc108	234	15	11	182	14	11	6	147		0	-	36.9365714	0.15
Bcc109	234	15	11	182	14	11	6	147		0	-	42.8448571	0.12765957
Bcc110	234	15	11	182	14	11	6	147		0	-	39.5777143	0.11320755
Bcc111	234	15	11	182	14	11	6	147		0	-	50.8445714	0.11627907
Bcc112	234	15	11	182	14	11	6	147		0	-	31.1911429	0.13793103
Bcc113	234	15	11	182	14	11	6	147		0	-	52.0332857	0.175
Bcc114	234	15	11	182	14	11	6	147		0	-	41.851	0.12
Bcc115	306	15	64	284	14	11	6	147		0	-	39.0091429	0.16129032
Bcc116	306	15	64	284	14	11	6	147		0	-	50.5564286	0.14285714
Bcc117	306	15	64	284	14	11	6	147		0	-	34.3878571	0.13793103
Bcc118	306	15	64	284	14	11	6	147		0	-	30.405	0.18181818
Bcc119	306	15	64	284	14	11	6	147		0	-	24.6925714	0.21428571
Bcc120	NF*	15	64	284	14	271*	6	147	lepA_271/1snp		-	82.4151429	0.109375
Bcc121	306	15	64	284	14	11	6	147		0	-	53.3047143	0.12195122
Bcc122	306	15	64	284	14	11	6	147		0	-	53.3788571	0.21428571
Bcc123	306	15	64	284	14	11	6	147		0	-	53.1855714	0.11428571
Bcc124	306	15	64	284	14	11	6	147		0	-	66.5228571	0.12
Bcc125	306	15	64	284	14	11	6	147		0	-	19.9397143	0.42857143
Bcc126	306	15	64	284	14	11	6	147		0	-	26.3478571	0.17647059
Bcc127	306	15	64	284	14	11	6	147		0	-	51.2475714	0.13513514
Bcc128	306	15	64	284	14	11	6	147		0	-	32.6305714	0.19230769
Bcc129	224	23	136	192	49	155	8	144		0	-	14.0498571	0.375
Bcc130	224	23	136	192	49	155	8	144		0	-	52.6707143	0.18518519
Bcc131	224	23	136	192	49	155	8	144		0	-	22.314	0.28571429
Bcc132	224	23	136	192	49	155	8	144		0	-	41.719	0.21212121
Bcc133	224	23	136	192	49	155	8	144		0	-	62.6241429	0.15873016
Bcc134	224	23	136	192	49	155	8	144		0	-	27.2671429	0.17241379
Bcc135	224	23	136	192	49	155	8	144		0	-	32.5794286	0.20588235
Bcc136	267	159	173	220	136	186	140	176		0	-	60.1004286	0.2
Bcc137	267	159	173	220	136	186	140	176		0	-	84.1184286	0.13157895
Bcc138	224	23	136	192	49	155	8	144		0	-	19.6397143	0.16666667
Bcc139	224	23	136	192	49	155	8	144		0	-	41.9675714	0.2
Bcc140	224	23	136	192	49	155	8	144		0	-	22.5181429	0.23076923
Bcc141	NF*	79*	136	192	49	155	8	144	atpD_79/2snp		-	111.377857	0.07692308
Bcc142	210	15	11	180	14	11	6	79		0	-	18.2065714	0.22222222
Bcc143	210?	15	11	180	14	11?	6	79		0	lepA_11/edg	24.279	0.14814815
Bcc144	210	15	11	180	14	11	6	79		0	-	27.0174286	0.23076923
Bcc145	210	15	11	180	14	11	6	79		0	-	19.4157143	0.25
Bcc146	210	15	11	180	14	11	6	79		0	-	17.9395714	0.2
Bcc147	210	15	11	180	14	11	6	79		0	-	25.1078571	0.2

Bcc148	210	15	11	180	14	11	6	79		0	-	43.61	0.125
Bcc149	210	15	11	180	14	11	6	79		0	-	45.9752857	0.17391304
Bcc150	210	15	11	180	14	11	6	79		0	-	29.9031429	0.16666667
Bcc151	210	15	11	180	14	11	6	79		0	-	30.4007143	0.0952381
Bcc152	210	15	11	180	14	11	6	79		0	-	15.1061429	0.18181818
Bcc153	210	15	11	180	14	11	6	79		0	-	22.057	0.18181818
Bcc154	210?	15	11	180	14	11?	6	79		0	lepA_11/edg	17.6328571	0.33333333
Bcc155	28	15	11	9	14	11	6	12		0	-	21.5471429	0.1875
Bcc156	28	15	11	9	14	11	6	12		0	-	20.7692857	0.28571429
Bcc157	28	15	11	9	14	11	6	12		0	-	30.6174286	0.13333333
Bcc158	28	15	11	9	14	11	6	12		0	-	24.8261429	0.25
Bcc159	28	15	11	9	14	11	6	12		0	-	38.4138571	0.16666667
Bcc160	28	15	11	9	14	11	6	12		0	-	42.7535714	0.13513514
Bcc161	28	15	11	9	14	11	6	12		0	-	33.8421429	0.16666667
Bcc162	28	15	11	9	14	11	6	12		0	-	30.9495714	0.15
Bcc163	NF*?	15	159*	67*?	368*?	11	105*	29*?	gltB_159/1snp;gyrB_67/23snp28holes;recA_368/46snp2indel19holes;phaC_105/1snp;trpB_29/19snp21holes		gyrB_67/edg	14.5857143	0.5
Bcc164	28*	15	11*	9	14	11	6	12	gltB_11/1snp		-	50.621	0.10714286
Bcc165	28	15	11	9	14	11	6	12		0	-	51.8461429	0.16666667
Bcc166	28	15	11	9	14	11	6	12		0	-	33.7185714	0.14285714
Bcc167	28	15	11	9	14	11	6	12		0	-	29.2977143	0.15
Bcc168	28	15	11	9	14	11	6	12		0	-	22.2667143	0.2
Bcc169	28?	15	11?	9	14?	11	6?	12		0	gltB_11/edge	12.1867143	0.33333333
Bcc170	28	15	11	9	14	11	6	12		0	-	20.2827143	0.14285714
Bcc171	28	15	11	9	14	11	6	12		0	-	25.4718571	0.23809524
Bcc172	28?	15	11	9	14?	11	6	12		0	recA_14/edg	21.1708571	0.14285714
Bcc173	32	16	11	10	14	11	6	79		0	-	15.4522857	0.15384615
Bcc174	NF	16	11	10	14	-	6	79		0	-	17.7636667	0.21052632
Bcc175	32	16	11	10	14	11	6	79		0	-	12.9078571	0.28571429
Bcc176	32	16	11	10	14	11	6	79		0	-	17.3162857	0.25
Bcc177	32	16	11	10	14	11	6	79		0	-	23.0248571	0.16666667
Bcc178	32	16	11	10	14	11	6	79		0	-	21.9895714	0.2
Bcc179	32	16	11	10	14	11	6	79		0	-	20.0742857	0.11764706
Bcc180	28	15	11	9	14	11	6	12		0	-	24.9574286	0.1875
Bcc181	28	15	11	9	14	11	6	12		0	-	22.3714286	0.18181818
Bcc182	28	15	11	9	14	11	6	12		0	-	42.7078571	0.125
Bcc183	28*?	15?	11	9	14	11?	6*?	12	phaC_6/4holes		atpD_15/edg	7.23157143	0.5
Bcc184	28	15	11	9	14	11	6	12		0	-	45.9074286	0.17142857
Bcc185	28?	15	11	9	14	11?	6	12		0	lepA_11/edg	12.1067143	0.33333333
Bcc186	28*?	15	11	9	14	11*?	6	12	lepA_11/4holes		lepA_11/edg	19.8654286	0.13793103
Bcc187	28	15	11	9	14	11	6	12		0	-	22.2558571	0.125
Bcc188	28	15	11	9	14	11	6	12		0	-	12.6232857	0.16666667
Bcc189	28	15	11	9	14	11	6	12		0	-	13.5001429	0.25
Bcc190	28*?	15	11	9	14	11*?	6	12*?	lepA_11/22holes;trpB_12/17holes		lepA_11/edg	8.90728571	0.25
Bcc191	28	15	11	9	14	11	6	12		0	-	26.276	0.1
Bcc192	28	15	11	9	14	11	6	12		0	-	20.3198571	0.25
Bcc193	28	15	11	9	14	11	6	12		0	-	19.9727143	0.1875
Bcc201	33	16	11	10	95	11	6	79		0	-	36.3892857	0.14814815
Bcc202	33	16	11	10	95	11	6	79		0	-	21.5585714	0.27272727
Bcc203	33	16	11	10	95	11	6	79		0	-	39.0151429	0.12
Bcc204	33	16	11	10	95	11	6	79		0	-	57.8125714	0.15384615
Bcc205	33	16	11	10	95	11	6	79		0	-	42.597	0.15384615
Bcc206	33	16	11	10	95	11	6	79		0	-	43.7227143	0.16216216
Bcc207	33	16	11	10	95	11	6	79		0	-	27.34	0.18181818
Bcc208	33	16	11	10	95	11	6	79		0	-	58.2217143	0.12307692
Bcc209	33	16	11	10	95	11	6	79		0	-	66.9238571	0.13114754
Bcc210	33	16	11	10	95	11	6	79		0	-	61.8712857	0.08928571
Bcc211	33	16	11	10	95	11	6	79		0	-	53.8342857	0.09615385
Bcc212	33	16	11	10	95	11	6	79		0	-	104.225571	0.10416667
Bcc213	33	16	11	10	95	11	6	79		0	-	22.9927143	0.16
Bcc214	33	16	11	10	95	11	6	79		0	-	71.5511429	0.10606061
Bcc215	33	16	11	10	95	11	6	79		0	-	21.2284286	0.16666667
Bcc216	33	16	11	10	95	11	6	79		0	-	30.7091429	0.13333333
Bcc217	33	16	11	10	95	11	6	79		0	-	53.226	0.1025641
Bcc218	33	16	11	10	95	11	6	79		0	-	35.282	0.15384615
Bcc219	33	16	11	10	95	11	6	79		0	-	76.5734286	0.1125
Bcc220	33	16	11	10	95	11	6	79		0	-	37.8274286	0.16666667
Bcc221	33	16	11	10	95	11	6	79		0	-	50.7595714	0.17241379
Bcc222	33	16	11	10	95	11	6	79		0	-	54.8224286	0.09677419
Bcc223	33	16	11	10	95	11	6	79		0	-	52.6841429	0.125

Table S5. PacBio identification of methylation motifs.

Column	Description
Patient_ID	Patient ID for each patient used in the laboratory
Isolate_ID	Sample ID for each isolate used in the laboratory
RAPD	RAPD genotyping as definited by the clinical microbiologist
Methyltransferase_activity	The methylated motif
Methylation_position	The methylated position within the motif
Methylation_type	The methylated base
Fraction_of_methylation	Fraction of motifs that are methylated (methylated_sites/total_sites)
Methylated_sites	Total number of motifs that are methylated
Total_sites	Total number of potential methylation motifs
meanCoverage	The coverage

Table S5. PacBio identification of methylation motifs.

Patient_ID	Isolate_ID	RAPD	Methyltransferase_activity	Methylation_position	Methylation_type	Fraction_of_methylation	Methylated_sites	Total_sites	meanCoverage
P01	Bcc001	RAPD06	CACAG	4	m6A	0.99586594	6745	6773	50.52276
P01	Bcc001	RAPD06	TGAANNNNNNNCTC	4	m6A	0.9941691	1023	1029	50.012707
P01	Bcc001	RAPD06	GAGNNNNNNNTTCA	2	m6A	0.9902818	1019	1029	50.880276
P01	Bcc001	RAPD06	GTWWAC	5	m6A	0.97327393	1748	1796	48.85469
P01	Bcc001	RAPD06	GGGCCC	5	m4C	0.9568546	2750	2874	51.848362
P01	Bcc022	RAPD06	TGAANNNNNNNCTC	4	m6A	0.99507874	1011	1016	47.88823
P01	Bcc022	RAPD06	GAGNNNNNNNTTCA	2	m6A	0.984252	1000	1016	48.764
P01	Bcc022	RAPD06	CACAG	4	m6A	0.99044776	6636	6700	48.75407
P01	Bcc022	RAPD06	GTWWAC	5	m6A	0.97042096	1706	1758	47.597305
P01	Bcc022	RAPD06	GGGCCC	5	m4C	0.9267516	2619	2826	49.67545
P02	Bcc023	RAPD04	CCANNNNNNNCTGC	3	m6A	0.993524	1841	1853	50.531776
P02	Bcc023	RAPD04	GCAGNNNNNNNTGG	3	m6A	0.99190503	1838	1853	50.55767
P02	Bcc023	RAPD04	CACAG	4	m6A	0.9931884	6853	6900	49.77601
P02	Bcc023	RAPD04	GTWWAC	5	m6A	0.97176594	1893	1948	47.643425
P02	Bcc030	RAPD04	CCANNNNNNNCTGC	3	m6A	0.9968711	1593	1598	50.07219
P02	Bcc030	RAPD04	GCAGNNNNNNNTGG	3	m6A	0.9962453	1592	1598	49.56784
P02	Bcc030	RAPD04	CACAG	4	m6A	0.9922585	5896	5942	49.618217
P02	Bcc030	RAPD04	GTWWAC	5	m6A	0.9631391	1620	1682	47.057407
P08	Bcc116	RAPD09	CACAG	4	m6A	0.99825454	6291	6302	49.11445
P08	Bcc116	RAPD09	CCCGAG	5	m6A	0.99163514	4979	5021	49.79474
P08	Bcc116	RAPD09	GTWWAC	5	m6A	0.971564	1640	1688	47.758537
P09	Bcc129	RAPD44	CACAG	4	m6A	0.98545915	6235	6327	32.168724
P09	Bcc129	RAPD44	CTCGAG	5	m6A	0.97664315	7192	7364	32.299637
P09	Bcc129	RAPD44	GTWWAC	5	m6A	0.9527421	1633	1714	31.22719
P09	Bcc129	RAPD44	BNRCGGCCGY	7	m4C	0.056365576	634	11248	37.135647
P09	Bcc137	RAPD15	CACAG	4	m6A	0.99967724	6194	6196	70.13755
P09	Bcc137	RAPD15	GTWWAC	5	m6A	0.97451454	1606	1648	68.19739
P09	Bcc137	RAPD15	TCGWCGA	5	m4C	0.9265009	17963	19388	71.77766
P09	Bcc137	RAPD15	BCTCGAGV	6	m6A	0.8951382	5634	6294	70.1143
P09	Bcc137	RAPD15	ACTCGAGS	6	m6A	0.39095744	147	376	70.29932
P09	Bcc137	RAPD15	BCTCGAGTNNNNS	6	m6A	0.3442623	126	366	70.2381
P13	Bcc173	RAPD01	CACAG	4	m6A	0.99120104	6759	6819	36.93579
P13	Bcc173	RAPD01	GTWWAC	5	m6A	0.9644013	1788	1854	37.08669
P13	Bcc173	RAPD01	BNSGTCGACS	8	m6A	0.49530563	4959	10012	38.625328
P13	Bcc173	RAPD01	BVNNGTCGACNNS	9	m6A	0.40537727	4674	11530	38.828842
P13	Bcc174	RAPD01	GCAGNNNNNNCTC	3	m6A	0.97778547	2817	2881	36.797657
P13	Bcc174	RAPD01	GAGNNNNNNCTGC	2	m6A	0.9673724	2787	2881	37.001793
P13	Bcc174	RAPD01	CACAG	4	m6A	0.9672645	6796	7026	37.008827
P13	Bcc174	RAPD01	GTWWAC	5	m6A	0.92661035	1755	1894	36.71909
P13	Bcc174	RAPD01	BNSGTCGACV	8	m6A	0.5303721	6400	12067	38.93094
P13	Bcc174	RAPD01	ANNGTCGACSNS	8	m6A	0.2777086	446	1606	39.278027
P13	Bcc174	RAPD01	TGTCGACSNV	6	m6A	0.25835544	487	1885	40.41684
P13	Bcc174	RAPD01	HGGTCGACT	7	m6A	0.15774648	56	355	40.535713
P13	Bcc179	RAPD01	GCAGNNNNNNCTC	3	m6A	0.9654005	2567	2659	31.196728
P13	Bcc179	RAPD01	GAGNNNNNNCTGC	2	m6A	0.94170743	2504	2659	31.473642
P13	Bcc179	RAPD01	CACAG	4	m6A	0.9647059	6150	6375	31.726667
P13	Bcc179	RAPD01	GTWWAC	5	m6A	0.9242762	1660	1796	31.465061
P16	Bcc201	RAPD01	GCAGNNNNNNCTC	3	m6A	0.95828503	2481	2589	30.884321
P16	Bcc201	RAPD01	GAGNNNNNNCTGC	2	m6A	0.9389726	2431	2589	30.964212
P16	Bcc201	RAPD01	CACAG	4	m6A	0.9560849	5900	6171	30.93983
P16	Bcc201	RAPD01	GTWWAC	5	m6A	0.890866	1502	1686	31.267643
P16	Bcc201	RAPD01	BNSGTCGACV	8	m6A	0.50343186	5281	10490	33.71918
P16	Bcc201	RAPD01	CCCGGG	2	m4C	0.41577625	1323	3182	34.860924
P16	Bcc201	RAPD01	ANNGTCGACSNS	8	m6A	0.28057554	390	1390	35.25641
P16	Bcc201	RAPD01	WGTCGACSNS	6	m6A	0.25921908	478	1844	34.843098
P16	Bcc201	RAPD01	HGGTCGACT	7	m6A	0.18688525	57	305	35.245613
Ref	J2315	RAPD02	CACAG	4	m6A	0.9907407	6741	6804	44.689957
Ref	J2315	RAPD02	GTWWAC	5	m6A	0.9514811	1863	1958	44.31884

Table S6. Pan-genome analsis of the *B. cenocepacia* reference genomes and RAPD-specific genomes using a minimum cutoff of 95% BLASTP identity. Distribution of genes after collapsing paralogous clusters (“Homologs”) are shown, with “Reference genomes” to included only PacBio and NCBI assemblies.

Column	Description
Reference genomes	Homologs identified by Roary for PacBio assemblies and NCBI reference genomes (J2315, HI2424, AU1054 and H111).
RAPD01	Homologs identified by Roary for RAPD01 genome assemblies
RAPD02	Homologs identified by Roary for RAPD02 genome assemblies
RAPD04	Homologs identified by Roary for RAPD04 genome assemblies
RAPD06	Homologs identified by Roary for RAPD06 genome assemblies
RAPD09	Homologs identified by Roary for RAPD09 genome assemblies
RAPD15	Homologs identified by Roary for RAPD015 genome assemblies
RAPD44	Homologs identified by Roary for RAPD044 genome assemblies

Table S6. Pan-genome analsis of the *B. cenocepacia* reference genomes and RAPD-specific genomes using a minimum cutoff of 95% BLASTP identity. Distribution of genes after collapsing paralogous clusters ("Homologs") are shown, with "Reference genomes" to included only PacBio and NCBI assemblies.

	Reference genomes	RAPD01	RAPD02	RAPD04	RAPD06	RAPD09	RAPD15	RAPD44
Core genes (99% <= strains <= 100%)	3666	4723	4097	3734	3844	4095	6511	5816
"Soft-core" genes (95% <= strains < 99%)	0	1576	2034	2852	2637	0	0	0
"Shell" genes (15% <= strains < 95%)	5171	1037	1172	1332	626	2705	33	856
"Cloud" genes (0% <= strains < 15%)	5138	342	2248	1275	872	127	0	104
Total number of genes	13975	7678	9551	9193	7979	6927	6544	6776
