# Supplementary material for: Phenotypic diversity and genotypic flexibility of Burkholderia cenocepacia during long-term chronic infection of cystic fibrosis lungs
Source: Genome Res. 2017 Apr;27(4):650–62. doi: 10.1101/gr.213363.116 (PMC5378182; doi:10.1101/gr.213363.116)
Supplement: Supplemental Material [file supp_gr.213363.116_Supplemental_Methods_Text_Figures.docx]

**Supplemental Methods**

*DNA Extraction and Genome Sequencing*

For Illumina sequencing, genomic DNA was extracted from 1 ml of overnight culture of each *B. cenocepacia* isolate using the Puregene Gentra Archival Bacterial DNA extraction kit (Qiagen), as per manufacturer’s protocol for Gram-negative bacteria. Multiplexed sequencing libraries were made using the Illumina Nextera XT DNA Sample Prep kit (Illumina) following the manufacturers’ instructions. Sequencing was performed with single-end and paired-end reads at the Faculty of Pharmaceutical Sciences Sequencing Centre (University of British Columbia) to a minimum read depth of 60 X. Raw sequence reads are deposited on the NCBI Sequence Read Archive with the accession number SRP PRJNA289138.

For PacBio sequencing, genomic DNA was extracted from 10 ml of overnight cultures using the Puregene Gentra Archival Bacterial DNA extraction kit to yield at least 20 μg of DNA. PacBio sequencing was performed using two SMRTcells per isolate with P4-C2 chemistry.

*Clinical isolate collection*

Clinical data for all patients in this study were previously collected, as approved by the UBC Research Ethics Boards (H07-01396) (Zlosnik et al. 2011a).

To minimize passaging of the clinical isolates, a single passage was used to generate new stocks from the originals, and all subsequent phenotypic assays were a single passage away from these stocks.

*Phenotypic Analyses*

*Biofilm Assay.* We tested the ability of *B. cenocepacia* strains to form biofilm by adhering to 96-well polypropylene microtitre dishes following a previously established protocol (O'Toole and Kolter 1998; Conway et al. 2002). Bacteria were grown in ½ strength LB at 37 °C for one overnight, washed with 10 mM MgSO_4_, and inoculated into synthetic cystic fibrosis medium (SCFM) (Palmer et al. 2007) at OD_600_=0.1. Microtitre plates were incubated without shaking at 37 °C for 48 hours. The plates were washed to remove planktonic cells, fixed, and stained for 15 min with 125 μl of 1% (wt./vol.) crystal violet. After washing the plates to remove residual crystal violet, plates were dried, followed by solubilizing of the crystal violet with 200 μl of 95% (vol./vol.) ethanol. The final biofilm staining was measured at an absorbance wavelength of 595 nm. Experiments were performed at least three independent times, with the mean and standard deviation calculated.

*Mucoid Phenotype Classification*. To assess mucoidy in these isolates, we followed a previously established protocol (Zlosnik and Speert 2010; Zlosnik et al. 2011a; Zlosnik et al. 2015) that showed that various *B. cenocepacia* strains develop elaborate exopolysaccharide on the yeast extract mannitol media (YEM; 0.5 g of yeast extract liter^−1^ and 4 g of mannitol liter^−1^ supplemented with 20 g of agar liter^−1^). We streaked out fresh *B. cenocepacia* isolates onto YEM plates and incubated at 37°C for 48 h. Following the published scoring method, we defined mucoidy as a binary characteristic, with non-mucoid isolates given a score of zero and mucoid isolates given a score of one.

*Motility Assay.* The swimming motility for each isolate was individually measured using the motility agar (0.3% LB agar plates) as previously described (Zlosnik et al. 2015). Plates were poured to 3 mm thickness on the bench and used within the same day. Freshly grown overnight culture for each isolate was normalized to OD_600_=0.6 and 2 μl of the normalized bacterial culture was inoculated into the center of the motility agar. Motility plates were then incubated for 48 hours at 37°C and kept upright. At 48 hours, we measured the diameter of the motile zone for each isolate. We used *P. aeruginos*a PAK and *P. aeruginosa* PAK *ΔfliC* (Feldman et al. 1998) as motile and nonmotile controls, respectively.

*G. mellonella Killing Assay.* Larvae were purchased from the Cherry Creek Cricket and Worm Farm (British Columbia, Canada), sorted into petri dishes upon arrival and allowed to acclimate to the laboratory for at least one overnight. Fresh overnight cultures of *B. cenocepacia* isolates grown in ½ strength LB, pelleted and washed with 10 mM MgSO_4_. Each larva was injected with 10 μl of 10^6^ or 10^7^ cfu/ml (equivalent of 10^4^ or 10^5^ cfu respectively) of bacteria plus 1.2 mg/ml of Ampicillin to the hindmost left proleg using the BD Ultra-Fine II Insulin Syringe (Van Leene et al.). For each isolate, ~ 60 larvae were injected at each concentration. Each experiment included the negative control larvae, which were injected with 10 μl of 10 mM MgSO_4_ plus 1.2 mg/ml ampicillin to measure any lethal effects due to the injection process. In addition, each experiment also included two control *B. cenocepacia* isolates, a high-killing strain (Bcc137) and a low-killing strain (Bcc183) to allow for comparison of virulence across different experimental weeks.

After injection, larvae were placed in the dark at 37 °C and the live versus dead larvae were monitored every 24 h postinfection for three days. Larvae were considered dead when they showed no response to touch or displayed no obvious movement. Data analyses were done using Kaplan-Meier Survival Analysis (R version 3.2.2 and survival package 2.38) and we calculated the r-means of larvae survival infiltrated with different *B. cenocepacia* isolates to measure virulence.

*In vitro Growth Assays in Luria broth (LB) and synthetic cystic fibrosis medium* (*SCFM). B. cenocepacia* isolates were grown in ½ strength LB at 37 °C for one overnight, washed with 10 mM MgSO_4_, and inoculated into either ½ LB or SCFM (Palmer et al. 2007) at OD_600_ = 0.1, in clear, flat-bottom 96-well microtitre plates. Microtitre plates were incubated, shaking at 37 °C for ~24 hours in microtitre plate reader Safire2 (Tecan-US, Durham, NC, USA). The Tecan Safire2 plate reader measured OD every 15 minutes at OD_600_ nm. We used the logistic model to analyze the growth data using the R package growthcurve (<https://github.com/briandconnelly/growthcurve>) and generate maximum growth, and maximum growth rate for each isolate.

*Statistical Analyses*

All phenotypic data were analyzed in R (R Core Team 2016). For each phenotype, the raw data were averaged across replicates and normalized to the maximum measurement for that particular phenotype measured across 215 *B. cenocepacia* isolates. For instance, the maximum raw value for motility corresponds to 90mm, corresponding to the maximum size of the agar plate. To compare phenotypic variations for all phenotypes except mucoidy of isolates within each patient as a function of time, we applied a linear model (lm) with the phenotypic variables as dependent and time as independent variables (formula: lm(phenotype ~ time of isolation in days since the first *Bcc* isolate)). For the mucoid phenotype, we used logistic regression. We also tested whether the RAPD genotype can function as an interaction term in the linear model (formula: lm(phenotype ~ time of isolation in days since the first *Bcc* isolate * RAPD genotype)). We created correlation matrices by calculating the spearman’s rank correlation coefficient, corrected for multiple testing and plotted using the Hmisc and corrplot R packages. To test whether isolates from the same isolation date within each patient showed significant differences in biofilm, we performed one-way ANOVA extracted the statistical summary using the broom package. Most figures were created using the ggplot2 package in R (R Core Team 2016).

De novo *Genome Assembly*

We assembled *B. cenocepacia* genomes from Illumina reads using a custom assembly pipeline (<https://github.com/rehrlich/ray_assembly>): trimming the Nextera adaptor sequences using Trimmomatic-v0.30 (Bolger et al. 2014); joining the overlapping reads using COPE-v1.1.2 (Liu et al. 2012); correcting sequence errors with ALLPATHS-LG stand-alone ErrorCorrectReads.pl utility (Butler et al. 2008); and the de novo assembly of the corrected reads were done using the Ray-v2.2.0 assembler (Boisvert et al. 2012). The performance of Ray assembler compared to other assemblers has been previously assessed (Bradnam et al. 2013; Powers et al. 2013; Koren et al. 2014).

For PacBio-sequenced isolates (Bcc001, Bcc022, Bcc023, Bcc030, Bcc116, Bcc129, Bcc137, Bcc173, Bcc174, Bcc179, Bcc201 and J2315), the PacBio reads were assembled with the HGAP assembler, and the consensus sequence polishing by the Quiver algorithm (Chin et al. 2013) using the SMRT Analysis Suite (Pacific Biosciences). Given that bacterial chromosomes and plasmids are typically circular, we used Circlator, a post-assembly tool for circular closure (Hunt et al. 2015). Circlator detected circular sequences by assembling PacBio corrected reads that mapped to contig ends, resolved the redundant sequences shared by the overlapping ends, removed all contigs that failed to circularize and were completely contained within the sequence of another contig, and permuted the start position of each circularized contig to a user-defined gene (or by default *dnaA*) (Hunt et al. 2015). For a multi-chromosome bacterial species like *B. cenocepacia*, we could not rely on the canonical *recA* or *dnaA* genes as landmarks for circularizing because they are only found on chromosome one. Instead, we used the DNA-partitioning systems, *parABS*, to permute the start of each circular replicon because *ParAB* paralogs are present on each of the chromosomes and plasmid (Dubarry et al. 2006). Base modification analysis was performed with the SMRT Analysis Suite using standard mapping protocols. All assembled genomes were annotated with the rapid prokaryotic genome annotation pipeline, Prokka, v1.12 (Seemann 2014).

Whole genome analysis of both Illumina and PacBio assemblies were performed using pyani (<https://github.com/widdowquinn/pyani/>) to calculate the pair-wise average nucleotide identity (ANI) (Konstantinidis and Tiedje 2005; Goris et al. 2007; Richter and Rossello-Mora 2009) by aligning all the input sequences with NUCmer from MUMmer (Kurtz et al. 2004) (ANIm) and the tetranucleotide frequencies. We performed whole genome sequence alignment using both progressiveMauve (Darling et al. 2010) and used the Bioconductor package DECIPHER v2.1.1 to create synteny dot plots (Wright 2015).

*Pan-genome Analyses*

Pan-genome analyses for all isolates with <500 contigs or for each RAPD genotype were performed using the rapid large-scale prokaryote pan-genome analysis pipeline, Roary v 3.4.2, from the Sanger Institute (<https://github.com/sanger-pathogens/Roary>; (Page et al. 2015)). We included 206 genome sequences in the large pan-genome analysis: all PacBio assemblies, any Illumina assemblies with <500 contigs, and 4 NCBI reference genomes. Our input files were the GFF3 files from the Prokka-annotated assemblies above (Seemann 2014). We also include the Prokka-annotated NCBwI reference genomes *B. cenocepacia* HI2424 (NCBI Taxonomy ID*:* 331272) and AU1054 (NCBI Taxonomy ID*:* 331271). To create core-genome alignments, Roary first clustered coding regions using CD-HIT (Fu et al. 2012), followed by an all-against-all comparison between the coding regions using a minimum cutoff of 75% BLASTP identity (unless otherwise specified in the legends), ignoring truncated and un-annotated genes, and genes with 5% Ns and genes with fewer than 120 bases. By combining the BLAST results with MCL clustering (Enright et al. 2002), Roary identified the homologous groups of genes, with a codon-aware multiple sequence alignment generated by PRANK (Loytynoja 2014) for both the core genome and the accessory genome.

*Estimating Population Structure and Phylogeny*

Core-genome SNPs were extracted from the Roary core genome alignment for each RAPD genotype or for all 215 isolates using the SNP Sites Program v 2.0.2 from the Sanger Institute (<https://github.com/sanger-pathogens/snp_sites>). These SNPs were used as input for maximum likelihood inference with RAxML v 8.2.0 (Stamatakis 2014). To create the best-scoring maximum-likelihood tree with bootstrapping support, we used the GTRCAT model with the –f a and the –N autoMRE options in RAxML (Stamatakis 2014). To map the phenotypic data onto the maximum-likelihood tree, we used the plotTree.R script (<https://github.com/katholt/plotTree>; (Inouye et al. 2014)).

*Variant Calling and Genome Loss*

We first aligned Illumina reads for all isolates to the NCBI J2315 reference genome (Holden et al. 2009) (NCBI Taxonomy ID*:* 216591; NCBI genome accessions: NC_011000.1, NC_011001.1, NC_011002.1, NC_011003.1) using the short read aligner BWA v 0.7.9a (Li and Durbin 2009). Single-nucleotide variants were identified and filtered with the SAMtools toolbox v 1.1. (Li et al. 2009). Each variant was annotated with a custom Perl script and the gene information for J2315 reference downloaded from NCBI. However, due to the difficulty of obtaining high quality alignments in poorly conserved regions for any RAPD genotypes except for RAPD02, we repeated the alignment and variant calling procedure using our PacBio reference genomes for each respective RAPD genotype. For isolates of the RAPD01, RAPD04, RAPD06, RAPD09, RAPD15 or RAPD44 genotypes, we used the PacBio assembly for Bcc173, Bcc023, Bcc001, Bcc116, Bcc137, or Bcc129 as reference genome, respectively. We kept any variant locations with at least 80% of the mapped reads agreeing with the variant call and a root mean square mapping quality of at least 30.

Using a previously established approach, we used variation in read coverage to identify regions of genome loss (Alkan et al. 2011; Thompson et al. 2013). We mapped the Illumina reads for each isolate to its respective PacBio RAPD reference assembly, counted the aligned read pairs per CDS window, and normalized to the total number of read pairs in each strain. For each strain, we identified regions where the mapped reads were significantly lower after normalizing for RAPD-specific mapping variation as regions of genomic loss. Furthermore, for each CDS and each isolate, we recorded the number of bases covered by reads relative to the number of bases covered for the corresponding reference isolate; a gene was considered mutated relative to the reference when that ratio was below 90%.

**Supplemental Results**

**S1 Text: Survey of six clinically relevant phenotypes for the 215 *B. cenocepacia* clinical isolates collected from 16 patients**

*Motility:* Swimming motility of all 215 isolates was assayed by motility zone assay as a proxy for the presence of functional flagella (Mahenthiralingam et al. 1994; Zlosnik et al. 2014). Clinical *Bcc* isolates have been shown to use flagella to invade epithelial cells, which also stimulate host inflammation (Tomich et al. 2002; Urban et al. 2004). Previous studies have observed loss of motility during long-term persistence by *B. cenocepacia*, as well as other bacteria including *P. aeruginosa* (Smith et al. 2006; Lieberman et al. 2011; Folkesson et al. 2012; Lieberman et al. 2014). The isolate collection showed substantial variation in swarming, with the motile zone range varying ~45-fold, from 2 to 90 mm (**Supplemental Table S2**). Longitudinal series showed significant motility reductions over time in 6 of 16 series, with a mean decrease in these series of 6.6% ± 4.4% per year (**Supplemental Fig. S2A** and **Supplemental Table S1D**). No obvious connection was seen between declining motility and RAPD genotype (although 2 of 4 RAPD04 series showed similar declines), nor was declining motility correlated with high motility in the earliest isolate of each series. However, RAPD type on its own was a significant predictor of motility, RAPD01 and RAPD02 were mostly non-motile, and RAPDs 06, 15, and 44 were mostly motile (p << 0.001 and adjusted R^2^ = 36% in linear regression).

*Biofilm formation:* Mature *B. cenocepacia* biofilms act as a barrier to antibiotics, help evade immune detection by neutrophils, and contribute to persistence (Van Acker et al. 2013; Murphy and Caraher 2015). Biofilm formation of all 215 isolates was assays by crystal violet staining after 48 hr incubation of static cultures grown in 96-well polypropylene microtitre plates; higher levels of stain detected by optical density (OD) indicate increased biofilm formation. Values varied considerable, from OD_595nm_ = 0.006 to 0.523 (**Supplemental Table S2**). Using linear regression, we identified significant changes in biofilm formation over time for 5 of 16 patients, but the direction of change was not consistent. Two series started with low biofilm values that increased over time; in particular the RAPD02 isolates from patient P12 showed dramatic increases in biofilm formation. In contrast, three series showed ~5 – 8% declines per year in isolates’ capacity to form biofilms (**Supplemental Fig. S2B**; **Supplemental Table S1E**). RAPD type was also a significant predictor of biofilm formation (p << 0.001 and adjusted R^2^ = 49.9%) with RAPD06 forming particularly robust biofilms overall.

*Virulence in an insect model:* A well-established acute virulence assay (killing of *G. mellonella* moth larvae) was used to assess the capacity of each isolate to cause acute infections(Seed and Dennis 2008; Uehlinger et al. 2009). Although an imperfect model, similarities exist between the insect and mammalian innate immune systems (Buchmann 2014). Furthermore, differences among *Burkholderia* isolates’ ability to kill *G. mellonella* larvae is comparable to a rat agar bead infection model, and can be performed at much higher throughput compared to rodent models (Seed and Dennis 2008; Uehlinger et al. 2009). For each of the 215 *B. cenocepacia* isolates, 60 larvae were injected with two distinct inoculums (10^4^ cfu or 10^5^ cfu per larvae, corresponding to virulence_low and virulence_high respectively). Larval survival was determined at 24 and 48 hours after inoculation, and the *r* mean values from Kaplan-Meier survival analysis were calculated (see Methods). Isolate killing varied widely, ranging from all larvae killed within 24 hours (normalized value of 1) to all larvae surviving after 48 hours (normalized value of 0). Two series (P09 and P03) decreased in acute virulence over time, but each was only significant at one of the dosages. Subgroup B isolates from patient P09 had among the highest acute virulence score, despite the relative stable and healthy lung function of P09. Significant decreases in acute virulence were seen for the P09 series at the low dose, whereas the P03 series showed a decrease in virulence over time only for the high inoculation (**Supplemental Tables S1F** and **S1G, Supplemental Fig. S2C**). RAPD genotype was a significant predictor of acute virulence in the insect model at both inoculation concentrations (p << 0.001, R^2^ = 0.270 and 0.177 for low and high doses respectively) with RAPDs 04, 15 and 44 being particularly virulent in the insect model.

*Mucoidy:* Previous studies of *B. multivorans*, and *B. cenocepacia* reported that strains convert from a mucoid to a non-mucoid colony phenotype during chronic infection (Zlosnik and Speert 2010; Silva et al. 2011; Zlosnik et al. 2011b). Furthermore, many B. cenocepacia isolates are non-mucoid (Zlosnik and Speert 2010; Zlosnik et al. 2011a; Zlosnik et al. 2015). We performed a qualitative assessment of the mucoid morphotype on yeast extract mannitol agar (categorically assigning each strain as either mucoid or non-mucoid), finding variation in mucoidy in only 6 of 16 patient series; all RAPD02 isolates were non-mucoid as previously reported (Zlosnik and Speert 2010; Zlosnik et al. 2011a; Zlosnik et al. 2015). Regression analysis identified no longitudinal series with significant changes in mucoidy over time (p > 0.20 for all 6 series with mucoidy variation, **Supplemental Table S1H**), in contrast to other reports. However, RAPD type was a significant predictor of mucoidy status (p << 0.001 and adjusted R^2^ = 0.463) with all subgroup B isolates being mucoid and RAPD06 having high incidence of mucoidy.

*Growth in liquid media:* To systematically characterize the growth dynamics of *B. cenocepacia*, we used microtitre plate readers to monitor changes in optical density (OD) of bacterial cell suspension over 24 hours, in 0.5x LB and SCFM. SCFM has been shown to nutritionally mimic the cystic fibrosis sputum, and induces similar gene expression and quorum-sensing signaling as *P. aeruginosa* grown in sputum (Palmer et al. 2007). We modeled the resulting cell growth-curves against a logistic curve and extracted the maximum yield (final OD) and growth rate (OD/second) for each isolate (**Supplemental Figure S3)**. Using linear regression, we found that four out of five patient series showed significant decline in LB growth rate over isolation time, ranging from -1.7% to -7.2% (p < 0.03). There were no significant changes observed for the maximum yield in LB for any patient series (**Supplemental Figure S3** and **Table S1I)**. We only saw two patient series, both from RAPD04, showing a significant decline of ~4% in SCFM growth rate (p<0.03) and maximum yield ranging from -0.9% to -3% over isolation time (p<0.05). Together, our growth data indicate potential metabolic changes in the later isolates compared to the earlier isolates.

**S2 Text: Control sequencing, assemblies and whole genome analyses**

For the J2315 control strain, we generated four contigs, corresponding to the four replicons found in the reference sequence. One major difference between our PacBio assembly and the NCBI reference sequence is that the PacBio assembly did not resolve 57-kb duplicated regions found at two different locations on chromosome 1 (highlighted in **Fig. S5** by a box; (Holden et al. 2009). This is mostly likely a consequence of the duplicated regions being longer than the typical PacBio insert sizes. There were also ten single nucleotide discrepancies between our PacBio assembly and the NCBI J2315 reference genome (PacBio assembly statistics and PacBio and Illumina comparisons in **Supplemental Table S3B** and **S3C**). We compared the PacBio and Illumina assemblies by aligning the respective assemblies using Mauve, and extracted total number of SNPs using the “Extract SNPs” function (**Supplemental Table S3B**). We observed that on average, ~65% of the SNP counts using the Mauve “Extract SNPs” function were located near the ends of Illumina contigs, potentially as a result of contig breaks due to repetitive sequences (**Supplemental Table S3B**). The Illumina assemblies were used to extract MLST loci for each isolate (**Table S4**, **Methods**).

To gain genomic insights into our collection of *B. cenocepacia* isolates, we calculated pair-wise average nucleotide identity (ANI) and tetranucleotid frequencies between the two NCBI reference genomes for *recA* subgroup A (J2315), *recA* subgroup B (HI2424), all the PacBio and Illumina assemblies (**Fig. S6** and **Supplemental File S8**). Traditionally, bacterial species were defined based on DNA-DNA hybridization (DDH) techniques, with 70% as the species definition. ANI is an *in silico* DDH analysis, with ~94% ANI corresponding to the species boundary. To calculate pair-wise ANI for all *B. cenocepacia* genomes, we used *pyani* which implements MUMmer to align fpairs of genomes, identify matching regions and calculate the average percent nucleotide identity for all matched regions (Konstantinidis and Tiedje 2005; Goris et al. 2007; Richter and Rossello-Mora 2009). We observed that all the isolates within the *recA* subgroup A or B had ~99% ANI (**Fig. S6A** and **Supplemental File S8**). In contrast, comparison between *recA* subgroup A and B isolates showed ~95% ANI, which corresponded to the long branch length that we observed between subgroup A and B isolates (**Fig. 4** and **Fig. S8**). We did not observe significant differences in tetranucleotide frequencies between different isolates (**Supplemental File S9**).

To identify breakpoints in chromosomal synteny between the different RAPD types, we used DECIPHER (Wright 2015) to create synteny dot plots of all PacBio assemblies and two NCBI references (**Fig. S6B** and **C**). In general, we observed synteny between isolates from the same RAPD genotype with the exception of RAPD01 isolates (Bcc173, Bcc174, Bcc179 and Bcc201).

PacBio single molecule sequencing can distinguish methylated bases (particularly 6-me-A) from unmodified bases and as a consequence one can detect genome-specific methylation motifs (Zak et al. 2012; Eutsey et al. 2015). DNA methylation in bacterial genomes serves diverse roles; acting in restriction modification (RM) systems to either protect the host genome against phage and other foreign DNA (Mruk and Kobayashi 2014) or to promote horizontal gene transfer (Eutsey et al. 2015). DNA methylation status also affects transcription, DNA repair, gene expression, cell cycle control, phase variation and pathogenesis (Zweiger et al. 1994; Marinus and Casadesus 2009; Srikhanta et al. 2010; Fang et al. 2012). In J2315, our control reference genome, we identified two methylation motifs (5’-CAC**A**G-3’ and 5’-GTWW**A**C-3’), which were found in all 12 PacBio-sequenced isolates regardless of RAPD types. Beyond these two common motifs, each RAPD genotype also had distinct methylation motifs, suggesting the presence of RAPD-specific RM systems (**Table S5**). In addition, changes were observed in the methylation motifs among serial isolates from the same patient. Specifically, comparing early isolates (Bcc173 and Bcc174) to an isolate (Bcc179) from 8 years later, we observed that two similar methylated motifs (5’-BNSGTCG**A**CS-3’ and 5’-BVNNGTCG**A**CNNS-3’) were lost during long-term persistence (**Table S5**). The functional impact, if any, of this DNA modification is, at present, unknown.

**S3 Text: Pan-genome analyses identified clade-specific gene content and potential inter-patient transmission**

To identify orthologs, the Roary algorithm filters and pre-clusters the coding sequences, performs an all-against-all BLASTP comparison and a final clustering with MCL. Preliminary tests showed the overall distribution of genes was relatively insensitive to the BLASTP identity threshold; we therefore chose a 75% cutoff, as it appeared closest to an inflection point (Hogg et al. 2007; Boissy et al. 2011) (**Fig. S8**). Since Roary ignores genes that are truncated, un-annotated (for example at contig breakpoints) or shorter than 120 bases, certain genes may be systematically lost from the analysis. We performed the pan-genome analysis with only the subset of reference-quality PacBio assemblies and NCBI assemblies. As predicted, based on the smaller number of genomes and the inclusion of more complete genome assemblies, this analysis found fewer rare genes (**Fig. S9** and **Table 4**).

Analysis of the Roary gene presence matrix with the core gene phylogeny (built from orthologous clusters only) showed that large sets of accessory genes were segregated according to their respective RAPD type (**Fig. 4B**). To isolate RAPD-specific genes, we identified homologous clusters that were exclusive to a RAPD type and common to all isolates within that RAPD type (**Supplemental File S11**). For instance, the cytochrome-*c* maturation (*ccm*) operon (which functions to export cytochrome *c* to the membrane followed by covalent attachment of heme (Schulz et al. 1999) was found exclusively and in all RAPD01 isolates. Consistent with previous studies, we confirmed that only the RAPD02 strains encode the cable *cbl* pilin (having 30% amino acid identity to the *E. coli* CFA/I fimbriae), which plays a critical role in the transmission and *in vivo* persistence of the lineage (Sajjan et al. 1995; Sajjan et al. 2000; Goldberg et al. 2011). One RAPD04-exclusive gene was the mRNA interferase HigB gene, part of a toxin-antitoxin system that regulates the amino-acid starvation response, multidrug resistance and virulence expression (Hurley and Woychik 2009; Christensen-Dalsgaard et al. 2010). Finally, only the RAPD06 isolates encoded a strain-specific nitric oxide response regulator NorR2 and tellurite resistance proteins.

Using core gene orthologs identified by Roary, we built phylogenetic trees for each RAPD type. We found that isolates from RAPD01 and RAPD02 formed patient-specific clades, while RAPD04 and RAPD06 displayed intermingling of clonal lineages among patients. The observed intermingling of clonal lineages suggests either: (1) patients were independently infected by closely related strains; or (2) patients with closely related strains infected each other, indicative of possible transmission.

To differentiate between these two hypotheses, we first investigated the hospitalization records for Patient 2 & 3, as well as Patient 6 and 10. For both of these patients, we did not observe overlapping hospital stays or clinical visits. However, these patients were most likely infected before the strict infection control was put in place in 1994, thus the patients could still have interacted in the community outside of hospital visits. In addition, we mapped on sample isolation dates to the phylogeny (**Fig. S11** and **S12**). These figures illustrate that the branching pattern in the tree and the sampling dates are poorly correlated. Particularly, deeper branching lineages and those branches with shorter distances to the root were not necessarily isolated at earlier time points than others. Instead, in many cases, we observed an inverse pattern between isolation time and branch length. One potential explanation for this observation is that multiple closely related strains co-exist in the patient at any give time point, and this diversity is not captured by the single-isolate sampling strategies utilized in this historical collection.

**S4 Text: Genotype-phenotype association analysis**

To build the genotype matrix used for the genotype-phenotype association analysis, we defined “mutant” (0) as any allele affected by deletions, nonsense, or missense changes relative to the PacBio RAPD reference genome from the earliest available time point; whereas “wild-type” alleles were those matching the reference or with only silent substitutions. This was done by aligning short paired-end Illumina reads to the reference using BWA and calling single-nucleotide changes using samtools and deletions by coverage analysis. This approach was used to minimize “false negative” gene absence calls from our Roary pan-genome analysis due to lack of annotation at contig breakpoints.

We observed that *dnaK* mutations are highly correlated (ρ = 0.57, **Fig. 7B**) with the loss of motility in our *Burkholderia* isolates. Another candidate, *cheA* encodes a histidine kinase that phosphorylates the downstream response regulator CheY, which regulates flagellar motors (Hansen et al. 2008). The *aer* gene encodes a receptor that senses environmental oxygen (via its PAS-domain), which regulates CheA and flagellar motility (Taylor et al. 1999). CadA, a lysine decarboxylase, has been shown to negatively regulate the adherence of enterohemorrhagic *E. coli* to host cells, potentially by modulating expression of flagellar and fimbriae components (Vazquez-Juarez et al. 2008). Interestingly, we found the quorum-sensing sensor kinase gene *qseC* associated with motility (ρ = 0.33) and biofilm (ρ = 0.39). QseC has been shown to regulate motility and biofilm formation in *Escherichia coli* (Yang et al. 2014) and is found in at least 25 human and plant pathogens.

Our genotype-phenotype associations aggregated all coding changes from the reference as “mutant”, and therefore does not account for how different mutations at the *dnaK, papC, gcvA* and *qseC* loci might affect biofilm and motility. However, we lack the statistical power to detect SNP-specific correlation. Therefore, we used the Roary gene possession matrix to ask whether gene presence or absence alone at these four loci could explain the phenotypic variations that we observed in biofilm and motility. With the exception of *qseC*, gene loss in *dnaK, papC,* or *gcvA* was correlated with less motility (**Fig. S14**) and less biofilm formation (**Fig. S15**).

We observed that there is a positive correlation between biofilm formation and motility in sub-group A but a negative correlation in the sub-group B lineage (**Fig. 3**). While we lack statistical power to dissect how genetic background differences between sub-group A and B may contribute to this phenotypic difference, we analyzed gene presence or absence of these four loci. Interestingly, we identified three *gcvA* paralogs (gcvA_18; group_23721; group_24122) that are missing in all subgroup B isolates (RAPD44 and RAPD15), but present in all subgroup A isolates.

**Supplemental Figures**

**Figure S1. *B. cenocepacia* isolates sample collection and the associated patient lung function (%FEV_1_). A.** Each row represents all the isolates from the patient (P01 to P16) corresponding to the sampling time for each of the isolate. Colors indicate the RAPD genotype these isolates belong to. **B.** Patient lung functions were assessed by %FEV_1_ (FEV1) and data were collected from the patient charts. Colors indicate the RAPD genotypes of the *B. cenocepacia* isolated from that particular patient.

**Figure S2. Scatterplots of *B. cenocepacia* phenotypes versus time of isolation.** Normalized phenotype data for **A.** motility; **B.** biofilm; and **C.** virulence for each isolate were plotted against the number of days since the first isolate was collected for that particular patient. Colors indicate the RAPD genotypes of the *B. cenocepacia*.

**Figure S3. Scatterplots of maximum *B. cenocepacia in vitro* growth and growth rate versus time of isolation.** Maximum growth and growth rate in ½ LB (**A** and **B**) and SCFM (**C** and **D**) for each isolate were plotted against the number of days since the first isolate was collected for that particular patient. Colors indicate the RAPD genotypes of the *B. cenocepacia*.

**Figure S4. Strip charts of *B. cenocepacia* phenotypic variations for each RAPD type.** Normalized phenotype data for **A.** motility; **B.** biofilm; **C.** mucoidy; **D.** virulence at low inoculum; **E.** virulence at high inoculum; **F.** lung function (%FEV_1_); **G.** maximum growth rate in SCFM and **H.** maximum growth rate in half LB for each isolate were plotted against the RAPD type.

**Figure S5. Mauve whole genome alignment of NCBI reference genome for *B. cenocepacia* J2315 and PacBio-assembled J2315.** Each colored region is a locally collinear block where there is no observed rearrangement of the backbone sequence. The top colored blocks are for NCBI reference genome and the bottom colored blocks are the PacBio re-sequenced and assembled J2315 genome from this study, with three chromosomes and plasmid are indicated. Black box indicates the “missing” duplicated region in the PacBio assembly.

**Figure S6. Whole genome analyses of PacBio assemblies and NCBI reference genomes: A.** Percent average nucleotide identity calculated using MUMmer algorithm; **B.** synteny dot plot for *recA* subgroup A genomes and **C.** synteny dot plot for *recA* subgroup B genomes.

**Figure S7. Mauve whole genome alignments of PacBio assembly of Bcc129 against NCBI reference genome HI2424 revealed chromosome fusion. A.** Mauve whole genome alignment. The top colored blocks are for NCBI reference genome and the bottom colored blocks are the PacBio Bcc129 genome from this study. Boxes E and G are regions that are contiguous in Bcc129, but belonged to different chromosomes in HI2424. Boxes F and H are contiguous regions between BccHI2424 and Bcc129. **B.** PCR amplification with primers designed to spanned the highlighted regions E to H with the following expected band size: E = 5478bp, F = 5390bp, G = 5408bp, and H = 5045bp.

**Figure S8. Number of gene clusters as identified by Roary versus % BlastP identity threshold for A.** orthologs; and **B.** homologs in all *B. cenocepacia* genomes. Colors indicate whether the gene clusters are: core, soft core, shell or cloud genes.

**Figure S9. Core genome single nucleotide polymorphism (SNP) phylogeny and gene-presence-absence matrix of PacBio-sequenced *B. cenocepacia* isolates and four NCBI reference genomes J2315, MC03, AU1054, HI2424.** **A.** We aligned the core genomes of twelve PacBio-sequenced isolates (Bcc001, Bcc022, Bcc023, Bcc030, Bcc116, Bcc129, Bcc137, Bcc173, Bcc174, Bcc179 and J2315) with MC03, AU1054 and HI2424 to identify core genome SNPs and built the unrooted phylogeny using FastTree. The isolates are colored based on their RAPD genotype. The *recA* sub-group A isolates formed a distinct clade from *recA* sub-group B isolates. **B.** Using the pan-genome analysis with Roary, we identified 3645 core genes (present in at least 99% of the strains), 5345 shell genes (present in at least 15% of the strains up to 95% of the strains) and 6706 cloud genes (present in less than 15% of the strains). Mapping the gene presence-absence matrix from the Roary pan-genome analysis onto the unrooted phylogeny from **A**, we can clearly see distinct differences in gene composition that separate *recA* sub-group A strains from *recA* sub-group B strains.

**Figure S10. Phenotypic diversity of *B. cenocepacia* RAPD09, 15 and 44 isolates. A.** We measured the phenotypes and associated patient lung function as described above. To generate the heatmap, data were normalized as described in Methods. The core-genome phylogeny and the associated heatmap of RAPD09 with the scale bar for the heatmap on the top right. **B.** We measured the phenotypes and associated patient lung function as described in Methods. To generate the heatmap, data were normalized as described in Methods. Heatmap of the phenotypes is mapped onto the core-genome phylogeny of *recA* sub-group B strains (RAPD15 and RAPD44) with the scale bar for the heatmap on the top right.

**Figure S11. Isolation date of *B. cenocepacia* RAPD01 and 02 isolates.** The core-genome phylogeny and the associated isolation dates and patient IDs for **A.** RAPD01 and **B.** RAPD02 isolates. Small colored dots are used for indicate time points for which 2-3 isolates were collected.

**Figure S12. Isolation date of *B. cenocepacia* RAPD04 and 06 isolates.** The core-genome phylogeny and the associated isolation dates and patient IDs for **A.** RAPD04 and **B.** RAPD06 isolates. Small colored dots are used for indicate time points for which 2-3 isolates were collected.

**Figure S13. Mauve whole genome alignments of early and late isolate pairs show evidence of genome reduction for patients A.** P01 and **B.** P13**.** Each colored region is a locally collinear block where there is no observed rearrangement of the backbone sequence. The colored blocks on the top represent the PacBio assembled genomes for the early isolate and the bottom colored blocks represent the PacBio assembled genomes for the later isolate. The gaps seen in the early isolates indicate missing sequences in the later isolates.

**Figure S14. Boxplot of genetic variations in candidate gene families versus the observed motility variation.** **A – D.** We generated the genotypic variation matrix by defining the variation for each isolate with respect to its RAPD reference, with 1 = “wild type” and 0 = “mutant” (which includes any allele affected by deletion, nonsense, or missense mutations compared to the RAPD PacBio reference sequence from the earliest time point. **E – H.** We used the Roary gene possession matrix where 1 = gene presence and 0 = gene absence. Width of the boxplots represent sample size.

**Figure S15. Boxplot of genetic variations in candidate gene families versus the observed biofilm variation.** **A – D.** We generated the genotypic variation matrix by defining the variation for each isolate with respect to its RAPD reference, with 1 = “wild type” and 0 = “mutant” (which includes any allele affected by deletion, nonsense, or missense mutations compared to the RAPD PacBio reference sequence from the earliest time point. **E – H.** We used the Roary gene possession matrix where 1 = gene presence and 0 = gene absence. Width of the boxplots represent sample size.

**List of Figures and Tables:**

**Figures**

Figure 1. Overview of data collection

Figure 2. Longitudinal series of *Burkholderia cenocepacia* strains isolated from cystic fibrosis lung infections.

Figure 3. Correlation of *B. cenocepacia* phenotypes and patient lung function.

Figure 4. Core gene phylogeny and gene-possession across *B. cenocepacia* isolates.

Figure 5. Genetic and phenotypic diversification of *B. cenocepacia* longitudinal series.

Figure 6. Recurrent gene loss observed in *B. cenocepacia* longitudinal series.

Figure 7. Genetic association testing identifies candidate gene families for the observed phenotypic variation.

**Tables**

Table 1. Summary of 16 *Burkholderia cenocepacia* longitudinal series

Table 2. Correlation between RAPD type and clinically relevant phenotypes

Table 3. Percent change per year in clinically relevant phenotypes across longitudinal series

Table 4. Pan-genome analysis across the isolate collection

**Supplementary Figures**

Figure S1. Scatter plot of lung function (% FEV_1_) for each patient

Figure S2. Scatter plots of *B. cenocepacia* in vitro phenotypes (motility, biofilm, and virulence) against time of isolation.

Figure S3. Scatter plots of *B. cenocepacia* in vitro growth and growth rate against time of isolation.

Figure S4. Strip charts of *B. cenocepacia* phenotypic variations for each RAPD type.

Figure S5. Mauve whole genome alignment of NCBI reference genome for *B. cenocepacia* J2315 and PacBio-assembled J2315.

Figure S6. Whole genome analyses of PacBio assemblies and NCBI reference genomes

Figure S7. PacBio assembly of Bcc129 revealed chromosome fusion.

Figure S8. Number of gene clusters as identified by Roary versus % BlastP identity threshold.

Figure S9. Core genome phylogeny and gene-presence-absence matrix of PacBio-sequenced B. cenocepacia isolates and four NCBI reference genomes, J2315, MC03, AU1054 and HI2424.

Figure S10. Phenotypic diversity of *B. cenocepacia* RAPD09, 15 and 44 isolates.

Figure S11. Isolation date of *B. cenocepacia* RAPD01 and 02 isolates.

Figure S12. Isolation date of *B. cenocepacia* RAPD04 and 06 isolates.

Figure S13. Mauve whole genome alignments of early and late isolate pairs for patient P01 and P13 show evidence of genome reduction.

Figure S14. Boxplot of genetic variations in candidate gene families versus the observed motility variation.

Figure S15. Boxplot of genetic variations in candidate gene families versus the observed biofilm variation.

**Supplementary Tables**

Table S1. Linear regression of in vitro and in vivo phenotypes (% FEV1, %FVC, %FEF25-75, motility, biofilm, virulence, mucoidy, growth in half LB and SCFM) over time.

Table S2. The in vitro phenotypes of *B. cenocepacia* isolates from patients P01 to P16 and the date of isolation.

Table S3. Assembly statistics of Illumina short-read sequencing and PacBio long-read sequencing.

Table S4. Multi-locus sequence type (MLST) of each isolates as identified by SRST2.

Table S5: PacBio identification of methylation motifs.

Table S6. RAPD-specific genes as defined by homologous clusters exlusive to a RAPD type but found in all isolates within the RAPD type

Alkan C, Coe BP, Eichler EE. 2011. Genome structural variation discovery and genotyping. *Nat Rev Genet* **12**: 363-376.

Boissy R, Ahmed A, Janto B, Earl J, Hall BG, Hogg JS, Pusch GD, Hiller LN, Powell E, Hayes J et al. 2011. Comparative supragenomic analyses among the pathogens Staphylococcus aureus, Streptococcus pneumoniae, and Haemophilus influenzae using a modification of the finite supragenome model. *BMC Genomics* **12**: 187.

Boisvert S, Raymond F, Godzaridis E, Laviolette F, Corbeil J. 2012. Ray Meta: scalable de novo metagenome assembly and profiling. *Genome Biol* **13**: R122.

Bolger AM, Lohse M, Usadel B. 2014. Trimmomatic: a flexible trimmer for Illumina sequence data. *Bioinformatics* **30**: 2114-2120.

Bradnam KR, Fass JN, Alexandrov A, Baranay P, Bechner M, Birol I, Boisvert S, Chapman JA, Chapuis G, Chikhi R et al. 2013. Assemblathon 2: evaluating de novo methods of genome assembly in three vertebrate species. *Gigascience* **2**: 10.

Buchmann K. 2014. Evolution of Innate Immunity: Clues from Invertebrates via Fish to Mammals. *Front Immunol* **5**: 459.

Butler J, MacCallum I, Kleber M, Shlyakhter IA, Belmonte MK, Lander ES, Nusbaum C, Jaffe DB. 2008. ALLPATHS: de novo assembly of whole-genome shotgun microreads. *Genome Res* **18**: 810-820.

Chin CS, Alexander DH, Marks P, Klammer AA, Drake J, Heiner C, Clum A, Copeland A, Huddleston J, Eichler EE et al. 2013. Nonhybrid, finished microbial genome assemblies from long-read SMRT sequencing data. *Nat Methods* **10**: 563-569.

Christensen-Dalsgaard M, Jorgensen MG, Gerdes K. 2010. Three new RelE-homologous mRNA interferases of Escherichia coli differentially induced by environmental stresses. *Mol Microbiol* **75**: 333-348.

Conway BA, Venu V, Speert DP. 2002. Biofilm formation and acyl homoserine lactone production in the Burkholderia cepacia complex. *J Bacteriol* **184**: 5678-5685.

Darling AE, Mau B, Perna NT. 2010. progressiveMauve: multiple genome alignment with gene gain, loss and rearrangement. *PLoS One* **5**: e11147.

Dubarry N, Pasta F, Lane D. 2006. ParABS systems of the four replicons of Burkholderia cenocepacia: new chromosome centromeres confer partition specificity. *J Bacteriol* **188**: 1489-1496.

Enright AJ, Van Dongen S, Ouzounis CA. 2002. An efficient algorithm for large-scale detection of protein families. *Nucleic Acids Res* **30**: 1575-1584.

Eutsey RA, Powell E, Dordel J, Salter SJ, Clark TA, Korlach J, Ehrlich GD, Hiller NL. 2015. Genetic Stabilization of the Drug-Resistant PMEN1 Pneumococcus Lineage by Its Distinctive DpnIII Restriction-Modification System. *MBio* **6**: e00173.

Fang G, Munera D, Friedman DI, Mandlik A, Chao MC, Banerjee O, Feng Z, Losic B, Mahajan MC, Jabado OJ et al. 2012. Genome-wide mapping of methylated adenine residues in pathogenic Escherichia coli using single-molecule real-time sequencing. *Nature biotechnology* **30**: 1232-1239.

Feldman M, Bryan R, Rajan S, Scheffler L, Brunnert S, Tang H, Prince A. 1998. Role of flagella in pathogenesis of Pseudomonas aeruginosa pulmonary infection. *Infect Immun* **66**: 43-51.

Folkesson A, Jelsbak L, Yang L, Johansen HK, Ciofu O, Høiby N, Molin S. 2012. Adaptation of Pseudomonas aeruginosa to the cystic fibrosis airway: an evolutionary perspective. *Nature Reviews Microbiology* **10**: 841-851.

Fu L, Niu B, Zhu Z, Wu S, Li W. 2012. CD-HIT: accelerated for clustering the next-generation sequencing data. *Bioinformatics* **28**: 3150-3152.

Goldberg JB, Ganesan S, Comstock AT, Zhao Y, Sajjan US. 2011. Cable pili and the associated 22 kDa adhesin contribute to Burkholderia cenocepacia persistence in vivo. *PLoS One* **6**: e22435.

Goris J, Konstantinidis KT, Klappenbach JA, Coenye T, Vandamme P, Tiedje JM. 2007. DNA-DNA hybridization values and their relationship to whole-genome sequence similarities. *Int J Syst Evol Microbiol* **57**: 81-91.

Hansen CH, Endres RG, Wingreen NS. 2008. Chemotaxis in Escherichia coli: a molecular model for robust precise adaptation. *PLoS Comput Biol* **4**: e1.

Hogg JS, Hu FZ, Janto B, Boissy R, Hayes J, Keefe R, Post JC, Ehrlich GD. 2007. Characterization and modeling of the Haemophilus influenzae core and supragenomes based on the complete genomic sequences of Rd and 12 clinical nontypeable strains. *Genome Biol* **8**: R103.

Holden MT, Seth-Smith HM, Crossman LC, Sebaihia M, Bentley SD, Cerdeno-Tarraga AM, Thomson NR, Bason N, Quail MA, Sharp S et al. 2009. The genome of Burkholderia cenocepacia J2315, an epidemic pathogen of cystic fibrosis patients. *J Bacteriol* **191**: 261-277.

Hunt M, De Silva N, Otto TD, Parkhill J, Keane JA, Harris SR. 2015. Circlator: automated circularization of genome assemblies using long sequencing reads. *bioRxiv* doi:10.1101/023408.

Hurley JM, Woychik NA. 2009. Bacterial toxin HigB associates with ribosomes and mediates translation-dependent mRNA cleavage at A-rich sites. *J Biol Chem* **284**: 18605-18613.

Inouye M, Dashnow H, Raven LA, Schultz MB, Pope BJ, Tomita T, Zobel J, Holt KE. 2014. SRST2: Rapid genomic surveillance for public health and hospital microbiology labs. *Genome Med* **6**: 90.

Konstantinidis KT, Tiedje JM. 2005. Genomic insights that advance the species definition for prokaryotes. *Proc Natl Acad Sci U S A* **102**: 2567-2572.

Koren S, Treangen TJ, Hill CM, Pop M, Phillippy AM. 2014. Automated ensemble assembly and validation of microbial genomes. *BMC Bioinformatics* **15**: 126.

Kurtz S, Phillippy A, Delcher AL, Smoot M, Shumway M, Antonescu C, Salzberg SL. 2004. Versatile and open software for comparing large genomes. *Genome Biol* **5**: R12.

Li H, Durbin R. 2009. Fast and accurate short read alignment with Burrows-Wheeler transform. *Bioinformatics* **25**: 1754-1760.

Li H, Handsaker B, Wysoker A, Fennell T, Ruan J, Homer N, Marth G, Abecasis G, Durbin R, Genome Project Data Processing S. 2009. The Sequence Alignment/Map format and SAMtools. *Bioinformatics* **25**: 2078-2079.

Lieberman TD, Flett KB, Yelin I, Martin TR, McAdam AJ, Priebe GP, Kishony R. 2014. Genetic variation of a bacterial pathogen within individuals with cystic fibrosis provides a record of selective pressures. *Nat Genet* **46**: 82-87.

Lieberman TD, Michel JB, Aingaran M, Potter-Bynoe G, Roux D, Davis MR, Jr., Skurnik D, Leiby N, LiPuma JJ, Goldberg JB et al. 2011. Parallel bacterial evolution within multiple patients identifies candidate pathogenicity genes. *Nat Genet* **43**: 1275-1280.

Liu B, Yuan J, Yiu SM, Li Z, Xie Y, Chen Y, Shi Y, Zhang H, Li Y, Lam TW et al. 2012. COPE: an accurate k-mer-based pair-end reads connection tool to facilitate genome assembly. *Bioinformatics* **28**: 2870-2874.

Loytynoja A. 2014. Phylogeny-aware alignment with PRANK. *Methods Mol Biol* **1079**: 155-170.

Mahenthiralingam E, Campbell ME, Speert DP. 1994. Nonmotility and phagocytic resistance of Pseudomonas aeruginosa isolates from chronically colonized patients with cystic fibrosis. *Infect Immun* **62**: 596-605.

Marinus MG, Casadesus J. 2009. Roles of DNA adenine methylation in host-pathogen interactions: mismatch repair, transcriptional regulation, and more. *FEMS microbiology reviews* **33**: 488-503.

Mruk I, Kobayashi I. 2014. To be or not to be: regulation of restriction-modification systems and other toxin-antitoxin systems. *Nucleic Acids Res* **42**: 70-86.

Murphy MP, Caraher E. 2015. Residence in biofilms allows Burkholderia cepacia complex (Bcc) bacteria to evade the antimicrobial activities of neutrophil-like dHL60 cells. *Pathog Dis* **73**.

O'Toole GA, Kolter R. 1998. Initiation of biofilm formation in Pseudomonas fluorescens WCS365 proceeds via multiple, convergent signalling pathways: a genetic analysis. *Mol Microbiol* **28**: 449-461.

Page AJ, Cummins CA, Hunt M, Wong VK, Reuter S, Holden MT, Fookes M, Falush D, Keane JA, Parkhill J. 2015. Roary: rapid large-scale prokaryote pan genome analysis. *Bioinformatics* doi:10.1093/bioinformatics/btv421.

Palmer KL, Aye LM, Whiteley M. 2007. Nutritional cues control Pseudomonas aeruginosa multicellular behavior in cystic fibrosis sputum. *J Bacteriol* **189**: 8079-8087.

Powers JG, Weigman VJ, Shu J, Pufky JM, Cox D, Hurban P. 2013. Efficient and accurate whole genome assembly and methylome profiling of E. coli. *BMC Genomics* **14**: 675.

R Core Team. 2016. R: A Language and Environment for Statistical Computing. R Foundation for Statistical Computing, Vienna, Austria.

Richter M, Rossello-Mora R. 2009. Shifting the genomic gold standard for the prokaryotic species definition. *Proc Natl Acad Sci U S A* **106**: 19126-19131.

Sajjan U, Wu Y, Kent G, Forstner J. 2000. Preferential adherence of cable-piliated burkholderia cepacia to respiratory epithelia of CF knockout mice and human cystic fibrosis lung explants. *J Med Microbiol* **49**: 875-885.

Sajjan US, Sun L, Goldstein R, Forstner JF. 1995. Cable (cbl) type II pili of cystic fibrosis-associated Burkholderia (Pseudomonas) cepacia: nucleotide sequence of the cblA major subunit pilin gene and novel morphology of the assembled appendage fibers. *J Bacteriol* **177**: 1030-1038.

Schulz H, Fabianek RA, Pellicioli EC, Hennecke H, Thony-Meyer L. 1999. Heme transfer to the heme chaperone CcmE during cytochrome c maturation requires the CcmC protein, which may function independently of the ABC-transporter CcmAB. *Proc Natl Acad Sci U S A* **96**: 6462-6467.

Seed KD, Dennis JJ. 2008. Development of Galleria mellonella as an alternative infection model for the Burkholderia cepacia complex. *Infect Immun* **76**: 1267-1275.

Seemann T. 2014. Prokka: rapid prokaryotic genome annotation. *Bioinformatics* **30**: 2068-2069.

Silva IN, Ferreira AS, Becker JD, Zlosnik JE, Speert DP, He J, Mil-Homens D, Moreira LM. 2011. Mucoid morphotype variation of Burkholderia multivorans during chronic cystic fibrosis lung infection is correlated with changes in metabolism, motility, biofilm formation and virulence. *Microbiology (Reading, England)* **157**: 3124-3137.

Smith EE, Buckley DG, Wu Z, Saenphimmachak C, Hoffman LR, D'Argenio DA, Miller SI, Ramsey BW, Speert DP, Moskowitz SM et al. 2006. Genetic adaptation by Pseudomonas aeruginosa to the airways of cystic fibrosis patients. *Proc Natl Acad Sci U S A* **103**: 8487-8492.

Srikhanta YN, Fox KL, Jennings MP. 2010. The phasevarion: phase variation of type III DNA methyltransferases controls coordinated switching in multiple genes. *Nat Rev Microbiol* **8**: 196-206.

Stamatakis A. 2014. RAxML version 8: a tool for phylogenetic analysis and post-analysis of large phylogenies. *Bioinformatics* **30**: 1312-1313.

Taylor BL, Zhulin IB, Johnson MS. 1999. Aerotaxis and other energy-sensing behavior in bacteria. *Annu Rev Microbiol* **53**: 103-128.

Thompson O, Edgley M, Strasbourger P, Flibotte S, Ewing B, Adair R, Au V, Chaudhry I, Fernando L, Hutter H et al. 2013. The million mutation project: a new approach to genetics in Caenorhabditis elegans. *Genome Res* **23**: 1749-1762.

Tomich M, Herfst CA, Golden JW, Mohr CD. 2002. Role of flagella in host cell invasion by Burkholderia cepacia. *Infect Immun* **70**: 1799-1806.

Uehlinger S, Schwager S, Bernier SP, Riedel K, Nguyen DT, Sokol PA, Eberl L. 2009. Identification of specific and universal virulence factors in Burkholderia cenocepacia strains by using multiple infection hosts. *Infect Immun* **77**: 4102-4110.

Urban TA, Griffith A, Torok AM, Smolkin ME, Burns JL, Goldberg JB. 2004. Contribution of Burkholderia cenocepacia flagella to infectivity and inflammation. *Infect Immun* **72**: 5126-5134.

Van Acker H, Sass A, Bazzini S, De Roy K, Udine C, Messiaen T, Riccardi G, Boon N, Nelis HJ, Mahenthiralingam E et al. 2013. Biofilm-grown Burkholderia cepacia complex cells survive antibiotic treatment by avoiding production of reactive oxygen species. *PLoS One* **8**: e58943.

Van Leene J, Stals H, Eeckhout D, Persiau G, Van De Slijke E, Van Isterdael G, De Clercq A, Bonnet E, Laukens K, Remmerie N et al. 2007. A tandem affinity purification-based technology platform to study the cell cycle interactome in Arabidopsis thaliana. *Mol Cell Proteomics* **6**: 1226-1238.

Vazquez-Juarez RC, Kuriakose JA, Rasko DA, Ritchie JM, Kendall MM, Slater TM, Sinha M, Luxon BA, Popov VL, Waldor MK et al. 2008. CadA negatively regulates Escherichia coli O157:H7 adherence and intestinal colonization. *Infect Immun* **76**: 5072-5081.

Wright ES. 2015. DECIPHER: harnessing local sequence context to improve protein multiple sequence alignment. *BMC Bioinformatics* **16**: 322.

Yang K, Meng J, Huang YC, Ye LH, Li GJ, Huang J, Chen HM. 2014. The role of the QseC quorum-sensing sensor kinase in epinephrine-enhanced motility and biofilm formation by Escherichia coli. *Cell Biochem Biophys* **70**: 391-398.

Zak M, Mendonca R, Balazs M, Barrett K, Bergeron P, Blair WS, Chang C, Deshmukh G, Devoss J, Dragovich PS et al. 2012. Discovery and optimization of C-2 methyl imidazopyrrolopyridines as potent and orally bioavailable JAK1 inhibitors with selectivity over JAK2. *J Med Chem* **55**: 6176-6193.

Zlosnik JE, Costa PS, Brant R, Mori PY, Hird TJ, Fraenkel MC, Wilcox PG, Davidson AG, Speert DP. 2011a. Mucoid and nonmucoid Burkholderia cepacia complex bacteria in cystic fibrosis infections. *American journal of respiratory and critical care medicine* **183**: 67-72.

Zlosnik JE, Mori PY, To D, Leung J, Hird TJ, Speert DP. 2014. Swimming motility in a longitudinal collection of clinical isolates of Burkholderia cepacia complex bacteria from people with cystic fibrosis. *PLoS One* **9**: e106428.

Zlosnik JE, Speert DP. 2010. The role of mucoidy in virulence of bacteria from the Burkholderia cepacia complex: a systematic proteomic and transcriptomic analysis. *J Infect Dis* **202**: 770-781.

Zlosnik JE, Zhou G, Brant R, Henry DA, Hird TJ, Mahenthiralingam E, Chilvers MA, Wilcox P, Speert DP. 2015. Burkholderia species infections in patients with cystic fibrosis in British Columbia, Canada. 30 years' experience. *Ann Am Thorac Soc* **12**: 70-78.

Zlosnik JEA, Costa PS, Brant R, Mori PYB, Hird TJ, Fraenkel MC, Wilcox PG, Davidson AGF, Speert DP. 2011b. Mucoid and Nonmucoid Burkholderia cepaciaComplex Bacteria in Cystic Fibrosis Infections. *American journal of respiratory and critical care medicine* **183**: 67-72.

Zweiger G, Marczynski G, Shapiro L. 1994. A Caulobacter DNA methyltransferase that functions only in the predivisional cell. *J Mol Biol* **235**: 472-485.
